# Association Between Intravenous Magnesium Therapy in the Emergency Department and Subsequent Hospitalization Among Pediatric Patients With Refractory Acute Asthma: Secondary Analysis of a Randomized Clinical Trial

**DOI:** 10.1001/jamanetworkopen.2021.17542

**Published:** 2021-07-19

**Authors:** Suzanne Schuh, Stephen B. Freedman, Roger Zemek, Amy C. Plint, David W. Johnson, Francine Ducharme, Jocelyn Gravel, Graham Thompson, Sarah Curtis, Derek Stephens, Allan L. Coates, Karen J. Black, Darcy Beer, Judy Sweeney, Maggie Rumantir, Yaron Finkelstein

**Affiliations:** 1 Division of Pediatric Emergency Medicine, The Hospital for Sick Children, University of Toronto, Toronto, Ontario, Canada; 2 Sick Kids Research Institute, The Hospital for Sick Children, University of Toronto, Toronto, Ontario, Canada; 3 Section of Pediatric Emergency Medicine, Department of Pediatrics, Alberta Children’s Hospital Research Institute, Cumming School of Medicine, University of Calgary, Calgary, Alberta, Canada; 4 Section of Gastroenterology, Department of Emergency Medicine, Alberta Children’s Hospital Research Institute, Cumming School of Medicine, University of Calgary, Calgary, Alberta, Canada; 5 Department of Pediatrics, Children’s Hospital of Eastern Ontario, University of Ottawa, Ottawa, Ontario, Canada; 6 Department of Emergency Medicine, Children’s Hospital of Eastern Ontario, University of Ottawa, Ottawa, Ontario, Canada; 7 CHEO Research Institute, Ottawa, Ontario, Canada; 8 Department of Pediatrics, Alberta Children’s Hospital, Calgary, Alberta, Canada; 9 Department of Emergency Medicine, Alberta Children’s Hospital, Calgary, Alberta, Canada; 10 Department of Physiology & Pharmacology, Alberta Children’s Hospital, Calgary, Alberta, Canada; 11 Alberta Children’s Hospital Research Institute, Cumming School of Medicine, University of Calgary, Calgary, Alberta, Canada; 12 Department of Pediatric Emergency Medicine, Centre Hospitalier Universitaire Sainte-Justine, Montréal, Quebec, Canada; 13 Department of Pediatrics, Université de Montréal, Montréal, Quebec, Canada; 14 Department of Social and Preventive Medicine, Université de Montréal, Montréal, Quebec, Canada; 15 Department of Pediatrics, Alberta Children’s Hospital, University of Calgary, Calgary, Alberta, Canada; 16 Department of Emergency Medicine, Alberta Children’s Hospital, University of Calgary, Calgary, Alberta, Canada; 17 Alberta Children’s Hospital Research Institute, University of Calgary, Calgary, Alberta, Canada; 18 Department of Pediatrics, Stollery Children’s Hospital, University of Alberta, Edmonton, Alberta, Canada; 19 Department of Emergency Medicine, Stollery Children’s Hospital, University of Alberta, Edmonton, Alberta, Canada; 20 Department of Pediatrics, Clinical Research Services, Sick Kids Research Institute, The Hospital for Sick Children, Toronto, Ontario, Canada; 21 Division of Respiratory Medicine, Sick Kids Research Institute, The Hospital for Sick Children, University of Toronto, Toronto, Ontario, Canada; 22 Division of Pediatric Emergency Medicine, British Columbia Children’s Hospital, University of British Columbia, Vancouver, British Columbia, Canada; 23 Department of Pediatrics and Child Health, Division of Pediatric Emergency Medicine, University of Manitoba, Winnipeg, Manitoba, Canada; 24 Children’s Hospital Research Institute of Manitoba, Winnipeg, Manitoba, Canada; 25 Division of Pediatric Emergency Medicine, The Hospital for Sick Children, University of Toronto, Toronto, Ontario, Canada; 26 Division of Clinical Pharmacology and Toxicology, The Hospital for Sick Children, University of Toronto, Toronto, Ontario, Canada

## Abstract

**Importance:**

Despite guidelines recommending administration of intravenous (IV) magnesium sulfate for refractory pediatric asthma, the number of asthma-related hospitalizations has remained stable, and IV magnesium therapy is independently associated with hospitalization.

**Objective:**

To examine the association between IV magnesium therapy administered in the emergency department (ED) and subsequent hospitalization among pediatric patients with refractory acute asthma after adjustment for patient-level variables.

**Design, Setting, and Participants:**

This post hoc secondary analysis of a double-blind randomized clinical trial of children with acute asthma treated from September 26, 2011, to November 19, 2019, at 7 Canadian tertiary care pediatric EDs was conducted between September and November 2020. In the randomized clinical trial, 816 otherwise healthy children aged 2 to 17 years with Pediatric Respiratory Assessment Measure (PRAM) scores of 5 points or higher after initial therapy with systemic corticosteroids and inhaled albuterol with ipratropium bromide were randomly assigned to 3 nebulized treatments of albuterol plus either magnesium sulfate or 5.5% saline placebo.

**Exposures:**

Intravenous magnesium sulfate therapy (40-75 mg/kg).

**Main Outcomes and Measures:**

The association between IV magnesium therapy in the ED and subsequent hospitalization for asthma was assessed using multivariable logistic regression analysis. Analyses were adjusted for year epoch at enrollment, receipt of IV magnesium, PRAM score after initial therapy and at ED disposition, age, sex, duration of respiratory distress, previous intensive care unit admission for asthma, hospitalizations for asthma within the past year, atopy, and receipt of oral corticosteroids within 48 hours before arrival in the ED, nebulized magnesium, and additional albuterol after inhaled magnesium or placebo, with site as a random effect.

**Results:**

Among the 816 participants, the median age was 5 years (interquartile range, 3-7 years), 517 (63.4%) were boys, and 364 (44.6%) were hospitalized. A total of 215 children (26.3%) received IV magnesium, and 190 (88.4%) of these children were hospitalized compared with 174 of 601 children (29.0%) who did not receive IV magnesium. Multivariable factors associated with hospitalization were IV magnesium receipt from 2011 to 2016 (odds ratio [OR], 22.67; 95% CI, 6.26-82.06; *P* < .001) and from 2017 to 2019 (OR, 4.19; 95% CI, 1.99-8.86; *P* < .001), use of additional albuterol (OR, 5.94; 95% CI, 3.52-10.01; *P* < .001), and increase in PRAM score at disposition (per 1-U increase: OR, 2.24; 95% CI, 1.89-2.65; *P* < .001). In children with a disposition PRAM score of 3 or lower, receipt of IV magnesium therapy was associated with hospitalization (OR, 8.52; 95% CI, 2.96-24.41; *P* < .001).

**Conclusions and Relevance:**

After adjustment for patient-level characteristics, receipt of IV magnesium therapy after initial asthma treatment in the ED was associated with subsequent hospitalization. This association also existed among children with mild asthma at ED disposition. Evidence of a benefit of IV magnesium regarding hospitalization may clarify its use in the treatment of refractory pediatric asthma.

**Trial registration:**

ClinicalTrials.gov: NCT01429415

## Key Points

**Question:**

Is receipt of intravenous (IV) magnesium sulfate therapy in the emergency department associated with subsequent hospitalization among children with refractory acute asthma after adjustment for patient-level characteristics?

**Findings:**

In this secondary analysis of a randomized clinical trial of 816 children who experienced moderate to severe respiratory distress after stabilization therapy and inhaled magnesium therapy or placebo, 88.4% of those who received IV magnesium therapy were hospitalized compared with 29.0% of those who did not receive this therapy. After adjustment, children who received IV magnesium therapy had greater odds of hospitalization compared with those who did not receive this therapy.

**Meaning:**

The findings suggest that, after adjustment of patient-level characteristics, receipt of IV magnesium in the emergency department is associated with subsequent hospitalization among children with asthma.

## Teaser

This cohort study assesses the association between intravenous magnesium therapy administered in the emergency department and subsequent hospitalization among pediatric patients with refractory acute asthma in Canada.

## Introduction

Acute asthma exacerbations are a leading cause of pediatric emergency department (ED) visits and related hospitalizations, with associated costs of approximately $4 billion annually in the US.^1^ Although systemic corticosteroids, inhaled β_2_ agonists, and anticholinergics are recommended for the management of severe exacerbations, some children are resistant to this therapy, in part owing to genetic polymorphisms.^2,3,4^ For children experiencing ongoing respiratory distress after initial management, intravenous (IV) magnesium therapy represents a potential second-line treatment option.

Almost all international asthma management guidelines recommend consideration of IV magnesium as ancillary therapy in children with severe acute asthma.^5,6,7,8,9^ However, the evidence of IV magnesium benefit is, to our knowledge, limited to disparate results from 3 small randomized clinical trials (RCTs) (a total of 115 patients).^10,11,12^ A decade ago, IV magnesium was generally not used to prevent asthma-related hospitalizations,^13^ and only 12% of children hospitalized for asthma received IV magnesium in the ED.^14^ A recent Pediatric Emergency Care Applied Research Network Registry report^15^ found that only 10% of children treated for asthma in the ED were treated with IV magnesium. A 2017 report from the Pediatric Health Information System Data Collaborative^16^ found that although the use of IV magnesium was increasing, 36% of children hospitalized for asthma received IV magnesium. Neither study found an association between IV magnesium and lower hospitalization rates or lower likelihood of being treated in the intensive care unit.^15,16^

The findings in those 2 studies^15,16^ may be explained by the administration of IV magnesium to children with more severe asthma, who are more refractory to treatment. Moreover, the initial severity of illness among those treated with IV magnesium may be associated with reduced likelihood of ED discharge despite satisfactory clinical improvement. To our knowledge, no previous study has explored the real-life association between IV magnesium use, clinical asthma severity over time, and hospitalization.

To explore the association between asthma severity, IV magnesium therapy, and hospitalization among children with refractory acute asthma, we conducted a secondary analysis of a recent multicenter RCT.^17^ The trial found that the administration of nebulized magnesium sulfate with albuterol to children who continued to experience moderate to severe respiratory distress after standardized initial therapy did not convey any benefit compared with placebo with albuterol.^17^ Because many children in the trial also received IV magnesium after inhaled magnesium or placebo and asthma severity was measured using the validated Pediatric Respiratory Assessment Measure (PRAM) score,^18^ the trial offered a unique opportunity to assess the association between receipt of IV magnesium therapy in the ED and subsequent hospitalization. We hypothesized that children administered IV magnesium would be more likely to be hospitalized compared with those not given IV magnesium after adjustment for patient-level variables.

## Methods

### Study Design and Population

We conducted a post hoc secondary analysis of a multicenter, double-blind, placebo-controlled RCT (NCT01429415)^17^ of children aged 2 to 17 years treated at 7 Pediatric Emergency Research Canada EDs for acute asthma from September 26, 2011, to November 19, 2019 (the trial protocol for the RCT is given in Supplement 1). This secondary analysis was performed from September to November 2020. The research ethics boards of the Hospital for Sick Children and of all participating sites approved the RCT and all data obtained during the study. Waiver of informed consent was not obtained for this secondary analysis because the parent trial included collection of all data used in this secondary analysis, and the consent form specified intravenous magnesium as a possible required treatment during trial participation. This study followed the Strengthening the Reporting of Observational Studies in Epidemiology (STROBE) reporting guideline.

Children enrolled in the RCT who continued to experience moderate to severe respiratory distress,^19,20^ defined by a PRAM score of 5 points or higher^19^ (Table 1)^21^ after 1 hour of standardized initial treatment with systemic corticosteroids and 3 treatments with inhaled albuterol and ipratropium bromide, were randomly assigned to receive three 20-minute treatments of nebulized albuterol (5 mg) plus either nebulized magnesium sulfate (600 mg) or 5.5% saline placebo via a pretested, high-efficiency Aeroneb Go nebulizer (Philips).^22,23^ After completion of this therapy (60 minutes after randomization), the ED physicians prescribed additional treatments such as IV magnesium sulfate as needed and decided whether to hospitalize patients according to their clinical judgement.

**Table 1.** Pediatric Respiratory Assessment Measure Score^a^

Sign	Score^b^			
	0	1	2	3
Suprasternal retractions	Absent	NA	Present	NA
Scalene muscle contraction	Absent	NA	Present	NA
Air entry	Normal	Decreased at bases	Widespread decrease	Absent or minimal
Wheezing	Absent	Expiratory only	Inspiratory and expiratory	Audible without stethoscope or silent chest with minimal air entry
Oxygen saturation, %	≥95	92-94	≤91	NA
Oxygen saturation, Calgary, %^c^	≥93	90-92	≤89	NA

Abbreviation: NA, not applicable.

^a^ Adapted from *The Journal of Pediatrics*, 2008;137(6):762-768, ©2008, with permission from Elsevier.^18^

^b^ Score represents the sum of the individual components (range, 0-12).^18^

^c^ Because Calgary is 1000 m above sea level, the saturation cutoffs were adjusted accordingly.^21^

The RCT included children with a physician’s diagnosis of asthma or a previous wheeze treated with inhaled bronchodilators or systemic corticosteroids. Children with comorbidities such as chronic lung disease, cardiovascular disease, kidney disease, neurologic disease, or other systemic diseases were excluded, as were those experiencing their first episode of asthmalike symptoms who may have had other diagnoses. Children needing immediate airway stabilization and those who received IV magnesium therapy before trial enrollment were also excluded.

### Exposure

This secondary analysis used data from patients enrolled in the RCT who were administered IV magnesium after receipt of nebulized magnesium or placebo before disposition from the ED based on the clinical judgment of the treating ED physician. Magnesium was given by a continuous 30-minute IV infusion using magnesium doses of 40 to 75 mg/kg (maximum 2 g) in accordance with the local standard of care.

### Asthma Severity Measurement

Trained study nurses measured baseline PRAM scores and vital signs after initial stabilization therapy and then hourly up to 240 minutes after the start of the study intervention or until the time of ED disposition. The PRAM score^18^ is a summative clinical assessment instrument for children with acute asthma that ranges from 0 to 12 points, with scores of 1 to 3 indicating mild asthma; 4 to 7, moderate asthma; and 8 to 12, severe asthma. The score has good interobserver reliability across all age groups,^18^ distinguishes between severity levels,^24^ and has been successfully used as a trial outcome.^25^ To our knowledge, it is the only acute asthma severity score with demonstrated criterion validity using respiratory resistance as the gold standard.^26^ Based on previous literature,^18,27^ a PRAM score of 3 points or lower indicates mild asthma consistent with eligibility for discharge home. Therefore, we used this PRAM cutoff to identify children suitable for discharge home.

To ensure the fidelity of PRAM measurement, all nurses participating in the research completed an online training module.^28,29^ Study nurses were also trained in standard interviewing techniques and documented patient-level demographic, historical, and clinical data, including information on IV magnesium administration, disposition from the ED, and revisits to any medical facility within 72 hours.

### Study Outcomes

The primary outcome for this secondary analysis was hospitalization for asthma at the index ED visit. This outcome was selected because hospitalization is an important health utilization outcome, accounting for half of pediatric asthma-related costs.^30^ Hospitalization is also important to families because it usually requires caregivers to take time off work and arrange alternative sources of care for their other children.

### Statistical Analysis

To provide 80% power at a 2-sided significance level of 5%, using 20 patients with the outcome of interest for each of the 15 a priori–defined variables examined and knowing that 50% of children enrolled in the original study were admitted to the hospital,^17^ a sample of at least 300 hospitalized patients was needed for this study.^31^ We used proportions and 95% CIs to describe categorical data and means with SDs or medians with interquartile ranges (IQRs) for continuous data.

A priori–defined variables selected for the multivariate logistic regression analysis because of their plausible association with hospitalization included treatment with IV magnesium in the ED (exposure) and the PRAM score measured after initial stabilization therapy (at randomization) and at disposition from the ED as a marker of treatment response. The following patient-level variables that may have been independently associated with asthma severity and disposition were also included: age,^32,33^ sex,^34^ duration of respiratory distress before arrival,^35^ previous intensive care unit admission for asthma, asthma-related hospitalizations within the past year,^36^ personal history of atopy (eczema or allergic rhinitis), history of atopy in parents and siblings, oral corticosteroid administration within the 48 hours before ED arrival, intervention with nebulized magnesium plus albuterol (vs placebo plus albuterol) as part of the RCT intervention, and additional inhaled albuterol treatments given in the ED from the end of administration of the experimental therapy to 240 minutes after administration of therapy (≥1 treatment vs none). Because the proportion of patients hospitalized after administration of IV magnesium may have changed during the course of the original study, we included the year of presentation as another variable. Because only 2 centers participated between 2011 and 2014 (with a small number of participants), we divided the study periods into the 2011-2016 and 2017-2019 calendar-year epochs.

For the primary outcome, a multivariable logistic regression analysis was performed to assess the association between hospital admission after the ED visit as a binary dependent variable and IV magnesium therapy receipt in the ED. Because the association between hospitalization and receipt of IV magnesium therapy may have differed between the calendar-year epochs, we examined the interaction between the epochs and receipt of IV magnesium. Because management was likely similar within individual EDs, the ED site was included as a random effect. Goodness of fit of the model was examined with the Hosmer-Lemeshow test.

The secondary analysis was limited to children with a PRAM score of 3 points or lower at the time of ED disposition. In this subpopulation, we used multiple logistic regression analysis to examine the association between hospital admission after the ED visit and receipt of IV magnesium therapy. Given that only 72 children in this subpopulation were hospitalized and because the association between year epoch and administration of IV magnesium was examined, we limited the adjustment to the variables with the highest clinical interest: the year epochs 2011-2016 and 2017-2019, age, PRAM score measured at randomization and at ED disposition, treatment with inhaled magnesium, and additional inhaled albuterol treatments given in the ED between the end of administration of the experimental therapy and 240 minutes after administration of therapy, with the ED site as a random effect. Missing data were managed using listwise deletion because the number of patients with missing data was minimal (<5%); no imputations were performed. Statistical analysis was performed using SAS, version 9.4 (SAS Institute Inc). Significance was set at 2-sided *P* < .05.

## Results

A total of 5846 patients were screened for the RCT^17^; 5028 were excluded or refused participation, 818 were included in the randomization, and 816 constituted the study population for the RCT (409 received inhaled magnesium, and 407 received inhaled placebo) and for this secondary analysis. The median age was 5 years (IQR, 3-7 years), 517 participants (63.4%) were boys, and the median baseline PRAM score was 6 points (IQR, 5-7 points).

A total of 364 of the 816 study patients (44.6%) were hospitalized for asthma at the index ED visit, and 452 (55.4%) were discharged home from the ED. A total of 215 patients (26.3%) received IV magnesium in the ED, including 190 of the 364 patients (52.2%) who were hospitalized. The site-level rate of treatment with IV magnesium ranged from 6.3% to 33.7%. Children who received IV magnesium had higher PRAM scores after initial stabilization therapy (mean [SD], 6.7 [1.4] points vs 6.0 [1.1] points) and a higher likelihood of previous admissions for asthma to the intensive care unit (26 of 215 patients [12.1%] vs 53 of 601 patients [8.8%]) compared with those not administered IV magnesium (Table 2). Among children given IV magnesium, the mean (SD) PRAM score decreased from 5.39 (1.68) points at 60 minutes after randomization (when IV magnesium could first be administered) to 5.24 (1.89) points at ED disposition (mean [SD] change, −0.15 [1.70] points). Of the 215 children given IV magnesium, 190 (88.4%) were admitted to the hospital at the index ED visit. Of the 25 children treated with IV magnesium who were discharged home, 22 (88.0%) were treated at a single site, and 0.0% (95% CI, 0.0%-12.5%) of these children presented for further asthma care to any medical facility within the subsequent 72 hours.^37^

**Table 2.** Characteristics of Patients Who Did and Did Not Receive IV Magnesium Therapy

Characteristic	Patients			Unadjusted OR (95% CI)
	Total No. (N = 816)	Received IV magnesium, No. (%) (n = 215)	Did not receive IV magnesium, No. (%) (n = 601)	
Year of index ED visit				
2011-2016	386	102 (47.4)	284 (47.3)	1.08 (0.78-1.49)
2017-2019	430	113 (52.6)	317 (52.7)	
Initial PRAM score, mean (SD), points^a^	816	6.7 (1.4)	6.0 (1.1)	1.52 (1.35-1.72)^b^
ED disposition PRAM score, mean (SD), points	785	5.2 (1.9)	3.0 (1.7)	1.96 (1.75-2.19)^c^
Age ≤5 y	503	120 (55.8)	383 (63.7)	0.74 (0.54-1.02)
Sex				
Male	517	134 (62.3)	383 (63.7)	0.92 (0.66-1.28)
Female	299	81 (37.7)	218 (36.3)	
Respiratory distress ≤12 h before arrival	321	83 (38.6)	238 (39.6)	0.99 (0.71-1.37)
Past ICU asthma admission	79	26 (12.1)	53 (8.8)	1.88 (1.11-3.17)
Hospitalization for asthma in past year	197	64 (29.8)	133 (22.1)	1.48 (1.03-2.12)
Atopy	493	131 (60.9)	362 (60.2)	1.05 (0.75-1.46)
Family history of atopy	603	168 (78.1)	435 (72.4)	1.27 (0.87-1.88)
Oral corticosteroid therapy before arrival	145	39 (18.1)	106 (17.6)	1.31 (0.85-2.02)
Nebulized magnesium therapy	409	100 (46.5)	309 (51.4)	0.81 (0.61-1.07)
≥1 Albuterol treatment after experimental therapy	510	187 (87.0)	323 (53.7)	7.32 (4.57-11.70)

Abbreviations: ED, emergency department; ICU, intensive care unit; IV, intravenous; PRAM, Pediatric Respiratory Assessment Measure; OR, odds ratio.

^a^ PRAM score measured after initial therapy with albuterol, ipratropium bromide, and corticosteroids. Table 1 gives score details.

^b^ Per 1-U increase in the initial PRAM score.

^c^ Per 1-U increase in the ED disposition PRAM score.

### Association Between IV Magnesium Therapy and Hospitalization

The overall hospitalization rate for acute asthma among participating EDs ranged from 28.3% to 60.5%. Of 215 children given IV magnesium, 190 (88.4%) were hospitalized, whereas 174 of 601 children (29.0%) who did not receive magnesium were hospitalized. After adjustment, children receiving IV magnesium in the ED had almost 10 times greater odds of being hospitalized compared with those not administered IV magnesium (odds ratio [OR], 9.76; 95% CI, 4.58-20.77; *P* < .001) (Table 3). Furthermore, hospitalization after receipt of IV magnesium therapy was associated with the year epoch of presentation. Specifically, the adjusted odds of hospitalization after IV magnesium therapy from 2011 to 2016 were 22.67 (95% CI, 6.26-82.06; *P* < .001). In contrast, the adjusted odds of hospitalization after IV magnesium therapy from 2017 to 2019 were 4.19 (95% CI, 1.99-8.86; *P* < .001). Therefore, the odds of hospitalization after IV magnesium decreased approximately 5-fold over time. This decrease was statistically significant (OR, 5.40; 95% CI, 1.25-23.33; *P* = .02) (Table 3).

**Table 3.** Association Between Hospitalization and Patient Characteristics

Characteristic	Patients				OR (95% CI)	
	Total No. (N = 816)	Hospitalized, No. (%) (n = 364)	Not hospitalized, No. (%) (n = 452)	Unadjusted		Multivariable^a^
Year of reported treatment						
2011-2016	386	185 (50.8)	201 (44.5)	1.30 (0.98-1.72)		1.80 (0.86-3.72)
2017-2019	430	179 (49.2)	251 (55.5)			
IV magnesium therapy						
Total	215	190 (52.2)	25 (5.5)	25.87 (15.99-41.84)		9.76 (4.58-20.77)
2011-2016	NA	NA	NA	NA		22.67 (6.26-82.06)^b^
2017-2019	NA	NA	NA	NA		4.19 (1.99-8.86)^b^
IV magnesium × year interaction	NA	NA	NA	NA		5.40 (1.25-23.33)
Initial PRAM score, mean (SD)^c^	816	6.6 (1.4)	5.9 (1.0)	1.64 (1.45-1.86)		1.21 (0.99-1.46)^d^
ED disposition PRAM score, mean (SD)	785	4.9 (1.8)	2.5 (1.3)	2.70 (2.35-3.11)		2.24 (1.89-2.65)^e^
Age ≤5 y	503	232 (63.7)	271 (60.0)	1.20 (0.89-1.59)		1.39 (0.86-2.24)
Sex						
Male	517	224 (61.5)	293 (64.8)	1.14 (0.86-1.52)		0.92 (0.57-1.47)
Female	299	140 (38.5)	159 (35.2)			
Duration of symptoms ≤12 h before arrival	321	147 (40.4)	174 (38.5)	1.12 (0.85-1.51)		1.56 (0.73-1.84)
Past ICU admission for asthma	79	46 (12.1)	33 (7.7)	1.74 (1.10-2.80)		1.66 (0.76-3.66)
Hospitalization for asthma in past year	197	103 (28.3)	94 (20.8)	1.58 (1.14-2.20)		0.89 (0.52-1.52)
Atopy	493	214 (58.8)	279 (61.7)	0.82 (0.62-1.10)		0.79 (0.49-1.28)
Family history of atopy	603	272 (74.7)	331 (73.2)	1.10 (0.76-1.47)		1.06 (0.62-1.81)
Oral corticosteroid therapy before arrival	145	74 (20.3)	71 (15.7)	1.36 (0.93-1.98)		1.12 (0.60-2.08)
Nebulized magnesium therapy	409	172 (47.3)	237 (52.4)	0.81 (0.61-1.07)		0.97 (0.62-1.51)
≥1 Albuterol treatment after experimental therapy	510	318 (87.4)	192 (42.5)	11.82 (8.00-17.47)		5.94 (3.52-10.01)

Abbreviations: ED, emergency department; ICU, intensive care unit; IV, intravenous; NA, not applicable; OR, odds ratio; PRAM, Pediatric Respiratory Assessment Measure.

^a^ The multivariable regression analysis was adjusted for all variables shown.

^b^ Adjusted OR for the association between IV magnesium therapy and hospitalization.

^c^ Details of the PRAM score are given in Table 1. PRAM score measured after initial therapy with corticosteroids, salbutamol, and ipratropium bromide.

^d^ Per 1-U increase in initial PRAM score.

^e^ Per 1-U increase in ED disposition PRAM score.

The PRAM score measured at disposition was also independently associated with hospital admission in adjusted analyses (OR, 2.24; 95% CI, 1.89-2.65; *P* < .001), as was additional albuterol treatment administered after the completion of the experimental therapy (OR, 5.94; 95% CI, 3.52-10.01; *P* < .001) (Table 3). The Hosmer-Lemeshow test showed excellent goodness of fit.

### Association Between IV Magnesium Therapy and Hospitalization Among Children With PRAM Scores of 3 Points or Lower

Of the 417 children who had a PRAM score of 3 points or lower^18,26^ at ED disposition, 72 (17.3%) were hospitalized, including 19 of 31 children (61.3%) who received IV magnesium and 53 of 386 (13.7%) who did not receive IV magnesium. In this subpopulation, children treated with IV magnesium had 8.5 times higher odds of hospitalization compared with children not given IV magnesium (multivariable OR, 8.52; 95% CI, 2.96-24.41; *P* < .001) (Table 4). Although these children had 2.5 times lower odds of hospitalization after IV magnesium treatment in the 2017-2019 epoch than in the 2011-2016 epoch (OR, 1.34; 95% CI, 0.48-3.70; *P* = .42) (Table 4), this decrease in odds (interaction term) did not reach statistical significance.

**Table 4.** Association Between IV Magnesium Therapy and Hospitalization Among Children With a PRAM Score of 3 or Lower at ED Disposition

Characteristic	Patients				OR (95% CI)	
	Total No. (N = 417)	Hospitalized, No. (%) (n = 72)	Not hospitalized, No. (%) (n = 345)	Unadjusted		Multivariable^a^
Year of reported treatment						
2011-2016	182	31 (43.1)	151 (43.8)	0.97 (0.58-1.62)		1.34 (0.48-3.70)
2017-2019	235	41 (56.9)	194 (56.2)			
IV magnesium therapy						
Total	31	19 (26.4)	12 (3.5)	13.46 (5.89-30.77)		8.52 (2.96-24.41)
2011-2016	NA	NA	NA	NA		13.02 (2.30-73.74)^b^
2017-2019	NA	NA	NA	NA		5.58 (1.79-17.41)^b^
Initial PRAM score, mean (SD)^c^	417	6.3 (1.4)	5.9 (1.1)	1.46 (1.18-1.81)		1.51 (1.16-1.97)^d^
ED disposition PRAM score, mean (SD)	417	2.5 (0.7)	2.0 (0.9)	2.17 (1.52-3.09)		2.11 (1.38-3.22)^e^
Age ≤5 y	256	51 (71.8)	205 (59.4)	1.72 (0.98-3.01)		1.50 (0.77-2.94)
Nebulized magnesium therapy	211	33 (45.8)	178 (51.6)	1.30 (0.78-2.18)		0.95 (0.52-1.76)
≥1 Albuterol treatment after experimental therapy	192	59 (81.9)	133 (38.6)	7.23 (3.81-13.72)		5.40 (2.71-11.11)

Abbreviations: ED, emergency department; IV, intravenous; NA, not applicable; OR, odds ratio; PRAM, Pediatric Respiratory Assessment Measure.

^a^ The multivariable regression analysis was adjusted for all variables shown.

^b^ Adjusted ORs for the association between IV magnesium therapy and hospitalization.

^c^ Details of the PRAM score are given in Table 1. PRAM score measured after initial therapy with corticosteroids, inhaled albuterol, and ipratropium bromide.

^d^ Per 1-U increase in initial PRAM score.

^e^ Per 1-U increase in ED disposition PRAM score.

## Discussion

In this secondary analysis of a multicenter RCT of children refractory to optimized initial asthma therapy in the ED, we found that after adjustment for patient-level covariates, administration of IV magnesium therapy was associated with subsequent hospitalization, and the odds of hospitalization after receiving IV magnesium decreased over time. Furthermore, children eligible for discharge home after treatment with IV magnesium had 8 times higher odds of hospitalization compared with children who did not receive IV magnesium after adjustment for asthma severity and other patient-level characteristics.

The decision to administer IV magnesium may influence or follow the decision of the ED physicians to request hospital admission. This study’s findings are not consistent with the conclusions of previous systematic reviews,^38,39,40,41,42^ which reported that IV magnesium therapy was associated with a reduction in hospitalizations among children with refractory acute asthma. Several factors may account for this discrepancy. First, IV magnesium therapy is a second-line intervention typically reserved for patients with more sever illness and therefore may be associated with a low threshold for hospitalization. Second, the half-life of IV magnesium is short,^43^ and the duration of its clinical effect in patients with asthma is unknown. This uncertainty may be associated with reluctance to discharge patients home without further clinical and safety data. Third, although ED throughput benchmarking requires efficient patient disposition decisions, the need for continued observation after administration of IV magnesium before discharge home may also contribute to the propensity of near-routine hospitalization after IV magnesium therapy. Fourth, the minimal change in PRAM scores between 60 minutes after randomization and the time of ED disposition in children administered IV magnesium suggests a poor clinical response to the overall ED therapy, which may have been associated with hospital admission decisions. In addition, the unknown safety associated with discharging a patient home after administration of IV magnesium is a likely contributor to the aforementioned practice pattern.^40^ Of importance, the association between IV magnesium therapy and hospitalization decreased over time. Although the reasons for this change remain uncertain, the trend may reflect increasing comfort of the ED physicians with discharging patients home after IV magnesium therapy.

A key systematic review by Griffiths and Kew^38^ of IV magnesium–associated outcomes of acute pediatric asthma showed a possible reduction in hospitalizations after IV magnesium administration and also indicated the limitations of the available evidence. Specifically, the systematic review highlighted that only 3 small single-center pediatric studies focused on hospitalization after IV magnesium therapy.^10,11,12^ These studies reached disparate conclusions, with heterogeneity in patient populations, study entry criteria, and initial stabilization regimens.^10,11,12^ A reanalysis of the data using a more robust random-effects method determined that IV magnesium was not associated with hospitalizations.^38^ Moreover, safety-related data such as revisits after discharge were not evaluated. The 2 studies that reported a positive association of IV magnesium therapy with hospitalization excluded preschoolers,^10,11^ who account for most ED visits for pediatric acute asthma.^44^ For these reasons, Griffiths and Kew^38^ expressed a low level of confidence in the available evidence. A similar cautionary conclusion was reached in a more recent review.^40^ Therefore, more definitive evidence is needed to inform clinical care about the effectiveness of IV magnesium therapy for acute pediatric asthma.^38^

Preliminary evidence suggests that discharge of select children with asthma after administration of IV magnesium may represent a reasonable management option. In a Pediatric Emergency Care Applied Research Network retrospective multicenter registry review^15^ of 61 854 ED visits, the revisit rates among children discharged from EDs after administration of IV magnesium did not differ from those among children who did not receive this medication. Similarly, in the current study, none of the children who received IV magnesium and were discharged home returned for medical care, further supporting the safety of IV magnesium therapy while simultaneously highlighting the importance of further research on this outcome. Of interest, almost all children in this study who were discharged home after IV magnesium therapy were managed at 1 center, which had the highest rate of IV magnesium use and therefore possibly greater confidence in its use.

### Limitations

This study has limitations. First, IV magnesium was evaluated in the context of an RCT of nebulized magnesium. Lack of adequate response to the experimental therapy may have prompted some physicians to consider the need for IV magnesium and hospital admission, with the use of IV magnesium as a third-line option. Second, because we did not ask physicians about their reasons for and goals of IV magnesium therapy, we cannot be certain of our postulate that IV magnesium is rarely used to prevent hospitalization. However, the study’s findings are consistent with this reasoning. Third, the association between IV magnesium therapy and hospitalization does not imply a cause and effect relationship. Fourth, the PRAM measurement at disposition reflects the clinical status after the administration of the entire treatment regimen given in the ED, including nebulized magnesium or placebo, and not the clinical status after IV magnesium therapy alone. Fifth, residual confounding represents a limitation because there may have been differences in unmeasured variables between patients given IV magnesium and those not given this treatment. Also, some potential confounders, such as specific viral etiologic pathogens, were not assessed. Sixth, the exact time of the hospitalization decision was unknown. As such, hospitalization may not always reflect asthma severity after IV magnesium therapy; many children may have been hospitalized owing to their severe asthma status at arrival to the ED and lack of improvement after inhaled magnesium therapy rather than owing to lack of response to IV magnesium therapy. Therefore, use of IV magnesium therapy may have reflected persistent lack of improvement and may have also triggered the hospitalization decision despite eventual mild asthma status. In addition, because children treated for asthma in community EDs are less likely to receive IV magnesium therapy^15^ and more likely to be hospitalized compared with children treated in pediatric EDs,^33^ this study’s results may not be generalizable to such settings.

## Conclusions

In this secondary analysis of an RCT, we found that the administration of IV magnesium therapy among children with refractory acute asthma was associated with hospitalization independent of asthma severity and other patient-level characteristics. Furthermore, after adjustment for patient-level characteristics, this association was also evident among children who met criteria for hospital discharge at the time of ED disposition. Future research on the benefit of IV magnesium therapy in reducing hospitalization and the related safety profile may clarify the role of this therapy in refractory pediatric asthma.

## Notes

Supplement 1. Trial Protocol

Supplement 2. **Nonauthor Collaborators.** Pediatric Emergency Research Canada
