# Supplementary material for: Association Between Intravenous Magnesium Therapy in the Emergency Department and Subsequent Hospitalization Among Pediatric Patients With Refractory Acute Asthma: Secondary Analysis of a Randomized Clinical Trial
Source: JAMA Netw Open. 2021 Jul 19;4(7):e2117542. doi: 10.1001/jamanetworkopen.2021.17542 (PMC8290299; doi:10.1001/jamanetworkopen.2021.17542)
Supplement: Supplement 1. — Trial Protocol [file jamanetwopen-e2117542-s001.pdf]

Inhaled Magnesium in Refractory Pediatric Acute Asthma

Principal Investigator:

Dr. Suzanne Schuh: The Hospital for Sick Children, Toronto, Ontario

Co-Investigators:

Dr. David Johnson: Alberta Children's Hospital, Calgary, Alberta

Dr. Roger Zemek: Children's Hospital of Eastern Ontario (CHEO), Ottawa, Ontario

Dr. Stephen Freedman: The Hospital for Sick Children, Toronto, Ontario

Dr. Chantal Guimont: Unité de médecine familial, Pavillon CHUL, Université Laval, Quebec

Dr. Darcy Beer: Winnipeg Children's Hospital, Winnipeg, Manitoba

Dr. Amy Plint: Children's Hospital of Eastern Ontario (CHEO), Ottawa, Ontario

Dr. Karen Black: IWK Health Centre, Halifax, Nova Scotia

Dr. Andrew Willan: The Hospital for Sick Children, Toronto, Ontario

Dr. Francine Ducharme: CHU – Sainte-Justine Pédiatrie, Montreal, Quebec

Dr. Graham Thompson: Alberta Children's Hospital, Calgary, Alberta

Dr. Allan Coates: The Hospital for Sick Children, Toronto, Ontario

Background and Significance

Acute asthma is a leading cause of pediatric emergency visits and hospitalizations.[1] In 2005, there were 754,000 pediatric ED asthma visits in the US[2-3], 15-20% of these require hospitalization and another 10-20% relapse within two weeks.[4] However, a 2006 asthma audit of a Canadian pediatric ED shows that 30% of children remaining in moderate and severe distress following initial stabilization therapy are hospitalized and that this population represents 84% of all children admitted to hospital with acute asthma.

Standard therapy of acute severe asthma consists of frequent inhaled $ß_2$ agonists, anticholinergics and corticosteroids.[5-15] However, this regimen has a high degree of outcome heterogeneity since the full benefit of corticosteroids is often not apparent until well beyond the purported 4 hour interval[16] and a substantial proportion of children responds poorly to $ß_2$ agonists (this resistance is in part determined by $ß_2$ adrenoreceptor gene polymorphism).[16-34] Current stabilization therapy[5, 14-15] is not always effective in severe attacks[35] and related costs remain high.[36] *Since these insufficient/poor responders represent virtually all pediatric asthma hospitalizations[37] and since hospitalizations account for 43% of the pediatric asthma care costs[38], finding effective strategies to decrease their morbidity is imperative.* Two adjunctive interventions poorly explored in the acute care setting are not ideal for the ED – IV methylxanthines are associated with significant toxicity and no longer recommended[39-40] and IV $ß_2$ agonists are generally reserved for ICU.[41-43]

Mg is a powerful relaxant of airway smooth muscle[44], with a rapid effect when given IV. It relieves bronchoconstriction by decreasing the uptake and release of calcium in bronchial smooth muscle[45], inhibiting release of acetylcholine[46] and of histamine release and stimulating nitric oxide and prostaglandin synthesis.[46] Furthermore, Mg augments the effect of $ß_2$ agonists by upregulating $ß_2$ receptors[47] and also reduces neutrophilic burst seen with the inflammatory response.[48] Mg can be given either IV or by nebulization. Two key meta-analyses confirm that the addition of IV Mg to routine therapy significantly improves hospitalizations and lung function.[49-50]The authors and several major asthma guidelines recommend that IV Mg be considered in children not responding to initial management.[49, 51-53] However, our survey "North American Practice Patterns of IV Mg in Severe Acute Asthma in Children" showed that 24% of participants have personally witnessed an Mg-attributed hypotension requiring treatment which, along with the belief that most children with asthma improve without an IV constitute major barriers to the use of IV Mg.[54] These results suggest that adverse effects of IV Mg may not be rare. Furthermore, IV access is much more difficult in young children (who make up the majority of children with asthma) than in adults, and multiple attempts are often required which can lead to an increasing cycle of crying and severe respiratory distress.[55-60] Other theoretical adverse effects after IV Mg administration include apnea and heart block.[61] However, none of the IV or inhaled Mg trials has reported either of these complications.

In contrast, the nebulization route is non-invasive and offers a major advantage of targeted delivery to the lower airway and less potential for side-effects,[62] due to a lower systemic delivery of Mg (1/4 of the IV dose). With IV delivery of Mg, the greatest tissue exposure within the lung is in the alveoli and Mg has to diffuse from the thicker-walled pulmonary and bronchial circulation to reach the smooth muscles of the airways. In contrast, most inhaled Mg would be deposited in the airways and direct diffusion through airway epithelium would result in much higher Mg levels around the smooth muscle as compared with IV delivery. However, the investigation of the efficacy of nebulized Mg has been sparse and has yielded disparate results. Seven studies have compared the benefit of adding nebulized Mg to salbutamol to salbutamol alone[63-69]; only one was limited to children.[64] Almost all studies included

asthmatics with negligible admission rates and only one study[63] limited participants to non-responders to bronchodilators who are most likely to benefit from nebulized Mg. This key study by Hughes et al (52 adults) showed a 30% risk reduction in hospitalizations favoring Mg (71% in controls and 43% in the Mg arm).[63] One small study of 62 school-aged children with acute asthma[64] found that a single dose of nebulized Mg added to salbutamol and systemic corticosteroids was associated with a significant improvement in FEV_1 compared to standard therapy at 10 minutes. However, ipratropium was not used, only one patient in each group was hospitalized and the authors did not examine the impact of Mg on other patient outcomes. A Cochrane systematic review by Blitz[70-71]evaluated 6 trials, 4 of which compared nebulized Mg with $ß_2$ agonists to $ß_2$ agonists alone.[63-64, 68-69] There was a clear additive benefit of Mg and salbutamol on lung function in adults with severe disease and a trend towards benefit with respect to lung function and hospitalizations in moderate asthma. A later systematic review[49] of 7 studies[63-69] found an overall treatment effect of Mg and $ß_2$ agonists on both the respiratory function and hospitalization rate approaching statistical significance (p values 0.08 and 0.06, respectively). ***Notably, none of the studies to date have incorporated ancillary evidence-based initial treatments known to reduce hospitalizations such as ipratropium bromide[70]. Also, there was lack of emphasis on patients at high risk of hospitalization.*** Furthermore, the delivery systems used were poorly described and were of low efficiency. Given the encouraging preliminary evidence of benefit, the non-invasiveness and high safety likelihood of the nebulization route and the expertise of our team to ensure Mg delivery, a pediatric study is needed to define the role of nebulized Mg.[49, 70] Addition of nebulized Mg should decrease hospitalizations in asthmatic children remaining in moderate to severe distress after optimized baseline treatment which would immediately impact current clinical practice and decrease morbidity of this high-risk population

Acute asthma is the most common cause of pediatric hospitalizations. While we know that repeat inhalations of $ß_2$ agonists and ipratropium with early oral steroids substantially reduce hospitalizations, many children are resistant to this standard initial therapy. About a third of children remaining in moderate to severe distress after standard therapy are admitted to hospital and comprise 84% of pediatric acute asthma hospitalizations. ***Finding safe, non-invasive, and effective strategies to treat children resistant to standard therapy would substantially decrease hospitalizations resulting in considerable health care savings and reduction of the psycho-social burden of the disease.*** While studies of magnesium sulfate (Mg) given intravenously (IV) suggest that this agent can reduce hospitalizations in both adults and children resistant to standard initial therapy, a North America-wide survey completed by us shows that only 7% of Emergency Department (ED) physicians give IV Mg to prevent hospitalizations, less than 5% of children given IV Mg go home from the ED, and IV Mg is primarily used by physicians to prevent admissions to the ICU. Barriers to IV Mg use include concern about side effects, with 24% of physicians reporting having observed IV Mg-related hypotension requiring treatment as well as a belief that IV therapy is unnecessary. Nebulization is an alternate route for administering Mg. This route has the advantage of being non-invasive and is likely much safer due to lower systemic delivery. Direct delivery via nebulization allows higher Mg concentrations at the target site, the lower airways, with a smaller total drug dose. Two meta-analyses of studies of nebulized Mg – all but one of which have focused on adults - have found that its effect on hospitalizations approaches statistical significance (p=0.08). As a result, the authors of these meta-analyses have ***called for a properly designed study to clarify the role of nebulized Mg. We propose to conduct such a trial.***

We plan the following specific aims:

1. **<u>Primary Objective:</u>** To examine if in children with acute asthma remaining in moderate to severe respiratory distress despite maximized initial bronchodilator and steroid therapy there is a reduction in hospitalization rate from the ED in those who receive nebulized Mg with salbutamol versus those receiving salbutamol only.
   <u>Hypothesis</u>: We hypothesize that the children with Pediatric Respiratory Assessment Measure (PRAM) ≥ 5 points after optimized initial inhaled bronchodilator and oral steroid therapies who are given nebulized Mg in addition to nebulized salbutamol will have significantly lower hospitalization rate within 24 hours of starting the study compared to those given salbutamol only.
2. To compare a difference in the changes in the validated Pediatric Respiratory Assessment Measure (PRAM), respiratory rate, oxygen saturation and blood pressure from randomization baseline to 240 minutes in the two groups
3. To determine if there is a significant association between the difference in the primary outcome between the groups and the patient's age, gender, baseline PRAM score, personal history of atopy and "viral-induced wheeze" phenotype.

Hypothesis(es) to be Tested
In this randomized, double-blind two-centre trial, we hypothesize that children with acute asthma with a Pediatric Respiratory Assessment Measure (PRAM) of ≥ 5 points after optimized initial inhaled bronchodilator and oral steroid therapies who are given nebulized Mg in addition to nebulized salbutamol will have at least a 15% lower hospitalization rate within 24 hours of starting the study as compared to those given salbutamol only.

Supportive Preliminary Data

A. North American Practice Patterns of IV Magnesium in Severe Acute Asthma in Children (NAPP SAAC Survey)[54]

We have recently carried out a continent-wide survey of the Pediatric Emergency Research Canada network and of Pediatric Emergency Medicine- Collaborative Research Committee consortium (US) with the main objective of investigating the frequency of use of IV Mg in stable and critically ill children with severe acute asthma, usual therapeutic goals with respect to disposition and factors impacting the use of this intervention.[54]

Summary of results:

Response rate to the survey: 70% in Canada and in the United States, results almost identical on both sides of the border

Majority of physicians use IV Mg in less than 20% of children with stable severe acute asthma

Only 7% of the ED physicians give IV Mg to prevent hospitalizations

71% give IV Mg to prevent ICU admission

Less than 5% of children given IV Mg in the ED are discharged home from the ED

24% of the ED physicians have personally witnessed IV Mg - related hypotension requiring therapy

Notable barriers to the use of IV Mg: a) concern about side effects and b) desire to avoid an IV

97.0 % of physicians felt that if high quality evidence of benefit of nebulized Mg were available, they would incorporate it into their practice.

These results show that serious adverse effects associated with IV Mg are not rare and that significant valid barriers to its use do exist. Investigation of the benefit of Mg given via an alternate route such as nebulization is therefore in order.

B. Selecting an optimal nebulization system for delivering inhaled magnesium[72]:

An important limitation of previous studies was the use of low-efficiency nebulizers which may have accounted for the disparity in the results. We have therefore conducted ***a pilot study to investigate expected lung deposition of Mg using three modern nebulizers:*** **the Pockethaler®/Aeroneb Pro** system (La Diffusion Technique, St. Etienne, France) with a holding chamber connected to a vibrating membrane nebulizer, **the Omron** vibrating membrane system (Kyoto, Japan) and the breath-enhanced **Pari Star** nebulizer (Munich, Germany). We used a breath simulator using a breathing pattern of 15 breaths per minute, tidal volume of 0.6 L and inspiratory to total respiratory cycle time of 0.4.[73-74] The three nebulizers were charged with an identical solution to that to be used in proposed trial: 600 mg [500 mg/ml] of Mg sulfate (Sandoz), 5 mg [5 mg/ml] of salbutamol (GSK) and 3.5 mL sterile water. *This Mg dose is at the upper end of the range of nebulized Mg doses used in previous trials*[65] [range of total Mg dose per study: 225-1500 mg] *and minimizes the possibility of under treatment in case no benefit is found.*

An inspiratory filter was placed at the output of the breath simulator. Mg collected from the filter represented the amount of Mg expected to enter the airway. The fraction of the aerosol expected to deposit in the lungs is the respirable fraction which depends on a high proportion of aerosol particles ≤ 5 µm in diameter.[75-76] We measured the particle size of the aerosol by laser diffraction (Malvern Instruments, Worchestershire, UK).[77] The pulmonary deposition of Mg in mg/min can be estimated by multiplying the amount of aerosol captured on the filter (expressed as mg of Mg collected per minute) by the respirable fraction.

The Pockethaler® system produced an estimated pulmonary Mg deposition of 12 mg/min (or 240 mg during a 20 minute nebulization) and a deposition of 61 mcg/min of salbutamol. These salbutamol deposition data are highly comparable to those previously obtained with a Hudson Updraft nebulizer testing 5 mg of salbutamol which resulted in virtually identical deposition of 60 µg/min.[78] Importantly, 62% of the inhaled particles were ≤ 5 µm in diameter. Furthermore, the osmolarity of the solution at the end of the nebulization was well below 500 mosm/L, a level previously associated with bronchospasm.[79] In contrast, the Pari LC Star yielded a lower Mg deposition of 7.9 mg/min and the osmolarity of the solution at the end of nebulization exceeded 500 mosm/L. Likewise, the estimated deposition of Omron was low (4.9 mg/min).

During the IV administration of Mg using a standard infusion rate of 40 mg/kg IV Mg[80] in a 25 kg child for 20 minutes, the systemic rate of Mg delivery (into the blood) would be 50 mg/min. In contrast, *the data above show that systemic delivery of nebulized Mg via the Pockethaler® nebulizer would be only about 25% of the Mg given during the IV administration which would result in lower circulating blood levels and a lower potential for side effects.* With aerosol delivery being lower compared to IV administration, another two inhalations could be undertaken with little concern about hypotension. *Therefore, the Pockethaler® system was the delivery of choice since it maximizes delivery of Mg to the airways while maintaining safety by minimizing possibility of hypotension from systemic absorption and by maintaining acceptable osmolarity to avoid bronchospasm.*

C. Development and Evaluation of PRAM[37, 81]:

The vast majority of children with acute asthma are of pre-school age and lack coordination to perform pulmonary function tests reliably. Dr Ducharme and colleagues therefore developed and validated the Pediatric Respiratory Assessment Measure (PRAM) as a measure of severity of airway obstruction in acute asthma and its responsiveness to treatment and later evaluated its performance characteristics in children 2 years of age and older presenting with acute asthma in the Emergency Department setting. *This background work will provide us with the ability to use this excellent measurement tool in this trial – both as an entry-severity criterion and as a secondary outcome.*

Experimental Design and Methodology

Primary question: In children 2-17 years of age with acute asthma who have persistent moderate to severe airway obstruction despite maximized initial bronchodilator and steroid therapy, is there a significant difference in the hospitalization rate in those who receive three nebulized Mg and salbutamol treatments compared to those receiving only nebulized salbutamol?

Secondary questions: Between these treatment modalities:

a) Is there a difference in the changes in the validated Pediatric Respiratory Assessment Measure (PRAM), respiratory rate, oxygen saturation and blood pressure from randomization baseline to 240 minutes?
b) Is there a difference in the number of salbutamol treatments within 240 minutes?
c) Does the treatment effect with respect to primary outcome vary between subgroups defined by these variables: age, gender, pre-randomization PRAM score, personal history of atopy and "viral-induced wheeze" phenotype?

Hypothesis: We hypothesize that the children with PRAM ≥ 5 points after optimized initial inhaled bronchodilator and oral steroid therapy who are given nebulized Mg in addition to nebulized salbutamol will have a significantly lower hospitalization rate at the index visit compared to those given salbutamol only.

Trial Design: This is a two-centre randomized double-blind placebo controlled trial. Two study groups will be compared: nebulized salbutamol with Mg sulfate and nebulized salbutamol with placebo.

Triage RN: Baseline PRAM score (**see Appendix A**) in all asthmatics

Moderate to severe disease (PRAM 5-12): assessment by ED physician
Systemic corticosteroids
3 treatments with salbutamol & ipratropium over 1 hour

↓

Study Nurse repeats PRAM at approximately 1 hour, if ≥ 5: assess eligibility criteria.
(If PRAM is < 5, subject is ineligible for study)

↓

If eligible, obtain informed consent
Baseline measurements (BP, RR, HR, O2 Sat, FEV1 if possible)
Obtain treatment allocation assignment
Retrieve treatment package from ED/pharmacy

↓

Experimental Group Treatment
Mg (600 mg) and Salbutamol (5mg)
@ 0', 20', 40'

Control Group Treatment
Placebo and Salbutamol (5mg)
@ 0', 20', 40'

PRAM, RR, HR, O_2 sat, FEV1 @ 0'(pre experimental Rx), 60' (post experimental Rx), 120', 180', 240'
BP @ 0', 20', 40', 60', 120', 180', 240'
(These study procedures can be done +/- 10 minutes at each time point)

↓

Further bronchodilator therapy as clinically indicated

↓

Disposition decision by ED MD

↓

Electronic/paper health chart review and telephone follow-up at 72 hrs (+4 days)

Inclusion criteria:

(1) **2-17 years** of age

(2) **Diagnosis of asthma**, defined as this diagnosis made by a physician and at least one prior acute episode of wheezing with cough or dyspnea treated with inhaled $ß_2$ agonists or oral corticosteroids. *Our study population will exclude bronchiolitis and first-time wheeze (potential alternate diagnoses).*

(3) ***Persistent* moderate to severe airway obstruction** after 3 doses of salbutamol and ipratropium, defined as a PRAM 5 or higher. *A PRAM score of 5 or more following initial therapy indicates the child has at least moderate disease severity[37] and has a high likelihood of being hospitalized.[37] This group of children includes 84% of all pediatric asthma hospitalizations; therefore, finding an effective therapy for this population has great potential to significantly reduce hospitalizations. (Appendix B).*

Although the inclusion of children with PRAM scores of 4 or more would enable us to capture nearly all asthma admissions, their admission rate is substantially lower (20%) and thus the overall baseline likelihood of admission would be reduced (Appendix B). Although the admission rate for children with PRAM of 6 or more is high, randomizing only this population would miss 30% of asthma hospitalizations (Appendix B). For these reasons, we have chosen to randomize children with PRAM 5 or more after initial bronchodilator therapy.

Although the PRAM scores of most children will improve following the initial treatment, 35% of those with a presenting PRAM of 5 points do not change (Appendix B). Thus, to maximize capturing this high-risk population, we shall screen and perform post-bronchodilator therapy PRAM scores on all previously healthy children in the target age-range with a presenting PRAM of 5 points or more.

Exclusion Criteria:

(1) No previous history of wheezing or bronchodilator therapy. *Some children who present with wheezing for the first time will have other diagnoses which would not be expected to respond to Mg.*

(2) Patients who have already received IV Mg therapy during the index visit.

(3) Critically ill children requiring immediate intubation. *These children need immediate ICU management and hospitalization.*

(4) Children who in the opinion of the treating physician require a chest radiograph due to atypical clinical presentation and are found to have radiologist-confirmed pneumonia. *These rare patients may have to be hospitalized primarily for treatment of the infection and may not respond to magnesium.*

(5) Known co-existent renal, chronic pulmonary, neurologic, cardiac or systemic disease. *These conditions may influence the response to Mg and hospitalization*.

(6) Transfers from other institutions. These patients would have received initial therapy well before arrival, potentially represent a different stage of their acute disease and may respond differently to Mg therapy.

(7) Known hypersensitivity to Mg sulfate.

(8) Patients previously enrolled in the study.

(9) Insufficient command of the English language.

(10) Lack of a home or cellular telephone.

Sample Selection: Children presenting to the EDs at The Hospital for Sick Children and the Alberta Children's Hospital when the research nurses are on duty (days and evenings) who meet the eligibility criteria will be approached for enrollment. The research nurses will keep a log of all children presenting to the ED with acute asthma during the study period whether randomized or not in order to assess the generalizability of the study. Both aforementioned hospitals are tertiary care centers, which see the entire clinical and demographic spectrum of the asthma population. Our profile of children with acute asthma should therefore be comparable to that of other institutions and the generalizability of the study

should not be affected and the referral bias should be minimal. A structured data collection form will be used to assess the baseline and demographic features that may affect outcome and potentially confound the comparisons. Since the patients will be screened consecutively and study coverage will occur during days, evenings and weekends, selection bias should not play a major role.

Randomization: The Research Support Pharmacist at The Hospital for Sick Children will produce Master Randomization tables, stratified by site and age (5 years or less versus more), using a permuted block randomization in a 1:1 ratio of active Mg sulfate to placebo, using random number generating software. The Master Randomization table will be held at the Research Pharmacy at The Hospital for Sick Children, which is open 24 hours a day. The Research Support Pharmacist at The Hospital for Sick Children will also prepare and distribute consecutively numbered kits. Upon obtaining the informed consent, the local study nurse will obtain the next appropriate numbered study kit from a locked research refrigerator and enter the kit number in the confidential study log book.

Blinding: In this study, the patients, families, research nurses and ED physicians will be blinded to the treatment assignment. The SickKids Research Pharmacist will prepare numbered kits with 3 vials containing: a) $MgSO_4$ for active kits or hypertonic 5.5% saline placebo for placebo kits (to match the high tonicity of Mg Sulfate), b) open label salbutamol nebulizer solution and c) sterile water as the top up diluent (sterile water chosen as the top up diluent since mixing normal saline with Mg sulfate is hyperosmotic; using sterile water will keep the osmolarity of both solutions well below the 500 mOsm/L that may be associated with bronchospasm) (See Appendix C). The active Mg and placebo hypertonic saline mixture with salbutamol and sterile water are similar in volume, color, taste and smell when nebulized. The study nurse, physicians and patient will be unaware of the next group assignment. Only the pharmacy at The Hospital for Sick Children will be unblinded. We acknowledge the remote possibility of indirect unblinding because a decrease in blood pressure may rarely occur during Mg therapy. However, major hypotension is unlikely and the likelihood of inadequate blinding is thus very low. To assess blinding, the research nurse and parents/patients will be asked at the conclusion of experimental therapy which intervention they think the child had received.

Pre-Study Screening and Baseline Evaluation: All previously healthy children 2-17 years of age with acute asthma will have a PRAM score measured in triage. Those with a presenting PRAM ≥ 5 will be assessed by the ED physician and receive either oral dexamethasone (0.3mg/kg), oral prednisolone/prednisone (2mg/kg) or IV hydrocortisone (5mg/kg) [all considered equivalent for reducing hospitalizations] plus three salbutamol and ipratropium inhalations standardized by dose via Metered Dose Inhaler/Valved Holding Chamber (MDI/VHC) 20 minutes apart. Children weighing less than 15 kg will receive 400mcg=4puffs of salbutamol and 80mcg=4puffs of ipratropium/dose via MDI. Children ≥ 15 kg will receive 800mcg=8puffs of salbutamol and 80mcg=4puffs of ipratropium/dose via MDI.[78, 82-87] These medications and doses are part of the standard of care. Ipratropium bromide decreases hospitalizations in asthmatic children with evidence of major distress[88], such as marked neck retractions and extensive wheeze, reflected in a PRAM score of 5. *Our baseline initial therapy is therefore optimized and insufficient improvement/persistent respiratory distress justifies further intervention in this population.*

Study Procedures: At approximately 1 hour after the first three inhalations have been given, the research nurse will assess eligibility for the study and measure the pre-randomization PRAM score. Eligible children with PRAM[81] ≥5 points after three bronchodilator treatments [at least moderate to severe respiratory distress] will be approached and informed consent will be obtained. Subjects will be randomly allocated to receive three consecutive nebulizations of salbutamol with either diluted Mg sulfate or diluted hypertonic saline placebo 20 minutes apart (+/- 10 minutes), using the Pockethaler®

system. Since three nebulizations were used in the adult study that demonstrated the greatest benefit of Mg[63], likewise we will use the same number in this study. Specifically, each treatment will utilize 600 mg (1.2 mL) of Mg sulfate (hypertonic)or 1.2 mL hypertonic 5.5% saline (*to match osmolarity of Mg sulfate-see Appendix C for details)*, 5 mg (1 mL) of salbutamol and 3.6 mL of sterile water. *Our Mg dose approximates the upper end of the Mg dosing range used in previous studies, selected to maximize the therapeutic potential of inhaled Mg. Administration of multiple experimental inhalations will have the advantage of better drug distribution in the lungs after the first treatment when some bronchoconstriction will have been relieved.*

In order to minimize the possibility of cough/bronchospasm which can on occasion be seen with inhaling solutions with osmolarities above 500 mOsm/L[79], we plan to employ a solution with an osmolarity well under 500mOsm/L. In order to ensure that any potential differences in side effects/treatment effect were not due to a difference in the osmolarity of the two solutions, we had to ensure that both the active and placebo arms solutions were of comparable and acceptable osmolarities. *Magnesium sulphate injection solution itself is hyper-osmolar. 5.5% saline has the same osmolarity as magnesium sulphate.*

The use of ***sterile water as the top up diluent in both the active Mg/salbutamol arm and the placebo 5.5% saline/salbutamol arm yields a highly acceptable final osmolality of 384 mOsm/L in both study arms*** (Appendix C). Using normal saline as the top up diluent in the active arm would result in a higher osmolality which would exceed the upper limit of acceptability of 500 mOsm/L. Therefore, normal saline cannot be used as the top up diluent.

The use of 5.5% saline as the placebo and of sterile water as the top up diluent in both arms creates comparable experimental conditions in both study arms (Appendix C). We have also pre-tested that the Pockethaler® nebulizer maintains isotonicity of both active and placebo solutions throughout nebulization, thereby minimizing the possibility of side- effects.

Pre-randomization, the study nurse will measure the subject's PRAM score, respiratory rate, heart rate, oxygen saturation, blood pressure and the FEV1 (if possible). The study nurse will measure these parameters at 60 minutes and hourly thereafter up to 240 minutes and blood pressure will also be assessed after each experimental nebulization at 20, 40, 60 minutes. These study procedures can be done +/- 10 minutes at each time point. The study nurse will also record the details of all other pharmacotherapy given as well as disposition status during the index visit. The research nurses will ascertain subsequent return visits/hospitalizations-both from the telephone follow-ups as well as from a review of the patient health records including any records from their family doctor if necessary at 72 hours. At this time the parents will also be questioned about unscheduled medical visits related to asthma and further therapies instituted. If families cannot be reached during mutually agreed upon times at 72 hours, daily phone calls will be made until day 7. If Hospitalized patients will not be contacted by the research nurse for a telephone follow-up.

Following this experimental intervention, participating children will continue to receive further salbutamol treatments as frequently as clinically warranted as per the treating ED physician. Disposition will also be determined by the ED physician, independently of the knowledge of the study intervention. If the patient has improved and the ED physician feels that he/she can go home, the patient can be discharged prior to the 240 minute study assessment. Discharged patients will receive a prescription for 400 mcg = 4puffs of salbutamol/dose via MDI up to every four hours as necessary for the next week in addition to daily oral prednisolone 1mg/kg for 5 days (maximum 60 mg) as per standard of care. All participating families will receive instructions to visit their primary care provider/ED if salbutamol has

to be given more often than every 4 hours for increased work of breathing/severe cough and if the respiratory status interferes with usual play/normal speech or routine activity.

In case of increasing respiratory distress, IV Mg may be given after the experimental therapy, as clinically warranted as per the treating ED physician, provided there are no Mg side effects after the study intervention. In the unlikely event the patient develops hypotension requiring therapy, apnea, heart block or another adverse event and the ED physician feels that the experimental therapy cannot be safely continued, further doses of the experimental treatment will be stopped. If these adverse events are accompanied by severe distress and additional IV Mg is warranted, the study may be unblinded for that subject. If the subject was allocated to the active Mg Sulfate arm, then additional IV Mg should not be given but alternative treatment provided instead. If the subject was allocated to the Placebo arm, then IV Mg may be given as part of treatment of the adverse event. Unblinding should only be requested when the clinical treatment of the patient will be different by knowing which arm of the study the patient was previously on. The study PI and the study nurses will remain blinded. (Appendix D)

The primary outcome measure will be hospitalization defined as admission to an inpatient unit within 24 hours of the start of the experimental therapy due to continued/worsening respiratory distress. Those children in whom a decision to admit was made by the treating physician, but due to lack of bed availability were never transferred to the inpatient unit will be analyzed as admitted as will those returning to the ED within 24 hours of the start of the study who require hospitalization for asthma. It is extremely unlikely that admissions would occur primarily for reasons other than respiratory distress. The study nurse will ascertain that the hospitalizations are for respiratory distress versus other reasons. Should the latter scenario occur, these children will be identified and not counted as hospitalized. If the nurse leaves before disposition has been finalized he/she will review the ED electronic data records to identify the length of stay, final disposition, number of bronchodilator treatments by this time and other outcomes the next day. He/she will also communicate with the treating ED physician regarding the reason for hospitalization.

Hospitalization is a powerful marker of treatment failure, a decrease in which is likely to impact practice and influence decision makers since almost a half of pediatric asthma costs, relate to hospitalizations.[89] Hospital admission can also be a very stressful even for both the caregivers and patients. It impacts on the rest of the family since caregivers have to take time off work and arrange alternative sources of care for the other children.

Secondary outcome measures - The two groups will also be compared with respect to:

a. Changes in the PRAM, respiratory rate and oxygen saturation from the start of the first experimental nebulization to 60, 120, 180 and 240 minutes and the changes in the blood pressure from the first experimental nebulization to 20, 40, 60, 120, 180 and 240 minutes.
b. Number of salbutamol treatments within 240 minutes of starting experimental therapy.
c. An association between hospitalization and age, gender, pre-randomization PRAM score, personal history of atopy, and “acute viral induced wheeze” phenotype.[90] This phenotype will be defined by age less than 5 years, co-existent upper respiratory tract infection, no interval symptoms between exacerbations, no atopy.[90-96]

Other outcomes

Unscheduled visits for asthma to any medical facility within 72 hours of the start of the study. *Most return visits for acute asthma occur within this period.* However, this will be an uncommon event and a meaningful analysis may not be possible.

Major side-effects such as hypotension (systolic blood pressure below 5th percentile for age) or apnea will be tracked as will be admission to ICU for airway stabilization. These outcomes are extremely rare (unstable children will be excluded) and the study cannot therefore be powered for their meaningful statistical analysis. *However, these data are critical to estimate a safety profile of inhaled Mg in children.* We shall measure the Forced Expiratory Volume in one second (FEV1) at baseline as well as at the other aforementioned times in children 6 years old and older.[97] However, we may not be able to analyze the results as most study patients will be pre-schoolers who cannot perform the necessary maneuvers reliably. Moreover, more than two thirds of the older children with severe asthma enrolled in our previous studies were unable to perform reliable lung function measurements.

PRAM is a validated 12 point clinical asthma severity score[81] exhibiting the most comprehensive measurement properties of all asthma scores[98] which has been successfully used as an outcome in major trials.[99] *It is the only score with demonstrated criterion validity, using respiratory resistance as the gold standard.*[100] This instrument has recently been validated in both preschool and school aged acute asthmatics in the ED *and has strong association with admission, thus supporting its ability to distinguish across severity levels.*[37] *The score has inter-rater reliability consistently above 70%* [37]*and is currently implemented in numerous pediatric EDs across Canada.* In contrast, the Pediatric Asthma Severity Score[101] has not been validated against a concurrent measure of lung function and may not be as responsive as the PRAM due to a smaller range. The vast majority of children treated for acute asthma are preschoolers[102] who lack sufficient coordination to perform pulmonary function tests reliably. Both The Hospital for Sick Children and the Alberta Children's Hospital now measure the PRAM score as part of routine clinical assessment in their EDs in children with acute asthma.

Study Implementation: Prior to the study, the ED staff physicians and fellows and emergency nurses will be educated in all aspects of the study. Particular attention will be paid to the importance of communicating to the research nurse the reasons for hospitalization and the importance of protocolized stabilization therapy. The research nurses will be trained in all aspects of the study execution, including obtaining informed consent, technical aspects of administering nebulized treatments and the PRAM measurement.

This study requires the following personnel:

1. Study coordinator at The Hospital for Sick Children who will communicate with the PI, the co-PI and the study nurses regarding starting the study at both sites, data transfer, study-related enrollment and logistic issues, facilitate the REB-related matters as well as oversee the budget and organize the study log in Toronto.
2. Two research nurses or respiratory therapists in Toronto and one in Calgary will be responsible for screening, enrollment and study execution and use of the data collection forms.

Sample Size: A 2006 prospective audit of 1000 children presenting with acute asthma at a Canadian ED showed that approximately 30% of patients with a PRAM score of 5 or greater after bronchodilator therapy were hospitalized (Appendix B). The sample size calculation is based on the assessment of the between-group difference in proportions of hospitalizations. This is a superiority study in which the adoption of the Mg therapy can only be recommended for future practice if the rate of the primary outcome in this group is significantly lower than in the controls. The null hypothesis for the primary analysis is that the probability of hospitalization is the same in both arms. The specific alternative hypothesis for which we wish to have sufficient power is that the hospitalization rate in the Mg arm is lower by at least 15 percentage points (absolute difference). A discussion among investigators revealed that a difference of this magnitude would warrant adoption of nebulized Mg. This target difference is also chosen since it would have significant economic impact given the high frequency of acute asthma.

For a two-sided test to have a type I error rate of 5% and a power of 80%, we need to randomize 142 patients per arm, for a total of 284.[103] (A nominal value of 4% was used for the type I error rate to account for the interim analysis.) To be conservative, we assume a refusal rate of 40% and a loss to follow-up of 5%. Therefore, to have complete data on 284 patients, we plan to randomize 300 (*i.e.* 284/(1 - 0.05) and to approach 500 (*i.e.* 300/(1 - 0.4) for randomization.

Statistical Analysis:

The primary analysis:

A two-sided Fisher's exact test will be used to test the null hypothesis that the treatment arms are equal with respect to the probability of hospitalization. This analysis will be performed on all randomized patients, according to the intent-to-treat principle, using a two-sided test of hypothesis with a type I error of 0.05. A nominal level of 4% for the type I error rate will be used to account for the interim analysis.

The secondary analyses:

a) Repeated measures ANOVA to compare treatment arms with respect to the changes in the PRAM score, respiratory rate, heart rate, oxygen saturation, and blood pressure over time.
b) A Poisson model will be used to compare the number of salbutamol treatments used in the ED in the two study arms.
c) Logistic regression analysis, including interaction terms with treatment group, will be used to examine the subgroup effects with respect to the primary outcome. The following variables will be used to define subgroups: age, gender, pre-randomization PRAM score, personal history of atopy.
The statistical tests of hypotheses for the secondary outcomes a) through c will two-sided at the 0.017 level to account for the issue of multiple testing and to maintain an overall type 1 error rate of 0.05.

Interim Analysis: To ensure safety of the participating subjects, there will be one planned interim analysis on the first 142 patients randomized. The interim analysis will be a two-sided test of the null hypothesis that the treatment arms are equal with respect to the probability of hospitalization. A Fisher's exact test will be used. The interim analysis will use a type I error rate of 1%, and because the final analysis will have a type I error rate of 4%, there will be an overall type I error rate of 5%.

Feasibility: As part of our pilot work, we have obtained information from the medical records from both participating sites. We have found that the ED at the Hospital for Sick Children sees on average 2,382 children two years of age and older with acute recurrent wheeze annually. Of these, 250 are hospitalized and 2,132 are discharged. According to a 2006 asthma audit from a Canadian pediatric ED, 84% of admitted patients and 30% of discharged patients have PRAM scores of 5 or higher after initial bronchodilator therapy. Projecting these percentages to the data above, we would expect 850 children annually to have a PRAM of 5 or higher, of which 250 would be hospitalized.

At the Toronto site, we plan to employ two full-time trained research nurses/respiratory therapists, who will be cover the study 6 days a week and will work *on average* 12 hours a day. 364 patients with a PRAM score ≥ 5 after 3 treatments can be expected to present during this coverage period (Appendix E). Presuming the "worst-case scenario", 10% of these may be missed while the nurses are on duty, 30% can be expected to be excluded for enrollment criteria, 40% may refuse participation and 5% may be lost to follow-up. These estimates are based on 15 respiratory RCTs by the PI and they are highly conservative since our miss rate in comparable studies has consistently been below 5% and loss to follow-up after significantly longer intervals has been less than 2%. Therefore, we would expect to enroll and complete follow-up on 131 analyzable children annually. Since *virtually all asthma cases occur between September and May, these totals represent one 9 month "asthma season"*. To save

money, enrollment will be limited to these periods. With this arrangement, we anticipate to acquire a full sample size by the end of the second 9 month asthma season (Appendix E).

Calgary site: The ED at the Alberta Children's Hospital sees approximately 2000 children two years of age and older with acute recurrent wheeze annually. Of these, approximately 200 are hospitalized and 1800 are discharged. According to the aforementioned evidence, we would expect 710 children annually to have a PRAM of 5 or higher, of which 200 would be hospitalized.

At the Calgary site, we plan to employ one full-time trained research nurse/respiratory therapist, who will cover the study 37.5 hours a week. He/she will work 3 days a week, 12 hours a day *on average* (the study period is quite long, so longer shifts may be more useful). Approximately 152 patients with a PRAM score $\geq$ 5 after 3 treatments can be expected to present during this coverage period (Appendix E1). Presuming the "worst-case scenario", 10% of these may be missed while the nurses are on duty, 30% can be expected to be excluded for enrollment criteria, 40% may refuse participation and 5% may be lost to follow-up. Therefore, we would expect to enroll and complete follow-up on 54 analyzable children in one asthma season.

With this arrangement, we anticipate to acquire a sample size of 316 analyzable children by the end of the second 9 month asthma season from both sites (Appendices E & E1).

Compliance with the experimental therapy is expected to be excellent since the nurses will administer and supervise its delivery in all children and the entire intervention will take place in the ED. They will also ensure the nebulizer mask stays on the face throughout treatment. We have conducted numerous past studies with successful nebulized bronchodilator delivery with a mask-face seal facilitated by the research nurse.[104-115] The experimental period is very short which will also enhance compliance. In our extensive experience, virtually no patients fail to finish experimental therapy. We have adjusted the sample size by 5% to account for /loss to follow-up.

<u>Adverse Events</u>

All adverse events will be reported to the Hospital for Sick Children Research Ethics Board according to the Hospital for Sick Children's adverse event reporting requirements. All serious, unexpected adverse drug reactions to the study medication will be reported to Health Canada within 15 calendar days or for death or life-threatening events, within 7 calendar days. In the latter case, a follow-up report must be filed within 8 calendar days. Adverse reactions will be managed according to the Hospital for Sick Children's standard clinical management practices.

Since hypotension is the only major side-effect of IV Mg occurring with appreciable frequency, all enrolled patients will be on precautionary frequent blood pressure monitoring as per the study protocol. If the systolic blood pressure drops below 5th percentile for age, the study will be stopped, treatment given as necessary and DSMC will be notified.

Due to the osmolarity of the study solutions being well under 500 mOsm/L throughout nebulization and co-administration of salbutamol, we do not anticipate side effects to occur as a result of using the aforementioned composition of the study solutions. However, should the highly unlikely event of respiratory deterioration occur, the experimental therapy will be discontinued, appropriate additional treatment started and the event will be reported to the DSMC within 48 hours.

To ensure safety of the participating subjects, unstable children requiring immediate airway stabilization will be excluded. We are also planning an interim analysis to maximize safety.

Data Safety and Monitoring Committee (DSMC):

The Data Safety and Monitoring Committee (DSMC) will consist of a non-study biostatistician, an ED physician and researcher and an ED scientist. The members of this committee will not be collaborators of this trial. They will be notified of all serious adverse events (such as hypotension <5th percentile for age, apnea, heart block, severe increase in respiratory distress necessitating discontinuation of the study) and of an admission to the ICU within 48 hours. Should any of these adverse events occur, they will be immediately reported from both sites to the study coordinator at SickKids who will promptly notify the DSMC. The DSMC will meet once per asthma season or ad hoc if necessary.

Dissemination of Results and Future Directions: The results of this study will be submitted for presentation at either the annual meeting of the Pediatric Academic Societies, the Society for Academic Emergency Medicine or the American Academy of Pediatrics. We shall also submit the manuscript for publication in a peer-reviewed scientific journal.

Limitations: In this study, we anticipate a very low rate of magnesium-related side effects such as hypotension. The major reason for this is a limited systemic magnesium delivery, which will be much lower than with the IV therapy. However, the study sample size will not permit us to conduct a meaningful statistical analysis of magnesium-related adverse events since we anticipate an extremely small number of such events, if any.

Literature References

1. Mannino DM, Homa DM, Pertowski CA, Ashizawa A, Nixon LL, Johnson CA, et al. Surveillance for asthma--United States, 1960-1995. *Morbidity & Mortality Weekly Report CDC Surveillance Summaries*. 1998;47(1):1-27.
2. Akinbami L. Asthma prevalence, health care use and mortality: United States, 2003-2005. http://www.cdc.gov/nchs/data/hestat/ashtma03-05/asthma03-05.htm. Published November 2006. Updated September 1, 2009. Accessed October 1, 2009.
3. Moorman JE, Rudd RA, Johnson CA, King M, Minor P, Bailey C, et al. National surveillance for asthma--United States, 1980-2004. *Morbidity & Mortality Weekly Report Surveillance Summaries*. 2007;56(8):1-54.
4. Camargo CA, Investigators M. Acute asthma among children presenting to the emergency department: the Multicenter Asthma Research Collaboration. *Acad Emerg Med*. 1998;5(5):380.
5. Scarfone RJ, Fuchs SM, Nager AL, Shane SA. Controlled trial of oral prednisone in the emergency department treatment of children with acute asthma.[see comment]. *Pediatrics*. 1993;92(4):513-518.
6. Robertson CF, Smith F, Beck R, Levison H. Response to frequent low doses of nebulized salbutamol in acute asthma. *J Pediatr*. 1985;106(4):672-674.
7. Schuh S, Parkin P, Rajan A, Canny G, Healy R, Rieder M, et al. High-versus low-dose, frequently administered, nebulized albuterol in children with severe, acute asthma. *Pediatrics*. 1989;83(4):513-518.
8. Plotnick LH, Ducharme FM. Combined inhaled anticholinergic agents and beta-2-agonists for initial treatment of acute asthma in children.[update in Cochrane Database Syst Rev. 2000;(4):CD000060; PMID: 11034671]. *Cochrane Database of Systematic Reviews*. 2000(2):CD000060.
9. Rowe BH, Edmonds ML, Spooner CH, Camargo CA. Evidence-based treatments for acute asthma. *Respir Care*. 2001;46(12):1380-1390.
10. National Heart, Lung and Blood Institute: Global Initiative for asthma. Global strategy for asthma management and prevention. NHLBI/WHO workshop report. Bethesda, Md: NIH; 2002. Report No.: 02-3659.
11. National Institutes of Health and National Heart, Lung, and Blood Institute. Guidelines for the Diagnosis and Management of Asthma - Update on Selected Topics 2002. Washington, DC: US Dept of Health and Human Services; 2002. Report No.: 97-4051.
12. National Asthma Council. *Asthma Management Handbook 2002*. Melbourne: National Asthma Council Australia, Ltd; 2002.
13. British Thoracic S, Scottish Intercollegiate Guidelines N. British guideline on the management of asthma. *Thorax*. 2003;58 Suppl 1:i1-94.
14. Becker A, Lemiere C, Berube D, Boulet LP, Ducharme FM, FitzGerald M, et al. Summary of recommendations from the Canadian Asthma Consensus guidelines, 2003. *CMAJ Canadian Medical Association Journal*. 2005;173(6 Suppl):S3-11.
15. *National Asthma Education and Prevention Program. Expert Panel Report 3: guidelines for the diagnosis and management of asthma.* Bethesda, MD: National Institutes of Health, National Heart, Lung, and Blood Institute; 2007.
16. Hall IP. Pharmacogenetics of asthma. *Eur Respir J*. 2000;15(3):449-451.
17. Drazen JM, Silverman EK, Lee TH. Heterogeneity of therapeutic responses in asthma. *Br Med Bull*. 2000;56(4):1054-1070.
18. Palmer LJ, Silverman ES, Weiss ST, Drazen JM. Pharmacogenetics of asthma. *Am J Respir Crit Care Med*. 2002;165(7):861-866.

19. Koga T, Kamimura T, Oshita Y, Narita Y, Mukaino T, Nishimura M, et al. Determinants of bronchodilator responsiveness in patients with controlled asthma. *J Asthma*. 2006;43(1):71-74.
20. Tsai HJ, Shaikh N, Kho JY, Battle N, Naqvi M, Navarro D, et al. Beta 2-adrenergic receptor polymorphisms: pharmacogenetic response to bronchodilator among African American asthmatics. *Hum Genet*. 2006;119(5):547-557.
21. Johnson M. The beta-adrenoceptor. *Am J Respir Crit Care Med*. 1998;158(5 Pt 3):S146-153.
22. Taylor DR, Epton MJ, Kennedy MA, Smith AD, Iles S, Miller AL, et al. Bronchodilator response in relation to beta2-adrenoceptor haplotype in patients with asthma. *Am J Respir Crit Care Med*. 2005;172(6):700-703.
23. Lipworth BJ, Hall IP, Tan S, Aziz I, Coutie W. Effects of genetic polymorphism on ex vivo and in vivo function of beta2-adrenoceptors in asthmatic patients. *Chest*. 1999;115(2):324-328.
24. Israel E, Drazen JM, Liggett SB, Boushey HA, Cherniack RM, Chinchilli VM, et al. The effect of polymorphisms of the beta(2)-adrenergic receptor on the response to regular use of albuterol in asthma. *Am J Respir Crit Care Med*. 2000;162(1):75-80.
25. Taylor DR, Drazen JM, Herbison GP, Yandava CN, Hancox RJ, Town GI. Asthma exacerbations during long term beta agonist use: influence of beta(2) adrenoceptor polymorphism. *Thorax*. 2000;55(9):762-767.
26. Israel E, Chinchilli VM, Ford JG, Boushey HA, Cherniack R, Craig TJ, et al. Use of regularly scheduled albuterol treatment in asthma: genotype-stratified, randomised, placebo-controlled cross-over trial.[see comment]. *Lancet*. 2004;364(9444):1505-1512.
27. Palmer CN, Lipworth BJ, Lee S, Ismail T, Macgregor DF, Mukhopadhyay S. Arginine-16 beta2 adrenoceptor genotype predisposes to exacerbations in young asthmatics taking regular salmeterol.[see comment]. *Thorax*. 2006;61(11):940-944.
28. Contopoulos-Ioannidis DG, Manoli EN, Ioannidis JP. Meta-analysis of the association of beta2-adrenergic receptor polymorphisms with asthma phenotypes.[see comment]. *J Allergy Clin Immunol*. 2005;115(5):963-972.
29. Martinez FD, Graves PE, Baldini M, Solomon S, Erickson R. Association between genetic polymorphisms of the beta2-adrenoceptor and response to albuterol in children with and without a history of wheezing. *J Clin Invest*. 1997;100(12):3184-3188.
30. Carroll CL, Schramm CM, Zucker AR. Slow-responders to IV beta2-adrenergic agonist therapy: defining a novel phenotype in pediatric asthma. *Pediatr Pulmonol*. 2008;43(7):627-633.
31. Liggett SB. Polymorphisms of the beta2-adrenergic receptor and asthma. *Am J Respir Crit Care Med*. 1997;156(4 Pt 2):S156-162.
32. Thakkinstian A, McEvoy M, Minelli C, Gibson P, Hancox B, Duffy D, et al. Systematic review and meta-analysis of the association between {beta}2-adrenoceptor polymorphisms and asthma: a HuGE review. *Am J Epidemiol*. 2005;162(3):201-211.
33. Choudhry S, Ung N, Avila PC, Ziv E, Nazario S, Casal J, et al. Pharmacogenetic differences in response to albuterol between Puerto Ricans and Mexicans with asthma.[see comment]. *Am J Respir Crit Care Med*. 2005;171(6):563-570.
34. Martin AC, Zhang G, Rueter K, Khoo SK, Bizzintino J, Hayden CM, et al. Beta2-adrenoceptor polymorphisms predict response to beta2-agonists in children with acute asthma. *J Asthma*. 2008;45(5):383-388.
35. Kercsmar CM, McDowell KM. Love it or lev it: levalbuterol for severe acute asthma--for now, leave it.[comment]. *J Pediatr*. 2009;155(2):162-164.
36. Weiss KB, Sullivan SD, Lyttle CS. Trends in the cost of illness for asthma in the United States, 1985-1994. *J Allergy Clin Immunol*. 2000;106(3):493-499.
37. Ducharme FM, Chalut D, Plotnick L, Savdie C, Kudirka D, Zhang X, et al. The Pediatric Respiratory Assessment Measure: a valid clinical score for assessing acute asthma severity from toddlers to teenagers. *J Pediatr*. 2008;152(4):476-480.

38. Ungar WJ, Coyte PC, Pharmacy Medication Monitoring Program Advisory B. Prospective study of the patient-level cost of asthma care in children. *Pediatr Pulmonol*. 2001;32(2):101-108.
39. Parameswaran K, Belda J, Rowe BH. Addition of intravenous aminophylline to beta2-agonists in adults with acute asthma. *Cochrane Database of Systematic Reviews*. 2000(4):CD002742.
40. Littenberg B. Aminophylline treatment in severe, acute asthma. A meta-analysis. *JAMA*. 1988;259(11):1678-1684.
41. Browne GJ, Lam LT. Single-dose intravenous salbutamol bolus for managing children with acute severe asthma in the emergency department:Reanalysis of data. *Pediatr Crit Care Med*. 2002;3(2):117-123.
42. Browne GJ, Penna AS, Phung X, Soo M. Randomised trial of intravenous salbutamol in early management of acute severe asthma in children. *Lancet*. 1997;349(9048):301-305.
43. Browne GJ, Trieu L, Van Asperen P. Randomized, double-blind, placebo-controlled trial of intravenous salbutamol and nebulized ipratropium bromide in early management of severe acute asthma in children presenting to an emergency department. *Crit Care Med*. 2002;30(2):448-453.
44. Spivey WH, Skobeloff EM, Levin RM. Effect of magnesium chloride on rabbit bronchial smooth muscle. *Ann Emerg Med*. 1990;19(10):1107-1112.
45. Iseri LT, French JH. Magnesium: nature's physiologic calcium blocker. *Am Heart J*. 1984;108(1):188-193.
46. Hill J, Britton J. Dose-response relationship and time-course of the effect of inhaled magnesium sulphate on airflow in normal and asthmatic subjects. *Br J Clin Pharmacol*. 1995;40(6):539-544.
47. Rolla G, Bucca C, Brussino L, Colagrande P. Effect of intravenous magnesium infusion on salbutamol-induced bronchodilatation in patients with asthma. *Magnes Res*. 1994;7(2):129-133.
48. Cairns CB, Kraft M. Magnesium attenuates the neutrophil respiratory burst in adult asthmatic patients.[see comment]. *Acad Emerg Med*. 1996;3(12):1093-1097.
49. Mohammed S, Goodacre S. Intravenous and nebulised magnesium sulphate for acute asthma: systematic review and meta-analysis. *Emerg Med J*. 2007;24(12):823-830.
50. Cheuk DK, Chau TC, Lee SL. A meta-analysis on intravenous magnesium sulphate for treating acute asthma. *Arch Dis Child*. 2005;90(1):74-77.
51. Global Initiative for Asthma. www.ginasthma.com. Accessed October 5, 2009.
52. Canadian Asthma Consensus Group. Management of patients with asthma in the emergency department and in hospital. *CMAJ Canadian Medical Association Journal*. 1999;161(11 Suppl):S53-59.
53. British Thoracic Society and Scottish Intercollegiate Guidelines Network. British guideline on the management of asthma. http://www.brit-thoracic.org.uk/Portals/0/Clinical%20Information/Asthma/Guidelines/sign101%20revised%20June%2009.pdf. Published 2009. Updated June 2009. Accessed October 23, 2009.
54. Schuh S, Macias C, Freedman S, Plint A, Zorc J, Bajaj L, et al. North American Practice Patterns of IV Magnesium Therapy in Severe Acute Asthma in Children. *Acad Emerg Med*. 2010;17(11):1189-1196.
55. Taddio A, Soin HK, Schuh S, Koren G, Scolnik D. Liposomal lidocaine to improve procedural success rates and reduce procedural pain among children: a randomized controlled trial.[see comment]. *CMAJ Canadian Medical Association Journal*. 2005;172(13):1691-1695.
56. Black KJ, Pusic MV, Harmidy D, McGillivray D. Pediatric intravenous insertion in the emergency department: bevel up or bevel down? *Pediatr Emerg Care*. 2005;21(11):707-711.
57. Arts SE, Abu-Saad HH, Champion GD, Crawford MR, Fisher RJ, Juniper KH, et al. Age-related response to lidocaine-prilocaine (EMLA) emulsion and effect of music distraction on the pain of intravenous cannulation. *Pediatrics*. 1994;93(5):797-801.

58. Manner T, Kanto J, Iisalo E, Lindberg R, Viinamaki O, Scheinin M. Reduction of pain at venous cannulation in children with a eutectic mixture of lidocaine and prilocaine (EMLA cream): comparison with placebo cream and no local premedication. *Acta Anaesthesiol Scand*. 1987;31(8):735-739.
59. Taddio A, Gurguis MG, Koren G. Lidocaine-prilocaine cream versus tetracaine gel for procedural pain in children. *Ann Pharmacother*. 2002;36(4):687-692.
60. Walco GA, Burns JP, Cassidy RC. The ethics of pain control in infants and children. In: Schechter NL, Berde CB, Yaster M, editors. *Pain in infants, children, and adolescents*. 2nd ed. New York: Lippincott Williams & Wilkins; 2003. p. 157-168.
61. Magnesium Sulphate: Adverse Effects. In: Reynolds JEF, Parfitt K, Parsons AV, Sweetman SC, Council of the Royal Pharmaceutical Society of Great Britain, Department of Pharmaceutical Sciences, editors. *The extra pharmacopoeia*. 29th ed. London: Pharmaceutical Press; 1989. p. 1033.
62. Okayama H, Aikawa T, Okayama M, Sasaki H, Mue S, Takishima T. Bronchodilating effect of intravenous magnesium sulfate in bronchial asthma. *JAMA*. 1987;257(8):1076-1078.
63. Hughes R, Goldkorn A, Masoli M, Weatherall M, Burgess C, Beasley R. Use of isotonic nebulised magnesium sulphate as an adjuvant to salbutamol in treatment of severe asthma in adults: randomised placebo-controlled trial.[see comment]. *Lancet*. 2003;361(9375):2114-2117.
64. Mahajan P, Haritos D, Rosenberg N, Thomas R. Comparison of nebulized magnesium sulfate plus albuterol to nebulized albuterol plus saline in children with acute exacerbations of mild to moderate asthma. *J Emerg Med*. 2004;27(1):21-25.
65. Aggarwal P, Sharad S, Handa R, Dwiwedi SN, Irshad M. Comparison of nebulised magnesium sulphate and salbutamol combined with salbutamol alone in the treatment of acute bronchial asthma: a randomised study. *Emergency Medicine Journal*. 2006;23(5):358-362.
66. Drobina BJ, Kostic MA, Roos JA. Nebulized magnesium has no benefit in the treatment of acute asthma in the emergency department. *Acad Emerg Med*. 2006;13(S26).
67. Kokturk N, Turktas H, Kara P, Mullaoglu S, Yilmaz F, Karamercan A. A randomized clinical trial of magnesium sulphate as a vehicle for nebulized salbutamol in the treatment of moderate to severe asthma attacks.[see comment]. *Pulm Pharmacol Ther*. 2005;18(6):416-421.
68. Bessmertny O, DiGregorio RV, Cohen H, Becker E, Looney D, Golden J, et al. A randomized clinical trial of nebulized magnesium sulfate in addition to albuterol in the treatment of acute mild-to-moderate asthma exacerbations in adults. *Ann Emerg Med*. 2002;39(6):585-591.
69. Nannini LJ, Pendino JC, Corna RA, Mannarino S, Quispe R. Magnesium sulfate as a vehicle for nebulized salbutamol in acute asthma. *The American journal of medicine*. 2000;108(3):193-197.
70. Blitz M, Blitz S, Hughes R, Diner B, Beasley R, Knopp J, et al. Aerosolized magnesium sulfate for acute asthma: a systematic review.[see comment][erratum appears in Chest. 2005 Nov;128(5):3779]. *Chest*. 2005;128(1):337-344.
71. Blitz M, Blitz S, Beasely R, Diner BM, Hughes R, Knopp JA, et al. Inhaled magnesium sulfate in the treatment of acute asthma.[update in Cochrane Database Syst Rev. 2005;(4):CD003898; PMID: 16235345][update of Cochrane Database Syst Rev. 2005;(2):CD003898; PMID: 15846687]. *Cochrane Database of Systematic Reviews*. 2005(3):CD003898.
72. Coates A, Leung K, Vecellio None L, Schuh S. The Choice of a Nebulizer for Delivering Magnesium Sulfate to Pediatric Asthmatic Patients in the Emergency Department. *Respir Care*. In Press.
73. Coates AL, MacNeish CF, Allen PD, Ho SL, Lands LC. Do sinusoidal models of respiration accurately reflect the respiratory events of patients breathing on nebulizers? *J Aerosol Med*. 1999;12(4):265-273.
74. Katz SL, Adatia I, Louca E, Leung K, Humpl T, Reyes JT, et al. Nebulized therapies for childhood pulmonary hypertension: an in vitro model. *Pediatr Pulmonol*. 2006;41(7):666-673.

75. Coates AL, Dinh L, MacNeish CF, Rollin T, Gagnon S, Ho SL, et al. Accounting for radioactivity before and after nebulization of tobramycin to insure accuracy of quantification of lung deposition. *J Aerosol Med*. 2000;13(3):169-178.
76. Coates AL, Tipples G, Leung K, Gray M, Louca E, Vaccine WHOPDGfMA. How many infective viral particles are necessary for successful mass measles immunization by aerosol? *Vaccine*. 2006;24(10):1578-1585.
77. Mitchell JP, Nagel MW, Nichols S, Nerbrink O. Laser diffractometry as a technique for the rapid assessment of aerosol particle size from inhalers. *J Aerosol Med*. 2006;19(4):409-433.
78. Coates AL, Macneish CF, Lands LC, Smountas A, Meisner D, Kelemen S, et al. Factors influencing the rate of drug output during the course of wet nebulization. *Journal of Aerosol Medicine-Deposition Clearance and Effects in the Lung*. 1998;11(2):101-111.
79. Dodd ME, Abbott J, Maddison J, Moorcroft AJ, Webb AK. Effect of tonicity of nebulised colistin on chest tightness and pulmonary function in adults with cystic fibrosis. *Thorax*. 1997;52(7):656-658.
80. Ciarallo L, Brousseau D, Reinert S. Higher-dose intravenous magnesium therapy for children with moderate to severe acute asthma. *Arch Pediatr Adolesc Med*. 2000;154(10):979-983.
81. Chalut DS, Ducharme FM, Davis GM. The Preschool Respiratory Assessment Measure (PRAM): a responsive index of acute asthma severity.[see comment]. *J Pediatr*. 2000;137(6):762-768.
82. Chua HL, Collis GG, Newbury AM, Chan K, Bower GD, Sly PD, et al. The influence of age on aerosol deposition in children with cystic fibrosis. *Eur Respir J*. 1994;7(12):2185-2191.
83. Newhouse MT, Dolovich MB. Control of asthma by aerosols.[see comment]. *N Engl J Med*. 1986;315(14):870-874.
84. Tal A, Golan H, Grauer N, Aviram M, Albin D, Quastel MR. Deposition pattern of radiolabeled salbutamol inhaled from a metered-dose inhaler by means of a spacer with mask in young children with airway obstruction. *J Pediatr*. 1996;128(4):479-484.
85. Wildhaber JH, Dore ND, Wilson JM, Devadason SG, LeSouef PN. Inhalation therapy in asthma: nebulizer or pressurized metered-dose inhaler with holding chamber? In vivo comparison of lung deposition in children.[see comment]. *J Pediatr*. 1999;135(1):28-33.
86. Leung K, Louca E, Coates AL. Comparison of breath-enhanced to breath-actuated nebulizers for rate, consistency, and efficiency. *Chest*. 2004;126(5):1619-1627.
87. Wildhaber JH, Devadason SG, Hayden MJ, Eber E, Summers QA, LeSouef PN. Aerosol delivery to wheezy infants: a comparison between a nebulizer and two small volume spacers. *Pediatr Pulmonol*. 1997;23(3):212-216.
88. Qureshi F, Pestian J, Davis P, Zaritsky A. Effect of nebulized ipratropium on the hospitalization rates of children with asthma. *N Engl J Med*. 1998;339(15):1030-1035.
89. Kopach R, Sadat S, Gallaway ID, Geiger G, Ungar WJ, Coyte PC. Cost-effectiveness analysis of medical documentation alternatives. *Int J Technol Assess Health Care*. 2005;21(1):126-131.
90. Brand PL, Baraldi E, Bisgaard H, Boner AL, Castro-Rodriguez JA, Custovic A, et al. Definition, assessment and treatment of wheezing disorders in preschool children: an evidence-based approach. *Eur Respir J*. 2008;32(4):1096-1110.
91. Ducharme FM, Lemire C, Noya FJ, Davis GM, Alos N, Leblond H, et al. Preemptive use of high-dose fluticasone for virus-induced wheezing in young children.[see comment]. *N Engl J Med*. 2009;360(4):339-353.
92. Oommen A, Lambert PC, Grigg J. Efficacy of a short course of parent-initiated oral prednisolone for viral wheeze in children aged 1-5 years: randomised controlled trial.[see comment]. *Lancet*. 2003;362(9394):1433-1438.

93. Vuillermin PJ, Robertson CF, South M. Parent-initiated oral corticosteroid therapy for intermittent wheezing illnesses in children: systematic review.[see comment]. *J Paediatr Child Health*. 2007;43(6):438-442.
94. Johnston SL, Pattemore PK, Sanderson G, Smith S, Lampe F, Josephs L, et al. Community study of role of viral infections in exacerbations of asthma in 9-11 year old children.[see comment]. *BMJ*. 1995;310(6989):1225-1229.
95. Martinez FD, Wright AL, Taussig LM, Holberg CJ, Halonen M, Morgan WJ. Asthma and wheezing in the first six years of life. The Group Health Medical Associates.[see comment]. *N Engl J Med*. 1995;332(3):133-138.
96. Castro-Rodriguez JA, Holberg CJ, Wright AL, Martinez FD. A clinical index to define risk of asthma in young children with recurrent wheezing. *Am J Respir Crit Care Med*. 2000;162(4 Pt 1):1403-1406.
97. Miller MR, Hankinson J, Brusasco V, Burgos F, Casaburi R, Coates A, et al. Standardisation of spirometry. *Eur Respir J*. 2005;26(2):319-338.
98. Birken CS, Parkin PC, Macarthur C. Asthma severity scores for preschoolers displayed weaknesses in reliability, validity, and responsiveness. *J Clin Epidemiol*. 2004;57(11):1177-1181.
99. Panickar J, Lakhanpaul M, Lambert PC, Kenia P, Stephenson T, Smyth A, et al. Oral prednisolone for preschool children with acute virus-induced wheezing. *N Engl J Med*. 2009;360(4):329-338.
100. Ducharme FM, Davis GM. Respiratory resistance in the emergency department: a reproducible and responsive measure of asthma severity.[see comment]. *Chest*. 1998;113(6):1566-1572.
101. Gorelick MH, Stevens MW, Schultz TR, Scribano PV. Performance of a novel clinical score, the Pediatric Asthma Severity Score (PASS), in the evaluation of acute asthma. *Acad Emerg Med*. 2004;11(1):10-18.
102. Akinbami LJ, Schoendorf KC. Trends in childhood asthma: prevalence, health care utilization, and mortality. *Pediatrics*. 2002;110(2 Pt 1):315-322.
103. Fleiss JL. *Statistical methods for rates and proportions*. 2nd ed. New York, NY: Wiley; 1981.
104. Schuh S, Canny G, Reisman JJ, Kerem E, Bentur L, Petric M, et al. Nebulized albuterol in acute bronchiolitis. *J Pediatr*. 1990;117(4):633-637.
105. Schuh S, Reider MJ, Canny G, Pender E, Forbes T, Tan YK, et al. Nebulized albuterol in acute childhood asthma: comparison of two doses. *Pediatrics*. 1990;86(4):509-513.
106. Bentur L, Canny GJ, Shields MD, Kerem E, Schuh S, Reisman JJ, et al. Controlled trial of nebulized albuterol in children younger than 2 years of age with acute asthma. *Pediatrics*. 1992;89(1):133-137.
107. Schuh S, Johnson D, Canny G, Reisman J, Shields M, Kovesi T, et al. Efficacy of adding nebulized ipratropium bromide to nebulized albuterol therapy in acute bronchiolitis. *Pediatrics*. 1992;90(6):920-923.
108. Schuh S, Johnson DW, Callahan S, Canny G, Levison H. Efficacy of frequent nebulized ipratropium bromide added to frequent high-dose albuterol therapy in severe childhood asthma. *J Pediatr*. 1995;126(4):639-645.
109. Johnson DW, Schuh S, Koren G, Jaffee DM. Outpatient treatment of croup with nebulized dexamethasone. *Arch Pediatr Adolesc Med*. 1996;150(4):349-355.
110. Johnson DW, Jacobson S, Edney PC, Hadfield P, Mundy ME, Schuh S. A comparison of nebulized budesonide, intramuscular dexamethasone, and placebo for moderately severe croup. *N Engl J Med*. 1998;339(8):498-503.
111. Schuh S, Johnson DW, Stephens D, Callahan S, Winders P, Canny GJ. Comparison of albuterol delivered by a metered dose inhaler with spacer versus a nebulizer in children with mild acute asthma. *J Pediatr*. 1999;135(1):22-27.

112. Schuh S, Reisman J, Alshehri M, Dupuis A, Corey M, Arseneault R, et al. A comparison of inhaled fluticasone and oral prednisone for children with severe acute asthma. *N Engl J Med*. 2000;343(10):689-694.
113. Schuh S, Coates AL, Binnie R, Allin T, Goia C, Corey M, et al. Efficacy of oral dexamethasone in outpatients with acute bronchiolitis. *J Pediatr*. 2002;140(1):27-32.
114. Scolnik D, Coates AL, Stephens D, Da Silva Z, Lavine E, Schuh S. Controlled delivery of high vs low humidity vs mist therapy for croup in emergency departments: a randomized controlled trial. *JAMA*. 2006;295(11):1274-1280.
115. Schuh S, Dick PT, Stephens D, Hartley M, Khaikin S, Rodrigues L, et al. High-dose inhaled fluticasone does not replace oral prednisolone in children with mild to moderate acute asthma. *Pediatrics*. 2006;118(2):644-650.

Appendix A: Pediatric Respiratory Assessment Measure (PRAM) Score

Signs	0	1	2	3
Suprasternal retractions	Absent		Present	
Scalene muscle contraction	Absent		Present	
Air entry*	Normal	Decreased at bases	Widespread decrease	Absent/minimal
Wheezing*	Absent	Expiratory only	Inspiratory and expiratory	Audible without stethoscope/ silent chest with minimal air entry
O2 saturation	≥95%	92%-94%	<92%	
*If asymmetric findings between the right and left lungs, the most severe side is rated. Reprinted from The Journal of Pediatrics, Vol 137, Issue 6, Chalut DS, Ducharme FM, Davis, GM. The Preschool Assessment Measure (PRAM): A responsive index of acute asthma severity. Pages 762-768, Copyright © 2000 with permission from Elsevier.				

Appendix B: <u>Pediatric Respiratory Assessment Measure (PRAM) Scores in Triage and After Initial Bronchodilator Therapy*</u>

**Triage PRAM**:	(N)	**Post-Bronchodilator Therapy PRAM ≥ 5**
4:	74	16 (22%)
5:	69	24 (35%)
6:	88	45 (51%)
7:	50	34 (68%)
8:	32	25 (78%)
9:	18	15 (83%)
10:	10	8 (80%)
11:	11	11 (100%)

Of children with PRAM ≥5 in triage, 58% (162/278) have post-bronchodilator therapy PRAM of ≥ 5.

<u>Probability of Hospitalization with different post-bronchodilator therapy PRAM scores*</u>

PRAM ≥ 4:	61/290 = 21%
PRAM ≥ 5:	53/184 = 30%
PRAM ≥ 6:	45/113 = 40%

<u>Post-Bronchodilator PRAM score as a Proportion of Asthma Hospitalizations*</u>

PRAM ≥ 4:	97%
PRAM ≥ 5:	84%
PRAM ≥ 6:	71%
PRAM ≥ 7:	49%

*2006 Asthma Audit from a Canadian pediatric ED

Appendix C: LOGISTICS OF BLINDING AND KIT MAKING

	**Investigational Drug or Placebo (mg=mL) (provided in a blinded vial)**	**Salbutamol Nebulizer Solution 5mg/mL (mg=mL)**	**Diluent Volume to Top up to 6mL Final Volume (mL)**	Osmolarity (mOsm/L)
Active Arm	Magnesium Sulfate Injection 500mg/mL (600mg Mg Sulf = 1.2mL)	5mg = 1mL	Sterile Water for Injection (3.8mL)	**384**
Placebo Arm	Hypertonic Saline (5.5%) (0mg Mg Sulf = 1.2mL)	5mg = 1mL	Sterile Water for Injection (3.8mL)	**381**

Each numbered kit, numbered according to the site's Master Randomization table, will contain 3 vials:
a) **Magnesium Sulfate** Injection 500mg/mL (600mg MgSulf = 1.2mL) **for Active Kits** OR **Hypertonic Saline** (5.5%) (0mg Mg Sulf = 1.2mL) **for Placebo Kits**

- The Magnesium Sulfate will be repackaged from their original vials into empty sterile vials in a laminar air flow hood.
- ***Hypertonic Saline (5.5%) was chosen to be included in the Placebo since Magnesium Sulfate itself (in the active arm) is very hypertonic. This is the percentage that mimicks the osmolality of the Active Magnesium.***
- Hypertonic Saline (5.5%) [to be used in the placebo arm only] will be compounded by pharmacy in a Laminar Air Flow hood using 14.6% concentrated Sodium Chloride and sterile water according to a strict standard and SOPs.
  - The repackaged Mg Sulfate and compounded placebo vials will be given a 6 month expiry date.
  - Identical labels will be placed on the blinded vials in order to ensure the integrity of the blind.

b) Salbutamol Nebulizer Solution 5mg/mL

- Canadian commercial supply in an open label fashion. No blinding required.

c) Top up diluent to top up to final 6mL nebulizer volume

- Canadian commercial supply of sterile water for Injection in an open label fashion. No blinding required.
- ***Sterile Water was chosen as the top up diluent to ensure that the osmolality of the nebulizer solutions was less than 500 (the osmolality at which bronchospasm has been reported). We did not chose normal saline as a diluents since this would have increased the osmolarity of the combination nebulizer solutions in both arms.***

In this Investigator initiated study, the numbered kits will be assembled and labeled in the Research Pharmacy according to detailed kit making Standard Operating Procedures. All kits/products will have appropriate Clinical Trial labeling according to Canadian regulations.

Appendix D: EMERGENCY UNBLINDING PROCEDURES

In the unlikely event the patient develops hypotension requiring therapy, apnea, heart block or another adverse event and the ED physician feels that the experimental therapy cannot be safely continued, further doses of the experimental treatment will be stopped.

If these adverse events are accompanied by severe distress and additional IV Mg is warranted, the study may be unblinded for that subject. If the subject was allocated to the Active Mg Sulfate arm, then additional IV Mg should not be given but alternative treatment provided instead. If the subject was allocated to the Placebo arm, then IV Mg may be given as part of treatment of the adverse event.

Emergency unblinding should only be requested when the clinical treatment of the patient will be different by knowing which arm of the study the patient was previously on. The study PI and the study nurses will remain blinded if possible.

The following Emergency Unblinding procedure will be followed:

1. Treating Physician or RN should contact the PI of the study for consultation to unblind. In the event they cannot be reached immediately go to the next step.
2. Contact the SickKids hospital pharmacy by phone.
3. Provide the patient's study randomization number, reason for unblinding, your site and your name to the SickKids pharmacist who will then provide the unblinded study arm.
4. Note that all patients whose therapy is unblinded must stop taking the experimental therapy The ED physician will prescribe additional treatment as clinically appropriate.
5. The requesting physician should initiate Email communication within 24 hours detailing the request for Emergency unblinding and why. The email must inform the local PI, study PI and SickKids Research Pharmacist.
6. The DSMC and REB will be advised of emergency unblinding within 48 hours.

Appendix E: ENROLLMENT FEASIBILITY (TORONTO SITE)

Total number of asthmatics ≥ 2 years of age annually (September through May)
2,382

↓

250 admissions

↓

2,132 discharges

↓

210 (84% of 250) of admissions/ have PRAM ≥ 5 after 3 treatments
640 (30% of 2,132) of discharges have PRAM ≥ 5 after 3 treatments

↓

Total 850 have PRAM ≥ 5 after 3 treatments

↓

364 present 6days a week, 12 hours a day

↓

Worst-Case Scenario
Misses (10%)

328 (not missed)

↓

Exclusions (30%)
230 (eligible)

↓

No Consent (40%)
138 (consenting & randomized)

↓

Loss to Follow-Up (5%)
Analyzable in 9 months
131

Appendix E1: ENROLLMENT FEASIBILITY (CALGARY SITE)

Total number of asthmatics ≥ 2 years of age annually (September through May)
2,000

↓

200 admissions

↓

1,800 discharges

↓

168 (84% of 200) of admissions/ have PRAM ≥ 5 after 3 treatments
540 (30% of 1,800) of discharges have PRAM ≥ 5 after 3 treatments

↓

Total 708 have PRAM ≥ 5 after 3 treatments

↓

151 present 3 days a week, 12 hours a day

↓

Worst-Case Scenario
Misses (10%)

136 (not missed)

↓

Exclusions (30%)
95 (eligible)

↓

No Consent (40%)
57(consenting & randomized)

↓

Loss to Follow-Up (5%)
Analyzable in 9 months
54

Magnesium Nebulization Utilization in Management of Pediatric Asthma – "MagNUM PA"

Principal Investigator:

Dr. Suzanne Schuh: The Hospital for Sick Children, Toronto, Ontario

Co-Investigators:

Dr. David Johnson: Alberta Children's Hospital, Calgary, Alberta

Dr. Roger Zemek: Children's Hospital of Eastern Ontario (CHEO), Ottawa, Ontario

Dr. Stephen Freedman: Alberta Children's Hospital, Calgary, Alberta

Dr. Darcy Beer: Winnipeg Children's Hospital, Winnipeg, Manitoba

Dr. Amy Plint: Children's Hospital of Eastern Ontario (CHEO), Ottawa, Ontario

Dr. Karen Black: BC Children's Hospital, Vancouver, British Columbia

Dr. Andrew Willan: The Hospital for Sick Children, Toronto, Ontario

Dr. Francine Ducharme: CHU – Sainte-Justine Pédiatrie, Montreal, Quebec

Dr. Graham Thompson: Alberta Children's Hospital, Calgary, Alberta

Dr. Allan Coates: The Hospital for Sick Children, Toronto, Ontario

Dr. Jocelyn Gravel: CHU – Sainte-Justine Pédiatrie, Montreal, Quebec

Dr. Sarah Curtis: Edmonton Clinic Health Academy, University of Alberta

Collaborators:

Dr. Naveen Poonai: Children's Hospital London Health Sciences Centre, London, Ontario

Darcy Nicksy: The Hospital for Sick Children, Toronto, Ontario

Magnesium Nebulization Utilization in Management of Pediatric Asthma – "MagNUM PA"

BACKGROUND AND SIGNIFICANCE

Acute asthma is a leading cause of pediatric emergency visits and hospitalizations.[1] In 2005, there were 754,000 pediatric ED asthma visits in the US[2,3], 15-20% of these require hospitalization and another 10-20% relapse within two weeks.[4] However, a 2006 asthma audit of a Canadian pediatric ED shows that 30% of children remaining in moderate and severe distress following initial stabilization therapy are hospitalized and that this population represents 84% of all children admitted to hospital with acute asthma.

Standard therapy of acute severe asthma consists of frequent inhaled $ß_2$ agonists, anticholinergics and corticosteroids.[5-15] However, this regimen has a high degree of outcome heterogeneity since the full benefit of corticosteroids is often not apparent until well beyond the purported 4 hour interval[16] and a substantial proportion of children responds poorly to $ß_2$ agonists (this resistance is in part determined by $ß_2$ adrenoreceptor gene polymorphism).[16-34] Current stabilization therapy[5,14,15] is not always effective in severe attacks[35] and related costs remain high.[36] *Since these insufficient/poor responders represent virtually all pediatric asthma hospitalizations[37] and since hospitalizations account for 43% of the pediatric asthma care costs[38], finding effective strategies to decrease their morbidity is imperative.* Two adjunctive interventions poorly explored in the acute care setting are not ideal for the ED – IV methylxanthines are associated with significant toxicity and no longer recommended[39,40] and IV $ß_2$ agonists are generally reserved for ICU.[41-43]

Mg is a powerful relaxant of airway smooth muscle[44], with a rapid effect when given IV. It relieves bronchoconstriction by decreasing the uptake and release of calcium in bronchial smooth muscle[45], inhibiting release of acetylcholine[46] and of histamine release and stimulating nitric oxide and prostaglandin synthesis.[46] Furthermore, Mg augments the effect of $ß_2$ agonists by upregulating $ß_2$ receptors[47] and also reduces neutrophilic burst seen with the inflammatory response.[48] Mg can be given either IV or by nebulization. Two key meta-analyses confirm that the addition of IV Mg to routine therapy significantly improves hospitalizations and lung function.[49,50]The authors and several major asthma guidelines recommend that IV Mg be considered in children not responding to initial management.[49,51-53] However, our survey "North American Practice Patterns of IV Mg in Severe Acute Asthma in Children" showed that 24% of participants have personally witnessed an Mg-attributed hypotension requiring treatment which, along with the belief that most children with asthma improve without an IV constitute major barriers to the use of IV Mg.[54] These results suggest that adverse effects of IV Mg may not be rare. Furthermore, IV access is much more difficult in young children (who make up the majority of children with asthma) than in adults, and multiple attempts are often required which can lead to an increasing cycle of crying and severe respiratory distress.[55-60] Other theoretical adverse effects after IV Mg administration include apnea and heart block.[61] However, none of the IV or inhaled Mg trials has reported either of these complications.

In contrast, the nebulization route is non-invasive and offers a major advantage of targeted delivery to the lower airway and less potential for side-effects,[62] due to a lower systemic delivery

of Mg (1/4 of the IV dose). With IV delivery of Mg, the greatest tissue exposure within the lung is in the alveoli and Mg has to diffuse from the thicker-walled pulmonary and bronchial circulation to reach the smooth muscles of the airways. In contrast, most inhaled Mg would be deposited in the airways and direct diffusion through airway epithelium would result in much higher Mg levels around the smooth muscle as compared with IV delivery. However, the investigation of the efficacy of nebulized Mg has been sparse and has yielded disparate results. Seven studies have compared the benefit of adding nebulized Mg to salbutamol to salbutamol alone[63-69]; only one was limited to children.[64] Almost all studies included asthmatics with negligible admission rates and only one study[63] limited participants to non-responders to bronchodilators who are most likely to benefit from nebulized Mg. This key study by Hughes et al (52 adults) showed a 30% risk reduction in hospitalizations favoring Mg (71% in controls and 43% in the Mg arm).[63] One small study of 62 school-aged children with acute asthma[64] found that a single dose of nebulized Mg added to salbutamol and systemic corticosteroids was associated with a significant improvement in FEV_1 compared to standard therapy at 10 minutes. However, ipratropium was not used, only one patient in each group was hospitalized and the authors did not examine the impact of Mg on other patient outcomes. A recent large RCT demonstrated a significant inhaled Mg effect on an asthma severity score at 60 minutes[70]but did not focus on hospitalizations and the authors did not exclude children who responded to baseline Rx.

A Cochrane systematic review by Blitz[71,72]evaluated 6 trials, 4 of which compared nebulized Mg with $ß_2$ agonists to $ß_2$ agonists alone.[63,64,68,69] There was a clear additive benefit of Mg and salbutamol on lung function in adults with severe disease and a trend towards benefit with respect to lung function and hospitalizations in moderate asthma. A later systematic review[49] of 7 studies[63-69] found an overall treatment effect of Mg and $ß_2$ agonists on both the respiratory function and hospitalization rate approaching statistical significance (p values 0.08 and 0.06, respectively). A recent Cochrane review found improved lung function and a trend toward fewer admissions in patients who received evidence-based baseline therapy[73] and attributes the lack of clear conclusions of inhaled Mg benefit to a small number of patients who were given optimized therapy, i.e. oral steroids with both salbutamol and ipratropium (total N= 247), with concurrent lack of power for using hospitalization as an outcome (N=249). **The *main limitations of past studies are inadequate use of anticholinergics, lack of limiting participants to non-responders to bronchodilators and possible use of inefficient delivery methods.***
The delivery systems used were poorly described and were of low efficiency. Given the encouraging preliminary evidence of benefit, the non-invasiveness and high safety likelihood of the nebulization route and the expertise of our team to ensure Mg delivery, a pediatric study is needed to define the role of nebulized Mg.[49,71] Addition of nebulized Mg should decrease hospitalizations in asthmatic children remaining in moderate to severe distress after optimized baseline treatment which would immediately impact current clinical practice and decrease morbidity of this high-risk population

We have obtained a peer-reviewed grant for a two-centre version of this trial from the Thrasher Research Fund which has enrolled 124 patients and shows excellent feasibility, lack of side effects and 100% compliance, with no loss to follow up. However, the rate of hospitalizations in this study is higher than anticipated hence the proposed sample size is inadequate to reliably detect a

minimum clinically significant difference in hospitalizations. For this reason, we shall need substantially larger sample size (816) to achieve definitive results. This is critically important as preliminary evidence regarding both effectiveness and safety of Mg warrants an adequately powered study.

In view of these arguments, we have submitted this proposal to the RCT committee at the Canadian Institutes for Health Research in March 2013. The study was funded in June 2013 as a Canada-wide seven-center RCT, to commence in the summer of 2014.

Acute asthma is the most common cause of pediatric hospitalizations. While we know that repeat inhalations of $ß_2$ agonists and ipratropium with early oral steroids substantially reduce hospitalizations, many children are resistant to this standard initial therapy. About a third of children remaining in moderate to severe distress after standard therapy are admitted to hospital and comprise 84% of pediatric acute asthma hospitalizations. ***Finding safe, non-invasive, and effective strategies to treat children resistant to standard therapy would substantially decrease hospitalizations resulting in considerable health care savings and reduction of the psycho-social burden of the disease.*** While studies of magnesium sulfate (Mg) given intravenously (IV) suggest that this agent can reduce hospitalizations in both adults and children resistant to standard initial therapy, a North America-wide survey completed by us shows that only 7% of Emergency Department (ED) physicians give IV Mg to prevent hospitalizations, less than 5% of children given IV Mg go home from the ED, and IV Mg is primarily used by physicians to prevent admissions to the ICU. Barriers to IV Mg use include concern about side effects, with 24% of physicians reporting having observed IV Mg-related hypotension requiring treatment as well as a belief that IV therapy is unnecessary. Nebulization is an alternate route for administering Mg. This route has the advantage of being non-invasive and is likely much safer due to lower systemic delivery. Direct delivery via nebulization allows higher Mg concentrations at the target site, the lower airways, with a smaller total drug dose. Two meta-analyses of studies of nebulized Mg – all but one of which have focused on adults - have found that its effect on hospitalizations approaches statistical significance ($p=0.08$). As a result, the authors of these meta-analyses have ***called for a properly designed study to clarify the role of nebulized Mg. This definitive trial of children in significant respiratory distress after optimized initial therapy will assess the impact of inhaled Mg on hospitalizations, use of medical resources and additional rescue co-interventions***

We plan the following specific aims:

1. **<u>Primary Objective:</u>** To examine if in children with acute asthma remaining in moderate to severe respiratory distress despite maximized initial bronchodilator and steroid therapy there is a reduction in hospitalization rate from the ED in those who receive nebulized Mg with salbutamol versus those receiving salbutamol only.
   <u>Hypothesis</u>: We hypothesize that the children with Pediatric Respiratory Assessment Measure (PRAM) ≥ 5 points after optimized initial inhaled bronchodilator and oral steroid therapies who are given nebulized Mg in addition to nebulized salbutamol will have

significantly lower hospitalization rate within 24 hours of starting the study compared to those given salbutamol only.

2. To compare a difference in the changes in the validated Pediatric Respiratory Assessment Measure (PRAM), respiratory rate, oxygen saturation and blood pressure from randomization baseline to 240 minutes in the two groups
3. To determine if there is a significant association between the difference in the primary outcome between the groups and the patient's age, gender, baseline PRAM score, personal history of atopy and "viral-induced wheeze" phenotype.

Hypothesis(es) to be Tested
In this randomized, double-blind seven-centre trial, we hypothesize that children with acute asthma with a Pediatric Respiratory Assessment Measure (PRAM) of ≥ 5 points after optimized initial inhaled bronchodilator and oral steroid therapies who are given nebulized Mg in addition to nebulized salbutamol will have at least a 10 % lower hospitalization rate within 24 hours of starting the study as compared to those given salbutamol only.

SUPPORTIVE PRELIMINARY DATA

North American Practice Patterns of IV Magnesium in Severe Acute Asthma in Children (NAPP SAAC Survey)

Schuh et al, Academic Emergency Medicine, 2010; 17(11): 1189-1196.

We have published a continent-wide survey of the Pediatric Emergency Research Canada network and of Pediatric Emergency Medicine- Collaborative Research Committee consortium (US) entitled "North American Practice Patterns of IV Magnesium Therapy in Severe Acute Asthma in Children" (NAPP SAAC Survey) with the main objective of investigating the frequency of use of IV Mg in stable and critically ill children with severe acute asthma, usual therapeutic goals with respect to disposition and factors impacting the use of this intervention.

Summary of results:

• Response rate to the survey: 70% in Canada and in the United States
• Majority of physicians use IV Mg in less than 20% of children with stable severe acute asthma
• Only 7% of the ED physicians give IV Mg to prevent hospitalizations
• 71% give IV Mg to prevent ICU admission
• Less than 5% of children given IV Mg in the ED are discharged home from the ED
• 24% of the ED physicians have personally witnessed IV Mg related hypotension requiring therapy
•NotablebarrierstotheuseofIVMg:a)concernaboutsideeffectsandb)desiretoavoidanIV
• 97.0 % of physicians felt that if high quality evidence of benefit of nebulized Mg were available, they would incorporate it into their practice and 87.9 % would be willing to participate in such research.

Magnesium Use in Asthma Pharmacotherapy in Canadian Pediatric Emergency Departments: Pediatric Emergency Research Canada Study

Suzanne Schuh, MD, FRCPC[a], Roger Zemek, MD, FRCPC[b], Amy Plint, MD, FRCPC[b], Karen JL Black, MD, FRCPC[c], Stephen Freedman, MD, FRCPC[a], Robert Porter, MD, CCFP (EM)[d], Serge Gouin, MDCM, FRCPC[e], Alexandra Hernandez, MD, FRCPC[f], David Johnson, MD, FAAP [g]

Schuh et al, Pediatrics 2012, 129:852-859.

Abstract

Objectives

To examine the utilization of intravenous magnesium in Canadian pediatric Emergency Departments (EDs) in children requiring hospitalization for acute asthma and association of administration of frequent albuterol/ipratropium and timely corticosteroids with hospitalization.

Methods

Retrospective medical record review at 6 EDs of otherwise healthy children 2-17 years of age with acute asthma. Data was extracted on history, disease severity, and timing of ED stabilization treatments with inhaled albuterol, ipratropium, corticosteroids and magnesium. Primary outcome was the proportion of hospitalized children given magnesium in the ED. Secondary outcome was the ED use of "intensive therapy" in hospitalized children, defined as three albuterol inhalations with ipratropium and corticosteroids within one hour of triage.

Results

19/154 hospitalized children received magnesium (12.3**%,** 95% CI 7.1; 17.5) versus 2/962 discharged patients. Children given magnesium were more likely to have been previously admitted to ICU (OR 11.2), hospitalized within the past year (OR3.8), received corticosteroids prior to arrival (OR4.0), presented with severe exacerbation (OR 6.1) and to have been treated at one particular centre (OR 14.9). 42/90 (53%) hospitalized children were not given "intensive therapy". Children receiving "intensive therapy" were more likely to present with severe disease to EDs using asthma guidelines (ORs 8.9, 3.0). Differences in the frequencies of all stabilization treatments were significant across centers.

Conclusions

Magnesium is used infrequently in Canadian pediatric EDs in acute asthma requiring hospitalization. Many of these children also do not receive frequent albuterol and ipratropium, or early corticosteroids. Significant variability in the use of these interventions was detected.

The Choice of a Nebulizer for Delivering Magnesium Sulfate to Pediatric Asthmatic Patients in the Emergency Department

Allan L Coates1, MDCM; Kitty Leung1, BSc; Laurent Vecellio2,3, PhD ; Suzanne Schuh4, FRCPC 1 Physiology and Experimental Medicine and 4 Population Health of the Hospital for Sick Children Research Institute, University of Toronto, Toronto Canada and 2 Aerodrug, Tours F-37000 France and 3 INSERM U-618 Université François Rableais, Tours F-37000 France

Coates et al, Respiratory Care Journal, 2011; 56(3):314-8.

Abstract

Background
As the use of intravenous magnesium sulfate (MgSO4) for the treatment of refractory asthma is becoming more common, the incidence of MgSO4 related systemic hypotension is also rising. One potential therapeutic option would be to deliver the MgSO4 by aerosol. One problem with MgSO4 is that, compared to most inhaled medication which is active in the microgram range, it has a dose requirement well into the milligram range. This, along with inefficient delivery systems, may be a reason for the lack of efficacy in some of the published studies using aerosol delivery.

Methods
Prior to a multicenter asthma study in children 2-17years of age evaluating inhaled MgSO4, an *in vitro* study was conducted to choose the best possible delivery system that would be effective over the entire age range. The potential devices considered included the Pari LC Star jet nebulizer, the Omron vibrating membrane device and the AeroNeb Go® vibrating membrane device with the Idehaler® acting as a holding chamber without valves that could connect with a face mask.

Results
The Pari LC Star® had an appropriate particle size distribution but a very slow rate of output. The Omron device had an even slower rate of output and a larger particle size distribution that would have been inappropriate for smaller children. The *in vitro* estimates for lung deposition for the AeroNeb Go® with the Idehaler® were 12.1±0.8 mg/min.

Conclusions
These data would suggest that a 16 minute nebulization session of 6 mL of a solution made up of 2 mL of 500 mg/mL of MgSO4, 1 ml of 5 mg/mL of albuterol and 7 mL of sterile water using the AeroNeb Go® vibrating membrane system attached to the Idehaler® holding chamber with a face mask would maximize delivery of magnesium to the airways in severe asthma while maintaining safety from both the question of bronchospasm due to hypersomolarity of the aerosol and hypotension from systemic absorption. Therefore this device and regime is recommended for the multicenter trial of inhaled MgSO4 in children with severe asthma.

Pulmonary Deposition with a Novel Aerosol Delivery System

Allan L Coates[1,2] MDCM, Kitty Leung[1] BSc, Jeffrey Chan[3] BSc, Nancy Ribeiro[3] RTNM Martin Charron[3] MD and Suzanne Schuh[4] MD
From Physiology and Experimental Medicine, Research Institute[1], Division of Respiratory Medicine[1,2], Division of Nuclear Medicine[3] and the Department of Emergency Medicine[4] Hospital for Sick Children, University of Toronto. Toronto Canada

Abstract

Background

A problem with intravenous magnesium sulphate ($MgSO_4$) in children and adults with severe acute asthma refractory to optimized standard therapy is systemic hypotension which might be avoided with the aerosol route. However, compared to most inhaled medications which are active in the microgram range, $MgSO_4$ has a dose requirement in the milligram range. This, plus the use of inefficient delivery systems, may explain the lack of efficacy of inhaled $MgSO_4$ in some studies. Prior to a multicenter asthma study in children 2-17 years of age evaluating inhaled $MgSO_4$, an *in vitro* study suggested that the AeroNeb Go® with the Idehaler® using a face mask would have an acceptable
pulmonary delivery of approximately 12 mg/min but no *in vivo* data exist.

Methods

Since the physical characteristics of the sodium and magnesium water suspension are comparable, five adult males had the rate of deposition of normal saline measured using nuclear medicine techniques (to eliminate any Mg-associated risk). Regions of interest comprised of both lungs, the mediastinum with both the trachea and esophagus and the stomach. The measured deposition of the radiolabel was converted to the rate of drug deposition which was compared to the results from an *in vitro* model using adult respiratory patterns.

Results

The mean rate of pulmonary deposition was <u>10.8±1.9 mg/min</u> (mean±SD) which correlated with height ($r=0.83$, $p<0.05$). The reasons for this slightly lower deposition compared to the *in vitro* estimate include the exclusion of tracheal deposition which would have been included *in vitro* and exhalation of anatomical dead space aerosol which would have been captured on the inspiratory filter *in vitro*. <u>The aforementioned deposition represents 20% of the charge dose, compared to 4% deposition by conventional nebulizers.</u>

Conclusion

The AeroNeb Go® coupled with the novel holding chamber, the Idehaler® did confirm the *in vitro* deposition data in healthy adult males, within expected limits. This device appears suitable for the clinical trial of inhaled $MgSO_4$ over a wide range of ages in patients with refractory asthma. Respiratory Care, December 2013, epub ahead of print.

Development and Evaluation of PRAM[37,83]:
The vast majority of children with acute asthma are of pre-school age and lack coordination to perform pulmonary function tests reliably. Dr Ducharme and colleagues therefore developed and validated the Pediatric Respiratory Assessment Measure (PRAM) as a measure of severity of airway obstruction in acute asthma and its responsiveness to treatment and later evaluated its performance characteristics in children 2 years of age and older presenting with acute asthma in the Emergency Department setting. *This background work will provide us with the ability to use this excellent measurement tool in this trial – both as an entry-severity criterion and as a secondary outcome.*

EXPERIMENTAL DESIGN AND METHODOLOGY

Primary question:
In children 2-17 years of age with acute asthma who have persistent moderate to severe airway obstruction despite maximized initial bronchodilator and steroid therapy, is there a significant difference in the hospitalization rate in those who receive three nebulized Mg and salbutamol treatments compared to those receiving only nebulized salbutamol?

Secondary questions:
Between these treatment modalities:
a). Is there a difference in the changes in the validated Pediatric Respiratory Assessment Measure (PRAM), respiratory rate, oxygen saturation and blood pressure from randomization baseline to 240 minutes?
b). Is there a difference in the number of salbutamol treatments within 240 minutes?
c). Does the treatment effect with respect to primary outcome vary between subgroups defined by these variables: age, gender, pre-randomization PRAM score, personal history of atopy and "viral-induced wheeze" phenotype?

Hypothesis:
We hypothesize that the children with PRAM ≥ 5 points after optimized initial inhaled bronchodilator and oral steroid therapy who are given nebulized Mg in addition to nebulized salbutamol will have a significantly lower hospitalization rate at the index visit compared to those given salbutamol only.

Trial Design:
This is a seven-centre randomized double-blind placebo controlled trial. Two study groups will be compared: nebulized salbutamol with Mg sulfate and nebulized salbutamol with placebo.

Triage RN: Baseline PRAM score (**see Appendix A**) in all asthmatics
Moderate to severe disease (PRAM meets local criteria for ipratropium & corticosteroids):
Assessment by ED physician
Systemic corticosteroids
3 treatments with salbutamol & ipratropium over 1 hour
Study nurse notified

↓

Study Nurse repeats PRAM at approximately 1 hour, if ≥ 5: assess eligibility criteria.
(If PRAM is < 5, subject is ineligible for study)

↓

If eligible, obtain informed consent
Baseline measurements (BP, RR, HR, O2 Sat, Obtain treatment allocation assignment
Retrieve treatment package from ED/pharmacy

↓

Experimental Group Treatment
Mg (600 mg) and Salbutamol (5mg)
(5mg)
@ 0', 20', 40'

Control Group Treatment
Placebo and Salbutamol
@ 0', 20', 40'

↓

PRAM, RR, HR, O_2 sat @ 0'(pre experimental Rx), 60' (post experimental Rx), 120', 180', 240'
BP @ 0', 20', 40', 60', 120', 180', 240'
(These study procedures can be done +/- 10 minutes at each time point)

↓

Further bronchodilator therapy as clinically indicated

↓

Disposition decision by ED MD

↓

Electronic/paper health chart review and telephone follow-up at 72 hrs (+4 days)

Inclusion criteria:

(1) **2-17 years** of age

(2) **Diagnosis of asthma**, defined as this diagnosis made by a physician or at least one prior acute episode of wheezing with cough or dyspnea treated with inhaled $ß_2$ agonists or oral corticosteroids. *Our study population will exclude bronchiolitis and first-time wheeze (potential alternate diagnoses).*

(3) ***Persistent*** **moderate to severe airway obstruction** after 3 doses of salbutamol and ipratropium, defined as a PRAM 5 or higher. *A PRAM score of 5 or more following initial therapy indicates the child has at least moderate disease severity*[37] *and has a high likelihood of being hospitalized.*[37] *This group of children includes 84% of all pediatric asthma*

hospitalizations; therefore, finding an effective therapy for this population has great potential to significantly reduce hospitalizations. (Appendix B).

Although the inclusion of children with PRAM scores of 4 or more would enable us to capture nearly all asthma admissions, their admission rate is substantially lower (20%) and thus the overall baseline likelihood of admission would be reduced (Appendix B). Although the admission rate for children with PRAM of 6 or more is high, randomizing only this population would miss 30% of asthma hospitalizations (Appendix B). For these reasons, we have chosen to randomize children with PRAM 5 or more after initial bronchodilator therapy.

Although the PRAM scores of most children will improve following the initial treatment, 35% of those with a presenting PRAM of 5 points do not change (Appendix B). Thus, to maximize capturing this high-risk population, we shall screen and perform post-bronchodilator therapy PRAM scores on all previously healthy children in the target age-range with a presenting PRAM of 5 points or more.

Exclusion Criteria:

(1) No previous history of wheezing or bronchodilator therapy. *Some children who present with wheezing for the first time will have other diagnoses which would not be expected to respond to Mg.*
(2) Patients who have already received IV Mg therapy during the index visit.
(3) Critically ill children requiring immediate intubation. *These children need immediate ICU management and hospitalization.*
(4) Children who in the opinion of the treating physician require a chest radiograph due to atypical clinical presentation and are diagnosed to have lobar consolidation with pneumonia, felt to be the primary cause of respiratory distress. *These rare patients may have to be hospitalized primarily for treatment of the infection and may not respond to magnesium.*
(5) Known co-existent renal, chronic pulmonary, neurologic, cardiac or systemic disease. *These conditions may influence the response to Mg and hospitalization*.
(6) Known hypersensitivity to Mg sulfate.
(7) Patients previously enrolled in the study.
(8) Insufficient command of the English language.
(9) Lack of a home or cellular telephone.

Sample Selection:

Children presenting to the collaborating EDs at The Hospital for Sick Children, Children's Hospital of Eastern Ontario, Ste Justine's Hospital, London Health Sciences Centre, Alberta Children's Hospital, Stollery Hospital and Children's Hospital of Winnipeg who meet eligibility criteria will be approached for enrollment when the research nurses are on duty (days and evenings). The research nurses will keep a log of all children presenting to the ED with acute asthma during the study period whether randomized or not in order to assess the generalizability of the study. All

aforementioned hospitals are tertiary care centers, which see the entire clinical and demographic spectrum of the asthma population. Our profile of children with acute asthma should therefore be comparable to that of other institutions and the generalizability of the study should not be affected and the referral bias should be minimal. A structured data collection form will be used to assess the baseline and demographic features that may affect outcome and potentially confound the comparisons. Since the patients will be screened consecutively and study coverage will occur during days, evenings and weekends, selection bias should not play a major role.

Randomization:

The Research Coordinating Pharmacist at SickKids will produce Master Randomization tables, stratified by site and age (≥ 6 years vs less), using a permuted block randomization of 6 and 8 in a 1:1 ratio of active Mg sulfate to placebo, using random number generating software. The Master Randomization tables will be held at the Research Pharmacy at SickKids, open 24 hours a day. Consecutively numbered kits will be prepared by each pharmacy according to the step-by-step procedure manual provided by Research Coordinating Pharmacist at SickKids. Upon receiving the informed consent, the study nurse will obtain the next appropriate numbered study kit from the locked research fridge in the ED (Mg has to be refrigerated) and enter the number in the confidential log book.

Blinding:

The patients, research nurses and ED physicians will be **blinded to the treatment assignment.** The SickKids Research Pharmacist will provide a manual with detailed instructions as to how each site pharmacy will prepare blinded numbered kits containing Mg SO4 or hypertonic 5.5% saline placebo (to match tonicity of Mg Sulfate).Sites will procure a study supply of open label salbutamol nebulizer solution and sterile water to be used as a top up diluent (sterile water chosen as the diluent since mixing normal saline with Mg sulfate is hyperosmolar). Each site will be given detailed requirements for drug accountability and handling to ensure compliance with Health Canada regulations. The active Mg and placebo hypertonic saline mixture with salbutamol and sterile water are very similar in volume, color, taste and smell when nebulized (tested in the research pharmacy at SickKids). The study nurse, physicians and patient will be unaware of the next group assignment. Only the pharmacy will be unblinded. We acknowledge the remote possibility of indirect unblinding because a decrease in blood pressure may occur during Mg therapy. However, major hypotension is unlikely and the likelihood of inadequate blinding is thus very low. The current inhaled Mg study has no hypotension episodes. Study patients are usually re-assessed after conclusion of the experimental therapy unless they become unstable or a symptomatic drop in blood pressure occurs. Therefore, the ED physicians will be unaware of minor blood pressure fluctuations and the likelihood of unblinding will be minimized. To assess blinding, the research nurse and parents will be asked at the conclusion of experimental therapy which intervention they think the child had received. In case of increasing respiratory distress, IV Mg may be given after the experimental therapy, provided the patient is not hypotensive. In the unlikely event the patient develops hypotension requiring therapy or

apnea and the ED physician feels that the experimental therapy cannot be safely continued, further doses of the experimental treatment will be stopped. If these Mg side effects are also accompanied by severe distress and additional IV Mg is warranted, the code may be broken for that patient. Unblinding will only occur if the clinical treatment of the patient will change as a result of knowing which arm of the study the patient was previously on. The study PI/local PI and the study nurses will remain blinded. No patients participating in our inhaled Mg study had experimental therapy unblinded. For emergency unblinding procedures, see **Appendix D**.

Pre-Study Screening and Baseline Evaluation:

All previously healthy children 2-17 years of age with acute asthma will have a PRAM score measured in triage. Those meeting local ED criteria for enhanced therapy (with ipratropium and systemic corticosteroids) will be assessed by the ED physician and receive either oral dexamethasone, oral prednisolone/prednisone or IV hydrocortisone [all considered equivalent for reducing hospitalizations] plus three salbutamol and ipratropium inhalations via Metered Dose Inhaler/Valved Holding Chamber (MDI/VHC)/nebulizer according to the local asthma pathway 20 minutes apart. Ipratropium bromide decreases hospitalizations in asthmatic children with evidence of major distress[90], such as marked neck retractions and extensive wheeze*Our baseline initial therapy is therefore optimized and insufficient improvement/persistent respiratory distress justifies further intervention in this population.*

Study Procedures:

At approximately 1 hour, i.e. at the conclusion of the baseline three inhalations, the research nurse will assess eligibility for the study and measure the pre-randomization PRAM score. Eligible children with PRAM[83] ≥5 points after three bronchodilator treatments [at least moderate to severe respiratory distress] will be approached and informed consent will be obtained. Subjects will be randomly allocated to receive three consecutive nebulizations of salbutamol with either diluted Mg sulfate or diluted hypertonic saline placebo 20 minutes apart (+/- 10 minutes), using the Aeroneb® Go Micropump Nebulizer along with the Idehaler®Pocket system. Since three nebulizations were used in the adult study that demonstrated the greatest benefit of Mg[63], likewise we will use the same number in this study. Specifically, each treatment will utilize 600 mg (1.2 mL) of Mg sulfate (hypertonic)or 1.2 mL hypertonic 5.5% saline (*to match osmolarity of Mg sulfate-see Appendix C for details)*, 5 mg (1 mL) of salbutamol and 3.8 mL of sterile water. *Our Mg dose approximates the upper end of the Mg dosing range used in previous studies, selected to maximize the therapeutic potential of inhaled Mg. Administration of multiple experimental inhalations will have the advantage of better drug distribution in the lungs after the first treatment when some bronchoconstriction will have been relieved.*

In order to minimize the possibility of cough/bronchospasm which can on occasion be seen with inhaling solutions with osmolarities above 500 mOsm/L[81], we plan to employ a solution with an osmolarity well under 500mOsm/L. In order to ensure that any potential differences in side effects/treatment effect were not due to a difference in the osmolarity of the two solutions, we had to ensure that both the active and placebo arms solutions were of comparable and

acceptable osmolarities. *Magnesium sulphate injection solution itself is hyper-osmolar. 5.5% saline has the same osmolarity as magnesium sulphate.*

The use of ***sterile water as the top up diluent in both the active Mg/salbutamol arm and the placebo 5.5% saline/salbutamol arm yields a highly acceptable final osmolality of 384 mOsm/L in both study arms*** (Appendix C). Using normal saline as the top up diluent in the active arm would result in a higher osmolality which would exceed the upper limit of acceptability of 500 mOsm/L. Therefore, normal saline cannot be used as the top up diluent.

The use of 5.5% saline as the placebo and of sterile water as the top up diluent in both arms creates comparable experimental conditions in both study arms (Appendix C). We have also pre-tested that the Idehaler® Pocket system ® nebulizer maintains isotonicity of both active and placebo solutions throughout nebulization, thereby minimizing the possibility of side- effects.

Pre-randomization, the study nurse will measure the subject's PRAM score, respiratory rate, heart rate, oxygen saturation and blood pressure. The study nurse will measure these parameters at 60 minutes and hourly thereafter up to 240 minutes and blood pressure will also be assessed after each experimental nebulization at 20, 40, 60 minutes. These study procedures can be done +/- 10 minutes at each time point. The study nurse will also record the details of all other pharmacotherapy given as well as disposition status during the index visit. The research nurses will ascertain subsequent return visits/hospitalizations-both from the telephone follow-ups as well as from a review of the patient health records including any records from their family doctor if necessary at 72 hours. At this time the parents will also be questioned about unscheduled medical visits related to asthma and further therapies instituted. If families cannot be reached during mutually agreed upon times at 72 hours, daily phone calls will be made until day 7. If hospitalized, patients will not be contacted by the research nurse for a telephone follow-up.

Following this experimental intervention, participating children will continue to receive further salbutamol treatments as frequently as clinically warranted as per the treating ED physician. Disposition will also be determined by the ED physician, independently of the knowledge of the study intervention. If the patient has improved and the ED physician feels that he/she can go home, the patient can be discharged prior to the 240 minute study assessment. Discharged patients will receive a prescription for inhaled salbutamol via MDI up to every four hours as necessary for the next week in addition to either daily oral prednisolone/prednisone or oral dexamethasone as per local standard of care. All participating families will receive instructions to visit their primary care provider/ED if salbutamol has to be given more often than every 4 hours for increased work of breathing/severe cough and if the respiratory status interferes with usual play/normal speech or routine activity.

<u>The primary outcome measure</u> will be hospitalization defined as admission to an inpatient unit within 24 hours of the start of the experimental therapy due to continued/worsening respiratory distress. Those children in whom a decision to admit was made by the treating physician, but due to lack of bed availability were never transferred to the inpatient unit will be analyzed as admitted as will those returning to the ED within 24 hours of the start of the study who require hospitalization for asthma. It is extremely unlikely that admissions would occur primarily for reasons other than respiratory distress. The study nurse will ascertain that the hospitalizations are for respiratory distress versus other reasons. Should the latter scenario occur, these children will be identified and not counted as hospitalized. Extended ED stays without a decision to admit will not be counted as hospitalized. If the nurse leaves before disposition has been finalized he/she will review the ED electronic data records to identify the length of stay, final disposition, number of bronchodilator treatments by this time and other outcomes the next day. He/she will also communicate with the treating ED physician regarding the reason for hospitalization.

Hospitalization is a powerful marker of treatment failure, a decrease in which is likely to impact practice and influence decision makers since almost a half of pediatric asthma costs, relate to hospitalizations.[91] Hospital admission can also be a very stressful even for both the caregivers and patients. It impacts on the rest of the family since caregivers have to take time off work and arrange alternative sources of care for the other children.

<u>Secondary outcome measures</u>

The two groups will also be compared with respect to:

a. Changes in the PRAM, respiratory rate and oxygen saturation from the start of the first experimental nebulization to 60, 120, 180 and 240 minutes and the changes in the blood pressure from the first experimental nebulization to 20, 40, 60, 120, 180 and 240 minutes.
b. Number of salbutamol treatments within 240 minutes of starting experimental therapy.
c. An association between hospitalization and age, gender, pre-randomization PRAM score, personal history of atopy, and "acute viral induced wheeze" phenotype.[92] This phenotype will be defined by age less than 5 years, co-existent upper respiratory tract infection, no interval symptoms between exacerbations, no atopy. [92-98]
d. All cause hospitalization rate by 24 hours of starting Rx to examine Mg impact on side effects such as hypotension necessitating admission.

<u>Other outcomes</u>

Unscheduled visits for asthma to any medical facility within 72 hours of the start of the study. *Most return visits for acute asthma occur within this period.* However, this will be an uncommon event and a meaningful analysis may not be possible.

Major side-effects such as hypotension (systolic blood pressure below 5th percentile for age) or apnea will be tracked as will be admission to ICU for airway stabilization. These outcomes are extremely rare (unstable children will be excluded) and the study cannot therefore be powered for their meaningful statistical analysis. *However, these data are critical to estimate a safety profile of inhaled Mg in children.* We shall not measure the Forced Expiratory Volume in one second (FEV1) since most study patients will be pre-schoolers who cannot perform the necessary maneuvers reliably. Moreover, more than two thirds of the older children with severe asthma enrolled in our previous studies were unable to perform reliable lung function measurements.

PRAM is a validated 12 point clinical asthma severity score[83] exhibiting the most comprehensive measurement properties of all asthma scores[99] which has been successfully used as an outcome in major trials.[100] *It is the only score with demonstrated criterion validity, using respiratory resistance as the gold standard.*[101] This instrument has recently been validated in both preschool and school aged acute asthmatics in the ED *and has strong association with admission, thus supporting its ability to distinguish across severity levels.*[37] *The score has inter-rater reliability consistently above 70%* [37]*and is currently implemented in numerous pediatric EDs across Canada.* In contrast, the Pediatric Asthma Severity Score[102] has not been validated against a concurrent measure of lung function and may not be as responsive as the PRAM due to a smaller range. The vast majority of children treated for acute asthma are preschoolers[103] who lack sufficient coordination to perform pulmonary function tests reliably. All participating EDs now measure the PRAM score as part of routine clinical assessment in their EDs in children with acute asthma. Since Calgary is situated 1000 metres above sea level, oxygen saturations there can be expected to be approximately 2% lower than in Toronto (International Civil Aviation Organization, Manual of the ICAO Standard Atmosphere, Doc 7488-CD, Third Edition, 1993, ISBN 92-9194-004-6). Therefore, the oxygen saturation component of the PRAM will be adjusted in Calgary (this is already local practice) as outlined in Appendix A.

Study Implementation:

Prior to the study, the ED staff physicians and fellows and emergency nurses will be educated in all aspects of the study. Particular attention will be paid to the importance of communicating to the research nurse the reasons for hospitalization and the importance of protocolized stabilization therapy. The research nurses will be trained in all aspects of the study execution, including obtaining informed consent, technical aspects of administering nebulized treatments and the PRAM measurement.

This study requires the following personnel:

1. Study manager at The Hospital for Sick Children who will communicate with the PI, the site PIs and all study nurses regarding starting the study at all sites, data transfer, study-related enrollment and logistic issues, facilitate the REB-related matters as well as oversee the budget and organize the study log in Toronto.

2. Research nurses or respiratory therapists at all participating sites will be responsible for screening, enrollment and study execution and electronic data entry as well as the data transfer.

Sample Size:

The sample size calculation is based on the assessment of the between-group difference in proportions of hospitalizations. The estimated hospitalization rate is based on our pilot data where the *overall* (*control plus intervention*) hospitalization rate is 40%. Since this ongoing pilot remains blinded, it is certainly feasible that the *control group hospitalization rate* may be as high as 50%. This admission rate is greater than that in a 2006 prospective audit of 1000 children presenting with acute asthma at Canadian EDs which showed that approximately 30% of patients with a PRAM score of ≥ 5 after bronchodilator therapy were hospitalized (Appendix B).While the admission rate in our current study is substantially higher than in the previous audit, the one study in adults that focused on non-respondents to optimized initial Rx had an even higher admission rate of 71%.[63] *In order to ensure adequate power,we have conservatively used the hospitalization rate from our pilot* as compared to lower estimates using historical data. This is a superiority study in which the adoption of the Mg therapy can only be recommended for future practice if the rate of the primary outcome in this group is significantly lower than in the controls. With 408 patients per arm (816 in total) a two-sided test with a type I error of 0.05 will have 80% power to achieve statistical significance if Mg therapy reduces the probability of hospitalization to 40% (i.e. absolute reduction of 10%) [104] . This estimate is based on clinically relevant differences agreed upon by all study authors and it also represents NNT of 10. In the Cochrane reviews of anticholinergics and early corticosteroids by Plotnick and Rowe, respective NNTs of 12 and 8 led to a change in national practice recommendations.[105,106] In our North America-wide survey the majority of respondents considered a 10% reduced risk as a minimally clinically important difference that would prompt adoption of Mg.[54] Since almost a half of pediatric asthma costs relates to hospitalizations, this target difference would also have significant economic impact. Since our pilot has already enrolled 124 patients, 692 additional subjects need to be recruited. Based on the current study, the anticipated refusal rate will be 24%. Although the study non-completion rate and loss to follow-up are both currently 0%, we assume that each may be as high as 5%. Therefore, to have complete data on 692 patients we plan to randomize 766 (*i.e.* 692/(1 - 0.05)*(1 – 0.05) and to approach 1008 (*i.e.* 766/(1 - 0.24).

.

Statistical Analysis:

The primary analysis:

A two-sided Fisher's exact test will be used to test the null hypothesis that the treatment arms are equal with respect to the probability of hospitalization. This analysis will be performed on all randomized patients, according to the intent-to-treat principle, using a two-sided test of hypothesis with a type I error of 0.05. A nominal level of 4% for the type I error rate will be used to account for the interim analysis.

The secondary analyses:
a) Repeated measures ANOVA to compare treatment arms with respect to the changes in the PRAM score, respiratory rate, heart rate, oxygen saturation, and blood pressure over time.
b) A Poisson model will be used to compare the number of salbutamol treatments used in the ED in the two study arms.
c) Logistic regression analysis, including interaction terms with treatment group, will be used to examine the subgroup effects with respect to the primary outcome. The following variables will be used to define subgroups: age, gender, pre-randomization PRAM score, personal history of atopy.
The statistical tests of hypotheses for the secondary outcomes a) through c will two-sided at the 0.017 level to account for the issue of multiple testing and to maintain an overall type 1 error rate of 0.05.

Interim Analysis:
To assure safety, there will be one planned interim analysis on the first 200 patients randomized (a quarter through the study) conducted by a statistician not involved in the trial and evaluated by the independent data safety monitoring board. The interim analysis will be a one-sided test of the null hypothesis of no difference versus the alternative hypothesis that the probability of hospitalization is higher on Mg therapy at the 0.01 level. That is, we are looking for evidence that Mg therapy is less effective, and the trial will be stopped at an interim analysis only if the null hypothesis is rejected in favor of the control arm. Therefore, the interim analysis is only for safety and not for efficacy and it will not increase the probability of erroneously rejecting the null hypothesis in favor of Mg therapy at the final analysis. The reason we are doing one-sided (for harm) interim analysis is because if there is early strong evidence that Mg increases the probability of hospital admission, we want to stop the trial. On the other hand, we do not want to stop the trial early for benefit because a smaller sample size will not be convincing.

Feasibility:
We plan to implement an enrollment schedule similar to the one used in the current study, for a total of 88 hours a week. Extensive weekly coverage is needed since the time of presentation of these children varies. These hours will be covered by a combination of clinical research nurse coordinators and several trained on call research nurses. Based on the current pilot study, approximately 1337 patients ≥2 years of age present to SickKids and Alberta Children's annually, 87 of which were enrolled and completed the study in **one asthma season** (**Appendix E**). Based on the current study logs, we anticipate a 7% miss rate, 17% eligibility, 24% refusal rate, 5% may not finish the full experimental Rx (0% to date) and 5% may be lost to follow up (0% to date). Based on these enrollment rates and annual asthma presentations to the participating EDs, 3994 children can be expected to present to the participating EDs, of which 2006 (50%) will be screened, 342 will be eligible and 260 are projected to be

randomized. Although 100% patients enrolled to date have completed experimental Rx and both the 24 hour and 7 day follow up, up to 5% may not fulfill either requirement, leaving 234 patients with full data per asthma season (**Appendix E**). Therefore, 3.2 asthma seasons (31 study months) which will include 4 fall periods when asthma presentations are the most plentiful represent a reasonable timeline for obtaining the required sample size. Since *virtually all asthma cases occur between September and May, these totals represent one "asthma season".* To save money, enrollment will be limited to these periods. ***TIMELINE:*** **Oct'13-Apr'14:** regulatory documents, investigator meetings, REB, distribution of nebulizers, **Jan-Apr 14:** hiring of personnel, **May-Aug '14:** personnel training, **Sept '14-May '15:** 1st year recruitment, **Sept '15- May '16**: 2nd year recruitment, **Sept'16-May'17** 3rd year of recruitment**, Sept-Dec '17**- last recruitment, **Jan '18–Mar18**: Data management, analysis, **Apr- June '18**: Abstract and manuscript preparation.
Compliance with the experimental therapy is expected to be excellent since the nurses will administer and supervise its delivery in all children and the entire intervention will take place in the ED. They will also ensure the nebulizer mask stays on the face throughout treatment. We have conducted numerous past studies with successful nebulized bronchodilator delivery with a mask-face seal facilitated by the research nurse.[108-119] The experimental period is very short which will also enhance compliance. In our extensive experience, virtually no patients fail to finish experimental therapy. We have adjusted the sample size by 5% to account for /loss to follow-up.

Adverse Events

Magnesium blocks the neuromuscular transmission and acts as a CNS depressant. Therefore, the theoretical adverse effects with IV Mg may include a transient drop in blood pressure, apnea and heart block.[61] None of the IV or inhaled Mg trials has reported any of these issues and none of these have occurred during the pilot phase of the study. One study detected burning at the IV site, flushing and fatigue.[120] In their systematic review, Rowe et al. reported a clinically non- significant decrease in blood pressure.[121] However, hypotension related to IV Mg does occur, as documented in our North-America-wide survey. None of the surveyed physicians have witnessed heart block related to IV Mg and ‹1% have witnessed apnea. The potential for these problems after nebulized Mg is much lower than with IV Mg since this treatment route will result in a lower systemic delivery of Mg (1/4 of the IV dose) and a lower systemic effect. Of note, a recent Cochrane review of 896 patients given inhaled Mg confirmed the safety of this agent.[73] No child in the current inhaled Mg study had experienced hypotension or other side effects.

All adverse events will be reported to the Hospital for Sick Children Research Ethics Board according to the Hospital for Sick Children's adverse event reporting requirements. Adverse events will be classified as mild, moderate or severe and as expected or unexpected. Expected adverse events will include cough, respiratory distress (disease-related), asthma-related hospitalization, IV insertion, sinus tachycardia and bitter/salty taste of the experimental solution. All serious, unexpected adverse drug reactions to the study medication will be reported to Health Canada within 15 calendar days or for death or life-threatening events,

within 7 calendar days. In the latter case, a follow-up report must be filed within 8 calendar days. Adverse reactions will be managed according to the Hospital for Sick Children's standard clinical management practices. Furthermore, we plan to document episodes of severe cough necessitating interruption of the experimental therapy for more than approximately 3 minutes to examine the safety profile of magnesium.

Since hypotension is the only major side-effect of IV Mg occurring with appreciable frequency, all enrolled patients will be on precautionary frequent blood pressure monitoring as per the study protocol. If the systolic blood pressure drops below 5th percentile for age, the study will be stopped, treatment given as necessary and DSMC will be notified. This has not happened during the current pilot phase of the study.

Due to the osmolarity of the study solutions being well under 500 mOsm/L throughout nebulization and co-administration of salbutamol, we do not anticipate side effects to occur as a result of using the aforementioned composition of the study solutions. However, should the highly unlikely event of respiratory deterioration occur, the experimental therapy will be discontinued, appropriate additional treatment started and the event will be reported to the DSMC within 48 hours. Salbutamol may cause tachycardia and this was also the case in many children enrolled to date. However, this was uniformly well tolerated and no patient had to stop/interrupt experimental therapy due to this issue.

To ensure safety of the participating subjects, unstable children requiring immediate airway stabilization will be excluded. We are also planning an interim analysis to maximize safety.

<u>**Data Safety and Monitoring Committee (DSMC):**</u>

The Data Safety and Monitoring Committee (DSMC) will consist of a non-study biostatistician, an ED physician and researcher and an ED scientist. The members of this committee will not be collaborators of this trial. They will be notified of all serious adverse events (such as hypotension <5th percentile for age, apnea, heart block, severe increase in respiratory distress necessitating discontinuation of the study) and of an admission to the ICU within 48 hours. Should any of these adverse events occur, they will be immediately reported from both sites to the study coordinator at SickKids who will promptly notify the DSMC. The DSMC will meet once per asthma season or ad hoc if necessary.

Dissemination of Results and Future Directions: The results of this study will be submitted for presentation at either the annual meeting of the Pediatric Academic Societies, the Society for Academic Emergency Medicine or the American Academy of Pediatrics. We shall also submit the manuscript for publication in a peer-reviewed scientific journal.

Limitations: In this study, we anticipate a very low rate of magnesium-related side effects such as hypotension. The major reason for this is a limited systemic magnesium delivery, which will

be much lower than with the IV therapy. However, the study sample size will not permit us to conduct a meaningful statistical analysis of magnesium-related adverse events since we anticipate an extremely small number of such events, if any.

Literature References

1. Mannino DM, Homa DM, Pertowski CA, et al. Surveillance for asthma--United States, 1960-1995. Morbidity & Mortality Weekly Report CDC Surveillance Summaries 1998;47:1-27.
2. Asthma prevalence, health care use and mortality: United States, 2003-2005. Centers for Disease Control and Prevention, November 2006. (Accessed October 1, 2009, at http://www.cdc.gov/nchs/data/hestat/ashtma03-05/asthma03-05.htm.)
3. Moorman JE, Rudd RA, Johnson CA, et al. National surveillance for asthma--United States, 1980-2004. Morbidity & Mortality Weekly Report Surveillance Summaries 2007;56:1-54.
4. Camargo CA, Investigators M. Acute asthma among children presenting to the emergency department: the Multicenter Asthma Research Collaboration. Acad Emerg Med 1998;5:380.
5. Scarfone RJ, Fuchs SM, Nager AL, Shane SA. Controlled trial of oral prednisone in the emergency department treatment of children with acute asthma.[see comment]. Pediatrics 1993;92:513-8.
6. Robertson CF, Smith F, Beck R, Levison H. Response to frequent low doses of nebulized salbutamol in acute asthma. J Pediatr 1985;106:672-4.
7. Schuh S, Parkin P, Rajan A, et al. High-versus low-dose, frequently administered, nebulized albuterol in children with severe, acute asthma. Pediatrics 1989;83:513-8.
8. Plotnick LH, Ducharme FM. Combined inhaled anticholinergic agents and beta-2-agonists for initial treatment of acute asthma in children.[update in Cochrane Database Syst Rev. 2000;(4):CD000060; PMID: 11034671]. Cochrane Database of Systematic Reviews 2000:CD000060.
9. Rowe BH, Edmonds ML, Spooner CH, Camargo CA. Evidence-based treatments for acute asthma. Respir Care 2001;46:1380-90.
10. National Heart, Lung and Blood Institute: Global Initiative for asthma. Global strategy for asthma management and prevention. NHLBI/WHO workshop report. Bethesda, Md: NIH; 2002. Report No.: 02-3659.
11. National Institutes of Health and National Heart, Lung, and Blood Institute. Guidelines for the Diagnosis and Management of Asthma - Update on Selected Topics 2002. Washington, DC: US Dept of Health and Human Services; 2002. Report No.: 97-4051.
12. National Asthma Council. Asthma Management Handbook 2002. Melbourne: National Asthma Council Australia, Ltd; 2002.
13. British Thoracic S, Scottish Intercollegiate Guidelines N. British guideline on the management of asthma. Thorax 2003;58 Suppl 1:i1-94.
14. Becker A, Lemiere C, Berube D, et al. Summary of recommendations from the Canadian Asthma Consensus guidelines, 2003. CMAJ Canadian Medical Association Journal 2005;173:S3-11.
15. National Asthma Education and Prevention Program. Expert Panel Report 3: guidelines for the diagnosis and management of asthma. Bethesda, MD: National Institutes of Health, National Heart, Lung, and Blood Institute; 2007.
16. Hall IP. Pharmacogenetics of asthma. Eur Respir J 2000;15:449-51.
17. Drazen JM, Silverman EK, Lee TH. Heterogeneity of therapeutic responses in asthma. Br Med Bull 2000;56:1054-70.
18. Palmer LJ, Silverman ES, Weiss ST, Drazen JM. Pharmacogenetics of asthma. Am J Respir Crit Care Med 2002;165:861-6.
19. Koga T, Kamimura T, Oshita Y, et al. Determinants of bronchodilator responsiveness in patients with controlled asthma. J Asthma 2006;43:71-4.
20. Tsai HJ, Shaikh N, Kho JY, et al. Beta 2-adrenergic receptor polymorphisms: pharmacogenetic response to bronchodilator among African American asthmatics. Hum Genet 2006;119:547-57.

21. Johnson M. The beta-adrenoceptor. Am J Respir Crit Care Med 1998;158:S146-53.
22. Taylor DR, Epton MJ, Kennedy MA, et al. Bronchodilator response in relation to beta2-adrenoceptor haplotype in patients with asthma. Am J Respir Crit Care Med 2005;172:700-3.
23. Lipworth BJ, Hall IP, Tan S, Aziz I, Coutie W. Effects of genetic polymorphism on ex vivo and in vivo function of beta2-adrenoceptors in asthmatic patients. Chest 1999;115:324-8.
24. Israel E, Drazen JM, Liggett SB, et al. The effect of polymorphisms of the beta(2)-adrenergic receptor on the response to regular use of albuterol in asthma. Am J Respir Crit Care Med 2000;162:75-80.
25. Taylor DR, Drazen JM, Herbison GP, Yandava CN, Hancox RJ, Town GI. Asthma exacerbations during long term beta agonist use: influence of beta(2) adrenoceptor polymorphism. Thorax 2000;55:762-7.
26. Israel E, Chinchilli VM, Ford JG, et al. Use of regularly scheduled albuterol treatment in asthma: genotype-stratified, randomised, placebo-controlled cross-over trial.[see comment]. Lancet 2004;364:1505-12.
27. Palmer CN, Lipworth BJ, Lee S, Ismail T, Macgregor DF, Mukhopadhyay S. Arginine-16 beta2 adrenoceptor genotype predisposes to exacerbations in young asthmatics taking regular salmeterol.[see comment]. Thorax 2006;61:940-4.
28. Contopoulos-Ioannidis DG, Manoli EN, Ioannidis JP. Meta-analysis of the association of beta2-adrenergic receptor polymorphisms with asthma phenotypes.[see comment]. J Allergy Clin Immunol 2005;115:963-72.
29. Martinez FD, Graves PE, Baldini M, Solomon S, Erickson R. Association between genetic polymorphisms of the beta2-adrenoceptor and response to albuterol in children with and without a history of wheezing. J Clin Invest 1997;100:3184-8.
30. Carroll CL, Schramm CM, Zucker AR. Slow-responders to IV beta2-adrenergic agonist therapy: defining a novel phenotype in pediatric asthma. Pediatr Pulmonol 2008;43:627-33.
31. Liggett SB. Polymorphisms of the beta2-adrenergic receptor and asthma. Am J Respir Crit Care Med 1997;156:S156-62.
32. Thakkinstian A, McEvoy M, Minelli C, et al. Systematic review and meta-analysis of the association between {beta}2-adrenoceptor polymorphisms and asthma: a HuGE review. Am J Epidemiol 2005;162:201-11.
33. Choudhry S, Ung N, Avila PC, et al. Pharmacogenetic differences in response to albuterol between Puerto Ricans and Mexicans with asthma.[see comment]. Am J Respir Crit Care Med 2005;171:563-70.
34. Martin AC, Zhang G, Rueter K, et al. Beta2-adrenoceptor polymorphisms predict response to beta2-agonists in children with acute asthma. J Asthma 2008;45:383-8.
35. Kercsmar CM, McDowell KM. Love it or lev it: levalbuterol for severe acute asthma--for now, leave it.[comment]. J Pediatr 2009;155:162-4.
36. Weiss KB, Sullivan SD, Lyttle CS. Trends in the cost of illness for asthma in the United States, 1985-1994. J Allergy Clin Immunol 2000;106:493-9.
37. Ducharme FM, Chalut D, Plotnick L, et al. The Pediatric Respiratory Assessment Measure: a valid clinical score for assessing acute asthma severity from toddlers to teenagers. J Pediatr 2008;152:476-80.
38. Ungar WJ, Coyte PC, Pharmacy Medication Monitoring Program Advisory B. Prospective study of the patient-level cost of asthma care in children. Pediatr Pulmonol 2001;32:101-8.
39. Parameswaran K, Belda J, Rowe BH. Addition of intravenous aminophylline to beta2-agonists in adults with acute asthma. Cochrane Database of Systematic Reviews 2000:CD002742.
40. Littenberg B. Aminophylline treatment in severe, acute asthma. A meta-analysis. JAMA 1988;259:1678-84.
41. Browne GJ, Lam LT. Single-dose intravenous salbutamol bolus for managing children with acute severe asthma in the emergency department:Reanalysis of data. Pediatr Crit Care Med 2002;3:117-23.

42. Browne GJ, Penna AS, Phung X, Soo M. Randomised trial of intravenous salbutamol in early management of acute severe asthma in children. Lancet 1997;349:301-5.
43. Browne GJ, Trieu L, Van Asperen P. Randomized, double-blind, placebo-controlled trial of intravenous salbutamol and nebulized ipratropium bromide in early management of severe acute asthma in children presenting to an emergency department. Crit Care Med 2002;30:448-53.
44. Spivey WH, Skobeloff EM, Levin RM. Effect of magnesium chloride on rabbit bronchial smooth muscle. Ann Emerg Med 1990;19:1107-12.
45. Iseri LT, French JH. Magnesium: nature's physiologic calcium blocker. Am Heart J 1984;108:188-93.
46. Hill J, Britton J. Dose-response relationship and time-course of the effect of inhaled magnesium sulphate on airflow in normal and asthmatic subjects. Br J Clin Pharmacol 1995;40:539-44.
47. Rolla G, Bucca C, Brussino L, Colagrande P. Effect of intravenous magnesium infusion on salbutamol-induced bronchodilatation in patients with asthma. Magnes Res 1994;7:129-33.
48. Cairns CB, Kraft M. Magnesium attenuates the neutrophil respiratory burst in adult asthmatic patients.[see comment]. Acad Emerg Med 1996;3:1093-7.
49. Mohammed S, Goodacre S. Intravenous and nebulised magnesium sulphate for acute asthma: systematic review and meta-analysis. Emerg Med J 2007;24:823-30.
50. Cheuk DK, Chau TC, Lee SL. A meta-analysis on intravenous magnesium sulphate for treating acute asthma. Arch Dis Child 2005;90:74-7.
51. Global Initiative for Asthma. (Accessed October 5, 2009, at www.ginasthma.com.)
52. Canadian Asthma Consensus Group. Management of patients with asthma in the emergency department and in hospital. CMAJ Canadian Medical Association Journal 1999;161:S53-9.
53. British guideline on the management of asthma. 2009. (Accessed October 23, 2009, at http://www.brit-thoracic.org.uk/Portals/0/Clinical%20Information/Asthma/Guidelines/sign101%20revised%20June%2009.pdf.)
54. Schuh S, Macias C, Freedman S, et al. North American Practice Patterns of IV Magnesium Therapy in Severe Acute Asthma in Children. Acad Emerg Med 2010;17:1189-96.
55. Taddio A, Soin HK, Schuh S, Koren G, Scolnik D. Liposomal lidocaine to improve procedural success rates and reduce procedural pain among children: a randomized controlled trial.[see comment]. CMAJ Canadian Medical Association Journal 2005;172:1691-5.
56. Black KJ, Pusic MV, Harmidy D, McGillivray D. Pediatric intravenous insertion in the emergency department: bevel up or bevel down? Pediatr Emerg Care 2005;21:707-11.
57. Arts SE, Abu-Saad HH, Champion GD, et al. Age-related response to lidocaine-prilocaine (EMLA) emulsion and effect of music distraction on the pain of intravenous cannulation. Pediatrics 1994;93:797-801.
58. Manner T, Kanto J, Iisalo E, Lindberg R, Viinamaki O, Scheinin M. Reduction of pain at venous cannulation in children with a eutectic mixture of lidocaine and prilocaine (EMLA cream): comparison with placebo cream and no local premedication. Acta Anaesthesiol Scand 1987;31:735-9.
59. Taddio A, Gurguis MG, Koren G. Lidocaine-prilocaine cream versus tetracaine gel for procedural pain in children. Ann Pharmacother 2002;36:687-92.
60. Walco GA, Burns JP, Cassidy RC. The ethics of pain control in infants and children. In: Schechter NL, Berde CB, Yaster M, eds. Pain in infants, children, and adolescents. 2nd ed. New York: Lippincott Williams & Wilkins; 2003:157-68.
61. Magnesium Sulphate: Adverse Effects. In: Reynolds JEF, Parfitt K, Parsons AV, Sweetman SC, Council of the Royal Pharmaceutical Society of Great Britain, Department of Pharmaceutical Sciences, eds. The extra pharmacopoeia. 29th ed. London: Pharmaceutical Press; 1989:1033.

62. Okayama H, Aikawa T, Okayama M, Sasaki H, Mue S, Takishima T. Bronchodilating effect of intravenous magnesium sulfate in bronchial asthma. JAMA 1987;257:1076-8.
63. Hughes R, Goldkorn A, Masoli M, Weatherall M, Burgess C, Beasley R. Use of isotonic nebulised magnesium sulphate as an adjuvant to salbutamol in treatment of severe asthma in adults: randomised placebo-controlled trial.[see comment]. Lancet 2003;361:2114-7.
64. Mahajan P, Haritos D, Rosenberg N, Thomas R. Comparison of nebulized magnesium sulfate plus albuterol to nebulized albuterol plus saline in children with acute exacerbations of mild to moderate asthma. J Emerg Med 2004;27:21-5.
65. Aggarwal P, Sharad S, Handa R, Dwiwedi SN, Irshad M. Comparison of nebulised magnesium sulphate and salbutamol combined with salbutamol alone in the treatment of acute bronchial asthma: a randomised study. Emergency Medicine Journal 2006;23:358-62.
66. Drobina BJ, Kostic MA, Roos JA. Nebulized magnesium has no benefit in the treatment of acute asthma in the emergency department. Acad Emerg Med 2006;13.
67. Kokturk N, Turktas H, Kara P, Mullaoglu S, Yilmaz F, Karamercan A. A randomized clinical trial of magnesium sulphate as a vehicle for nebulized salbutamol in the treatment of moderate to severe asthma attacks.[see comment]. Pulm Pharmacol Ther 2005;18:416-21.
68. Bessmertny O, DiGregorio RV, Cohen H, et al. A randomized clinical trial of nebulized magnesium sulfate in addition to albuterol in the treatment of acute mild-to-moderate asthma exacerbations in adults. Ann Emerg Med 2002;39:585-91.
69. Nannini LJ, Pendino JC, Corna RA, Mannarino S, Quispe R. Magnesium sulfate as a vehicle for nebulized salbutamol in acute asthma. The American journal of medicine 2000;108:193-7.
70. Powell C. A randomised, double blind, placebo controlled study of nebulised magnesium sulphate in asute asthma in children – The MAGNETIC study. Archives of Disease in Childhood 2012;97:A2-A3.
71. Blitz M, Blitz S, Hughes R, et al. Aerosolized magnesium sulfate for acute asthma: a systematic review.[see comment][erratum appears in Chest. 2005 Nov;128(5):3779]. Chest 2005;128:337-44.
72. Blitz M, Blitz S, Beasely R, et al. Inhaled magnesium sulfate in the treatment of acute asthma.[update in Cochrane Database Syst Rev. 2005;(4):CD003898; PMID: 16235345][update of Cochrane Database Syst Rev. 2005;(2):CD003898; PMID: 15846687]. Cochrane Database of Systematic Reviews 2005:CD003898.
73. Powell C, Dwan K, Milan SJ, et al. Inhaled magnesium sulfate in the treatment of acute asthma. The Cochrane database of systematic reviews 2012;12:CD003898.
74. Coates A, Leung K, Vecellio None L, Schuh S. The Choice of a Nebulizer for Delivering Magnesium Sulfate to Pediatric Asthmatic Patients in the Emergency Department. Respir Care In Press.
75. Coates AL, MacNeish CF, Allen PD, Ho SL, Lands LC. Do sinusoidal models of respiration accurately reflect the respiratory events of patients breathing on nebulizers? J Aerosol Med 1999;12:265-73.
76. Katz SL, Adatia I, Louca E, et al. Nebulized therapies for childhood pulmonary hypertension: an in vitro model. Pediatr Pulmonol 2006;41:666-73.
77. Coates AL, Dinh L, MacNeish CF, et al. Accounting for radioactivity before and after nebulization of tobramycin to insure accuracy of quantification of lung deposition. J Aerosol Med 2000;13:169-78.
78. Coates AL, Tipples G, Leung K, Gray M, Louca E, Vaccine WHOPDGfMA. How many infective viral particles are necessary for successful mass measles immunization by aerosol? Vaccine 2006;24:1578-85.
79. Mitchell JP, Nagel MW, Nichols S, Nerbrink O. Laser diffractometry as a technique for the rapid assessment of aerosol particle size from inhalers. J Aerosol Med 2006;19:409-33.
80. Coates AL, Macneish CF, Lands LC, et al. Factors influencing the rate of drug output during the course of wet nebulization. Journal of Aerosol Medicine-Deposition Clearance and Effects in the Lung 1998;11:101-11.

81. Dodd ME, Abbott J, Maddison J, Moorcroft AJ, Webb AK. Effect of tonicity of nebulised colistin on chest tightness and pulmonary function in adults with cystic fibrosis. Thorax 1997;52:656-8.
82. Ciarallo L, Brousseau D, Reinert S. Higher-dose intravenous magnesium therapy for children with moderate to severe acute asthma. Arch Pediatr Adolesc Med 2000;154:979-83.
83. Chalut DS, Ducharme FM, Davis GM. The Preschool Respiratory Assessment Measure (PRAM): a responsive index of acute asthma severity.[see comment]. J Pediatr 2000;137:762-8.
84. Chua HL, Collis GG, Newbury AM, et al. The influence of age on aerosol deposition in children with cystic fibrosis. Eur Respir J 1994;7:2185-91.
85. Newhouse MT, Dolovich MB. Control of asthma by aerosols.[see comment]. N Engl J Med 1986;315:870-4.
86. Tal A, Golan H, Grauer N, Aviram M, Albin D, Quastel MR. Deposition pattern of radiolabeled salbutamol inhaled from a metered-dose inhaler by means of a spacer with mask in young children with airway obstruction. J Pediatr 1996;128:479-84.
87. Wildhaber JH, Dore ND, Wilson JM, Devadason SG, LeSouef PN. Inhalation therapy in asthma: nebulizer or pressurized metered-dose inhaler with holding chamber? In vivo comparison of lung deposition in children.[see comment]. J Pediatr 1999;135:28-33.
88. Leung K, Louca E, Coates AL. Comparison of breath-enhanced to breath-actuated nebulizers for rate, consistency, and efficiency. Chest 2004;126:1619-27.
89. Wildhaber JH, Devadason SG, Hayden MJ, Eber E, Summers QA, LeSouef PN. Aerosol delivery to wheezy infants: a comparison between a nebulizer and two small volume spacers. Pediatr Pulmonol 1997;23:212-6.
90. Qureshi F, Pestian J, Davis P, Zaritsky A. Effect of nebulized ipratropium on the hospitalization rates of children with asthma. N Engl J Med 1998;339:1030-5.
91. Kopach R, Sadat S, Gallaway ID, Geiger G, Ungar WJ, Coyte PC. Cost-effectiveness analysis of medical documentation alternatives. Int J Technol Assess Health Care 2005;21:126-31.
92. Brand PL, Baraldi E, Bisgaard H, et al. Definition, assessment and treatment of wheezing disorders in preschool children: an evidence-based approach. Eur Respir J 2008;32:1096-110.
93. Ducharme FM, Lemire C, Noya FJ, et al. Preemptive use of high-dose fluticasone for virus-induced wheezing in young children.[see comment]. N Engl J Med 2009;360:339-53.
94. Oommen A, Lambert PC, Grigg J. Efficacy of a short course of parent-initiated oral prednisolone for viral wheeze in children aged 1-5 years: randomised controlled trial.[see comment]. Lancet 2003;362:1433-8.
95. Vuillermin PJ, Robertson CF, South M. Parent-initiated oral corticosteroid therapy for intermittent wheezing illnesses in children: systematic review.[see comment]. J Paediatr Child Health 2007;43:438-42.
96. Johnston SL, Pattemore PK, Sanderson G, et al. Community study of role of viral infections in exacerbations of asthma in 9-11 year old children.[see comment]. BMJ 1995;310:1225-9.
97. Martinez FD, Wright AL, Taussig LM, Holberg CJ, Halonen M, Morgan WJ. Asthma and wheezing in the first six years of life. The Group Health Medical Associates.[see comment]. N Engl J Med 1995;332:133-8.
98. Castro-Rodriguez JA, Holberg CJ, Wright AL, Martinez FD. A clinical index to define risk of asthma in young children with recurrent wheezing. Am J Respir Crit Care Med 2000;162:1403-6.
99. Birken CS, Parkin PC, Macarthur C. Asthma severity scores for preschoolers displayed weaknesses in reliability, validity, and responsiveness. J Clin Epidemiol 2004;57:1177-81.
100. Panickar J, Lakhanpaul M, Lambert PC, et al. Oral prednisolone for preschool children with acute virus-induced wheezing. N Engl J Med 2009;360:329-38.
101. Ducharme FM, Davis GM. Respiratory resistance in the emergency department: a reproducible and responsive measure of asthma severity.[see comment]. Chest 1998;113:1566-72.

102. Gorelick MH, Stevens MW, Schultz TR, Scribano PV. Performance of a novel clinical score, the Pediatric Asthma Severity Score (PASS), in the evaluation of acute asthma. Acad Emerg Med 2004;11:10-8.
103. Akinbami LJ, Schoendorf KC. Trends in childhood asthma: prevalence, health care utilization, and mortality. Pediatrics 2002;110:315-22.
104. Fleiss JL. Statistical Methods for Rates and Proportions. Second Edition: Wiley, John and Sons, Incorporated, New York, N.Y.; 1981.
105. Plotnick LH, Ducharme FM. Combined inhaled anticholinergics and beta2-agonists for initial treatment of acute asthma in children. The Cochrane database of systematic reviews 2000:CD000060.
106. Rowe BH, Spooner C, Ducharme FM, Bretzlaff JA, Bota GW. Early emergency department treatment of acute asthma with systemic corticosteroids. The Cochrane database of systematic reviews 2001:CD002178.
107. Fleiss JL. Statistical methods for rates and proportions. 2nd ed. New York, NY: Wiley; 1981.
108. Schuh S, Canny G, Reisman JJ, et al. Nebulized albuterol in acute bronchiolitis. J Pediatr 1990;117:633-7.
109. Schuh S, Reider MJ, Canny G, et al. Nebulized albuterol in acute childhood asthma: comparison of two doses. Pediatrics 1990;86:509-13.
110. Bentur L, Canny GJ, Shields MD, et al. Controlled trial of nebulized albuterol in children younger than 2 years of age with acute asthma. Pediatrics 1992;89:133-7.
111. Schuh S, Johnson D, Canny G, et al. Efficacy of adding nebulized ipratropium bromide to nebulized albuterol therapy in acute bronchiolitis. Pediatrics 1992;90:920-3.
112. Schuh S, Johnson DW, Callahan S, Canny G, Levison H. Efficacy of frequent nebulized ipratropium bromide added to frequent high-dose albuterol therapy in severe childhood asthma. J Pediatr 1995;126:639-45.
113. Johnson DW, Schuh S, Koren G, Jaffee DM. Outpatient treatment of croup with nebulized dexamethasone. Arch Pediatr Adolesc Med 1996;150:349-55.
114. Johnson DW, Jacobson S, Edney PC, Hadfield P, Mundy ME, Schuh S. A comparison of nebulized budesonide, intramuscular dexamethasone, and placebo for moderately severe croup. N Engl J Med 1998;339:498-503.
115. Schuh S, Johnson DW, Stephens D, Callahan S, Winders P, Canny GJ. Comparison of albuterol delivered by a metered dose inhaler with spacer versus a nebulizer in children with mild acute asthma. J Pediatr 1999;135:22-7.
116. Schuh S, Reisman J, Alshehri M, et al. A comparison of inhaled fluticasone and oral prednisone for children with severe acute asthma. N Engl J Med 2000;343:689-94.
117. Schuh S, Coates AL, Binnie R, et al. Efficacy of oral dexamethasone in outpatients with acute bronchiolitis. J Pediatr 2002;140:27-32.
118. Scolnik D, Coates AL, Stephens D, Da Silva Z, Lavine E, Schuh S. Controlled delivery of high vs low humidity vs mist therapy for croup in emergency departments: a randomized controlled trial. JAMA 2006;295:1274-80.
119. Schuh S, Dick PT, Stephens D, et al. High-dose inhaled fluticasone does not replace oral prednisolone in children with mild to moderate acute asthma. Pediatrics 2006;118:644-50.
120. Bloch H, Silverman R, Mancherje N, Grant S, Jagminas L, Scharf SM. Intravenous magnesium sulfate as an adjunct in the treatment of acute asthma. Chest 1995;107:1576-81.
121. Rowe BH, Bretzlaff JA, Bourdon C, Bota GW, Camargo CA, Jr. Intravenous magnesium sulfate treatment for acute asthma in the emergency department: a systematic review of the literature. Annals of emergency medicine 2000;36:181-90.

Appendix A: Pediatric Respiratory Assessment Measure (PRAM) Score

Signs	0	1	2	3
Suprasternal retractions	Absent		Present	
Scalene muscle contraction	Absent		Present	
Air entry*	Normal	Decreased at bases	Widespread decrease	Absent/minimal
Wheezing*	Absent	Expiratory only	Inspiratory and expiratory	Audible without stethoscope/ silent chest with minimal air entry
O2 saturation	≥95% - Toronto<br>≥93% - Calgary	92%-94% - Toronto<br>90%-92% - Calgary	<92% - Toronto<br>< 90% - Calgary	

*If asymmetric findings between the right and left lungs, the most severe side is rated.
Reprinted from The Journal of Pediatrics, Vol 137, Issue 6, Chalut DS, Ducharme FM, Davis, GM. The Preschool Assessment Measure (PRAM): A responsive index of acute asthma severity. Pages 762-768, Copyright © 2000 with permission from Elsevier.

Appendix B: **<u>Pediatric Respiratory Assessment Measure (PRAM) Scores in Triage and After Initial Bronchodilator Therapy*</u>**

**Triage PRAM**: (N)		**Post-Bronchodilator Therapy PRAM ≥ 5**
4:	74	16 (22%)
5:	69	24 (35%)
6:	88	45 (51%)
7:	50	34 (68%)
8:	32	25 (78%)
9:	18	15 (83%)
10:	10	8 (80%)
11:	11	11 (100%)

Of children with PRAM ≥5 in triage, 58% (162/278) have post-bronchodilator therapy PRAM of ≥ 5.

<u>Probability of Hospitalization with different post-bronchodilator therapy PRAM scores*</u>

PRAM ≥ 4:	61/290 = 21%
PRAM ≥ 5:	53/184 = 30%
PRAM ≥ 6:	45/113 = 40%

<u>Post-Bronchodilator PRAM score as a Proportion of Asthma Hospitalizations*</u>

PRAM ≥ 4:	97%
PRAM ≥ 5:	84%
PRAM ≥ 6:	71%
PRAM ≥ 7:	49%

*2006 Asthma Audit from a Canadian pediatric ED

Appendix C: LOGISTICS OF BLINDING AND KIT MAKING

	**Investigational Drug or Placebo (mg=mL) (provided in a blinded vial)**	**Salbutamol Nebulizer Solution 5mg/mL (mg=mL)**	**Diluent Volume to Top up to 6mL Final Volume (mL)**	Osmolarity (mOsm/L)
Active Arm	Magnesium Sulfate Injection 500mg/mL (600mg Mg Sulf = 1.2mL)	5mg = 1mL	Sterile Water for Injection (3.8mL)	**384**
Placebo Arm	Hypertonic Saline (5.5%) (0mg Mg Sulf = 1.2mL)	5mg = 1mL	Sterile Water for Injection (3.8mL)	**381**

Each site will prepare consecutively numbered randomization kits, numbered according to the site's Master Randomization table. Each kit will contain:
Magnesium Sulfate Injection 500mg/mL OR **Hypertonic Saline** (5.5%)

Active kits will contain Magnesium Sulfate injection
- Injection to be administered by nebulized inhalation
- Unblinded site pharmacy will repackage small batches of Canadian commercial Magnesium injection into empty sterile vials in a laminar air flow hood according to detailed worksheet procedures in the Pharmacy Manual of Operations.

Placebo Kits will contain Hypertopic Saline 5.5%
- Unblinded site pharmacy will compound small batches of Hypertonic Saline (5.5%) in a Laminar Air Flow hood using 14.6% concentrated Sodium Chloride and sterile water according to detailed worksheet procedures in the Pharmacy Manual of Operations.
- ***Hypertonic Saline (5.5%) was chosen as the Placebo since Magnesium Sulfate is hypertonic. 5.5% is the percentage that mimicks the osmolality of the Active arm when sterile water is used as the top up diluent.***

The repackaged Magnesium Sulfate and compounded placebo vials will be given a 6 month expiry date.
During Kit assembly by the site pharmacy, identical labels will be placed on the blinded vials in order to ensure the integrity of the blind.

Blinded Numbered Randomization Kits will be assembled by the unblinded site pharmacy and made available to the Emergency Study RNs for use once a subject is eligible to be randomized.

Open Label supplies of the following will be available:
1. Salbutamol Nebulizer Solution 5mg/mL Canadian commercial supply. No blinding required. Drug accountability according to Health Canada Division 5 regulations will be maintained.

2. Sterile Water for Injection (SWI)
Used as the diluent to top up to final 6mL nebulizer volume
Canadian commercial supply. No blinding required.
Drug accountability according to Health Canada Division 5 regulations will be maintained
Sterile Water was chosen as the top up diluent to ensure that <u>the final osmolality of the nebulizer solutions was less than 500</u> (the osmolality at which bronchospasm has been reported). <u>The inhalation solutions in both study arms will be of comparable isotonicity.</u>

In this Investigator initiated study, the numbered kits will be assembled and labeled in the local Research Pharmacy according to detailed kit making Standard Operating Procedures provided by the Coordinating Pharmacy at SickKids. All kits/products will have appropriate Clinical Trial labeling according to Canadian regulations.

Appendix D: EMERGENCY UNBLINDING PROCEDURES

In the unlikely event the patient develops hypotension requiring therapy, apnea, heart block or another adverse event and the ED physician feels that the experimental therapy cannot be safely continued, further doses of the experimental treatment will be stopped.

If these adverse events are accompanied by severe distress and additional IV Mg is warranted, the study may be unblinded for that subject. If the subject was allocated to the Active Mg Sulfate arm, then additional IV Mg should not be given but alternative treatment provided instead. If the subject was allocated to the Placebo arm, then IV Mg may be given as part of treatment of the adverse event.

Emergency unblinding should only be requested when the clinical treatment of the patient will be different by knowing which arm of the study the patient was previously on. The study PI/local PI and the study nurses will remain blinded if possible.

The following Emergency Unblinding procedure will be followed:

1. Treating Physician or RN should contact the local PI of the study for consultation to unblind. In the event they cannot be reached immediately go to the next step.
2. Contact the SickKids hospital pharmacy by phone.
3. Provide the patient’s study randomization number, reason for unblinding, your site and your name to the SickKids pharmacist who will then provide the unblinded study arm.
4. Note that all patients whose therapy is unblinded must stop taking the experimental therapy The ED physician will prescribe additional treatment as clinically appropriate.
5. The requesting physician should initiate Email communication within 24 hours detailing the request for Emergency unblinding and why. The email must inform the local PI and SickKids Research Pharmacist and Study PI.
6. The local DSMC and REB will be advised of emergency unblinding within 48 hours.

Appendix E: ANNUAL ENROLLMENT PROJECTION

**Site**	**Annual Asthma Presentations ≥2 years of age**	**Projected Annual Screens+**	**Projected Randomizations***	**Projected Annual Study Completion based on progress to date and asthma presentations**
Hospital for Sick Children	682	340	44	40
Children Hospital of Eastern Ontario	672	336	43	39
Alberta Children's Hospital	660	330	42	38
Stollery Children's Hospital	320	160	20	18
Winnipeg Children's Hospital	500	250	32	29
CHU – Sainte-Justine	670	335	43	39
Children's Hospital London	490	245	31	28
Total	**3994**	**1996**	**255**	**231**

+ screens represent approximately 50% of annual presentations as per current study
***** randomizations represent 13% of patients screened as per progress in current study

Current Seasonal Patient Accrual and Progress to date

Annual presentations at SickKids and ACH: 1337

↓

Available for screening: 718 (54%)

↓

Misses: 46 (6.5%)

↓

Screened: 672

↓

Exclusions: 558

↓

Eligible: 114 (17%)

↓

Refusals: 27 (24%)

↓

Randomized: 87

↓

Completed experimental Rx: 87 (100%)

↓

Follow up completed: 87 (100%)

Not screened (RNs off duty): 619

Exclusions:

PRAM <5 in triage/after Rx: 324

First wheeze: 68

Pneumonia 14

Co-morbidities:98

Transferred on IV Mg: 14

Allergy to Mg: 1

No English: 3

Previous enrollment: 6

Other reasons: 30

Magnesium Nebulization Utilization in Management of Pediatric Asthma – "MagNUM PA"

Expected annual patient accrual based on asthma presentations to participating EDs and study progress to date

Annual presentations: 3994 (September through May)

↓

Available for screening (54%): 2156

↓

Missed (7%): 150

↓

Screened: 2006

↓

Exclusions: 1664

↓

Eligible: 342 (17%)

↓

Refusals: 82 (24%)

↓

Randomized: 260

↓

Complete experimental Rx: 246 (95%)

↓

Complete follow up: 234 (95%)

Magnesium Nebulization Utilization in Management of Pediatric Asthma – "MagNUM PA"

Principal Investigator:

Dr. Suzanne Schuh: The Hospital for Sick Children, Toronto, Ontario

Co-Investigators:

Dr. David Johnson: Alberta Children's Hospital, Calgary, Alberta

Dr. Roger Zemek: Children's Hospital of Eastern Ontario (CHEO), Ottawa, Ontario

Dr. Stephen Freedman: Alberta Children's Hospital, Calgary, Alberta

Dr. Darcy Beer: Winnipeg Children's Hospital, Winnipeg, Manitoba

Dr. Amy Plint: Children's Hospital of Eastern Ontario (CHEO), Ottawa, Ontario

Dr. Karen Black: BC Children's Hospital, Vancouver, British Columbia

Dr. Andrew Willan: The Hospital for Sick Children, Toronto, Ontario

Dr. Francine Ducharme: CHU – Sainte-Justine Pédiatrie, Montreal, Quebec

Dr. Graham Thompson: Alberta Children's Hospital, Calgary, Alberta

Dr. Allan Coates: The Hospital for Sick Children, Toronto, Ontario

Dr. Jocelyn Gravel: CHU – Sainte-Justine Pédiatrie, Montreal, Quebec

Dr. Sarah Curtis: Edmonton Clinic Health Academy, University of Alberta

Collaborators:

Darcy Nicksy: The Hospital for Sick Children, Toronto, Ontario

Funding: Canadian Institutes for Health Research (CIHR), Thrasher Foundation, Physicians' Services Incorporated Foundation, SickKids Research Institute

Magnesium Nebulization Utilization in Management of Pediatric Asthma – "MagNUM PA"

BACKGROUND AND SIGNIFICANCE

Acute asthma is a leading cause of pediatric emergency visits and hospitalizations.[1] In 2005, there were 754,000 pediatric ED asthma visits in the US[2,3], 15-20% of these require hospitalization and another 10-20% relapse within two weeks.[4] However, a 2006 asthma audit of a Canadian pediatric ED shows that 30% of children remaining in moderate and severe distress following initial stabilization therapy are hospitalized and that this population represents 84% of all children admitted to hospital with acute asthma.

Standard therapy of acute severe asthma consists of frequent inhaled $ß_2$ agonists, anticholinergics and corticosteroids.[5-15] However, this regimen has a high degree of outcome heterogeneity since the full benefit of corticosteroids is often not apparent until well beyond the purported 4 hour interval[16] and a substantial proportion of children responds poorly to $ß_2$ agonists (this resistance is in part determined by $ß_2$ adrenoreceptor gene polymorphism).[16-34] Current stabilization therapy[5,14,15] is not always effective in severe attacks[35] and related costs remain high.[36] *Since these insufficient/poor responders represent virtually all pediatric asthma hospitalizations[37] and since hospitalizations account for 43% of the pediatric asthma care costs[38], finding effective strategies to decrease their morbidity is imperative.* Two adjunctive interventions poorly explored in the acute care setting are not ideal for the ED – IV methylxanthines are associated with significant toxicity and no longer recommended[39,40] and IV $ß_2$ agonists are generally reserved for ICU.[41-43]

Mg is a powerful relaxant of airway smooth muscle[44], with a rapid effect when given IV. It relieves bronchoconstriction by decreasing the uptake and release of calcium in bronchial smooth muscle[45], inhibiting release of acetylcholine[46] and of histamine release and stimulating nitric oxide and prostaglandin synthesis.[46] Furthermore, Mg augments the effect of $ß_2$ agonists by upregulating $ß_2$ receptors[47] and also reduces neutrophilic burst seen with the inflammatory response.[48] Mg can be given either IV or by nebulization. Two key meta-analyses confirm that the addition of IV Mg to routine therapy significantly improves hospitalizations and lung function.[49,50]The authors and several major asthma guidelines recommend that IV Mg be considered in children not responding to initial management.[49,51-53] However, our survey "North American Practice Patterns of IV Mg in Severe Acute Asthma in Children" showed that 24% of participants have personally witnessed an Mg-attributed hypotension requiring treatment which, along with the belief that most children with asthma improve without an IV constitute major barriers to the use of IV Mg.[54] These results suggest that adverse effects of IV Mg may not be rare. Furthermore, IV access is much more difficult in young children (who make up the majority of children with asthma) than in adults, and multiple attempts are often required which can lead to an increasing cycle of crying and severe respiratory distress.[55-60] Other theoretical adverse effects after IV Mg administration include apnea and heart block.[61] However, none of the IV or inhaled Mg trials has reported either of these complications.

In contrast, the nebulization route is non-invasive and offers a major advantage of targeted delivery to the lower airway and less potential for side-effects,[62] due to a lower systemic delivery of Mg (1/4 of the IV dose). With IV delivery of Mg, the greatest tissue exposure within the lung is in the alveoli and Mg has to diffuse from the thicker-walled pulmonary and bronchial circulation to reach the smooth muscles of the airways. In contrast, most inhaled Mg would be deposited in the airways and direct diffusion through airway epithelium would result in much higher Mg levels around the smooth muscle as compared with IV delivery. However, the investigation of the efficacy of nebulized Mg has been sparse and has yielded disparate results. Seven studies have compared the benefit of adding nebulized Mg to salbutamol to salbutamol alone[63-69]; only one was limited to children.[64] Almost all studies included asthmatics with negligible admission rates and only one study[63] limited participants to non-responders to bronchodilators who are most likely to benefit from nebulized Mg. This key study by Hughes et al (52 adults) showed a 30% risk reduction in hospitalizations favoring Mg (71% in controls and 43% in the Mg arm).[63] One small study of 62 school-aged children with acute asthma[64] found that a single dose of nebulized Mg added to salbutamol and systemic corticosteroids was associated with a significant improvement in FEV_1 compared to standard therapy at 10 minutes. However, ipratropium was not used, only one patient in each group was hospitalized and the authors did not examine the impact of Mg on other patient outcomes. A recent large RCT demonstrated a significant inhaled Mg effect on an asthma severity score at 60 minutes[70]but did not focus on hospitalizations and the authors did not exclude children who responded to baseline Rx.

A Cochrane systematic review by Blitz[71,72]evaluated 6 trials, 4 of which compared nebulized Mg with $ß_2$ agonists to $ß_2$ agonists alone.[63,64,68,69] There was a clear additive benefit of Mg and salbutamol on lung function in adults with severe disease and a trend towards benefit with respect to lung function and hospitalizations in moderate asthma. A later systematic review[49] of 7 studies[63-69] found an overall treatment effect of Mg and $ß_2$ agonists on both the respiratory function and hospitalization rate approaching statistical significance (p values 0.08 and 0.06, respectively). A recent Cochrane review found improved lung function and a trend toward fewer admissions in patients who received evidence-based baseline therapy[73] and attributes the lack of clear conclusions of inhaled Mg benefit to a small number of patients who were given optimized therapy, i.e. oral steroids with both salbutamol and ipratropium (total N= 247), with concurrent lack of power for using hospitalization as an outcome (N=249). **The *main limitations of past studies are inadequate use of anticholinergics, lack of limiting participants to non-responders to bronchodilators and possible use of inefficient delivery methods.***
The delivery systems used were poorly described and were of low efficiency. Given the encouraging preliminary evidence of benefit, the non-invasiveness and high safety likelihood of the nebulization route and the expertise of our team to ensure Mg delivery, a pediatric study is needed to define the role of nebulized Mg.[49,71] Addition of nebulized Mg should decrease hospitalizations in asthmatic children remaining in moderate to severe distress after optimized baseline treatment which would immediately impact current clinical practice and decrease morbidity of this high-risk population.

We have obtained a peer-reviewed grant for a two-centre version of this trial from the Thrasher Research Fund which has enrolled 124 patients and shows excellent feasibility, lack of side effects and 100% compliance, with no loss to follow up. However, the rate of hospitalizations in this study is higher than anticipated hence the proposed sample size is inadequate to reliably detect a minimum clinically significant difference in hospitalizations. For this reason, we shall need substantially larger sample size (816) to achieve definitive results. This is critically important as preliminary evidence regarding both effectiveness and safety of Mg warrants an adequately powered study.

In view of these arguments, we have submitted this proposal to the RCT committee at the Canadian Institutes for Health Research in March 2013. The study was funded in June 2013 as a Canada-wide seven-center RCT, to commence in the summer of 2014.

Acute asthma is the most common cause of pediatric hospitalizations. While we know that repeat inhalations of $ß_2$ agonists and ipratropium with early oral steroids substantially reduce hospitalizations, many children are resistant to this standard initial therapy. About a third of children remaining in moderate to severe distress after standard therapy are admitted to hospital and comprise 84% of pediatric acute asthma hospitalizations. ***Finding safe, non-invasive, and effective strategies to treat children resistant to standard therapy would substantially decrease hospitalizations resulting in considerable health care savings and reduction of the psycho-social burden of the disease.*** While studies of magnesium sulfate (Mg) given intravenously (IV) suggest that this agent can reduce hospitalizations in both adults and children resistant to standard initial therapy, a North America-wide survey completed by us shows that only 7% of Emergency Department (ED) physicians give IV Mg to prevent hospitalizations, less than 5% of children given IV Mg go home from the ED, and IV Mg is primarily used by physicians to prevent admissions to the ICU. Barriers to IV Mg use include concern about side effects, with 24% of physicians reporting having observed IV Mg-related hypotension requiring treatment as well as a belief that IV therapy is unnecessary. Nebulization is an alternate route for administering Mg. This route has the advantage of being non-invasive and is likely much safer due to lower systemic delivery. Direct delivery via nebulization allows higher Mg concentrations at the target site, the lower airways, with a smaller total drug dose. Two meta-analyses of studies of nebulized Mg – all but one of which have focused on adults - have found that its effect on hospitalizations approaches statistical significance (p=0.08). As a result, the authors of these meta-analyses have ***called for a properly designed study to clarify the role of nebulized Mg. This definitive trial of children in significant respiratory distress after optimized initial therapy will assess the impact of inhaled Mg on hospitalizations, use of medical resources and additional rescue co-interventions***

We plan the following specific aims:

1. **Primary Objective:** To examine if in children with acute asthma remaining in moderate to severe respiratory distress despite maximized initial bronchodilator and steroid therapy there is a reduction in hospitalization rate from the ED in those who receive nebulized Mg with salbutamol versus those receiving salbutamol only.
   Hypothesis: We hypothesize that the children with Pediatric Respiratory Assessment Measure (PRAM) ≥ 5 points after optimized initial inhaled bronchodilator and oral steroid therapies who are given nebulized Mg in addition to nebulized salbutamol will have significantly lower hospitalization rate within 24 hours of starting the study compared to those given salbutamol only.
2. To compare a difference in the changes in the validated Pediatric Respiratory Assessment Measure (PRAM), respiratory rate, oxygen saturation and blood pressure from randomization baseline to 240 minutes in the two groups
3. To determine if there is a significant association between the difference in the primary outcome between the groups and the patient's age, gender, baseline PRAM score, personal history of atopy and "viral-induced wheeze" phenotype.

Hypothesis(es) to be Tested

In this randomized, double-blind seven-centre trial, we hypothesize that children with acute asthma with a Pediatric Respiratory Assessment Measure (PRAM) of ≥ 5 points after optimized initial inhaled bronchodilator and oral steroid therapies who are given nebulized Mg in addition to nebulized salbutamol will have at least a 10 % lower hospitalization rate within 24 hours of starting the study as compared to those given salbutamol only.

SUPPORTIVE PRELIMINARY DATA

North American Practice Patterns of IV Magnesium in Severe Acute Asthma in Children (NAPP SAAC Survey)

Schuh et al, Academic Emergency Medicine, 2010; 17(11): 1189-1196.

We have published a continent-wide survey of the Pediatric Emergency Research Canada network and of Pediatric Emergency Medicine- Collaborative Research Committee consortium (US) entitled "North American Practice Patterns of IV Magnesium Therapy in Severe Acute Asthma in Children" (NAPP SAAC Survey) with the main objective of investigating the frequency of use of IV Mg in stable and critically ill children with severe acute asthma, usual therapeutic goals with respect to disposition and factors impacting the use of this intervention.

Summary of results:
• Response rate to the survey: 70% in Canada and in the United States
• Majority of physicians use IV Mg in less than 20% of children with stable severe acute asthma
• Only 7% of the ED physicians give IV Mg to prevent hospitalizations
• 71% give IV Mg to prevent ICU admission
• Less than 5% of children given IV Mg in the ED are discharged home from the ED
• 24% of the ED physicians have personally witnessed IV Mg related hypotension requiring therapy
•NotablebarrierstotheuseofIVMg:a)concernaboutsideeffectsandb)desiretoavoidanIV
• 97.0 % of physicians felt that if high quality evidence of benefit of nebulized Mg were available, they would incorporate it into their practice and 87.9 % would be willing to participate in such research.

Magnesium Use in Asthma Pharmacotherapy in Canadian Pediatric Emergency Departments: Pediatric Emergency Research Canada Study

Suzanne Schuh, MD, FRCPC[a], Roger Zemek, MD, FRCPC[b], Amy Plint, MD, FRCPC[b], Karen JL Black, MD, FRCPC[c], Stephen Freedman, MD, FRCPC[a], Robert Porter, MD, CCFP (EM)[d], Serge Gouin, MDCM, FRCPC[e], Alexandra Hernandez, MD, FRCPC[f], David Johnson, MD, FAAP [g]

Schuh et al, Pediatrics 2012, 129:852-859.

Abstract

Objectives

To examine the utilization of intravenous magnesium in Canadian Pediatric Emergency Departments (EDs) in children requiring hospitalization for acute asthma and association of administration of frequent albuterol/ipratropium and timely corticosteroids with hospitalization.

Methods

Retrospective medical record review at 6 EDs of otherwise healthy children 2-17 years of age with acute asthma. Data was extracted on history, disease severity, and timing of ED stabilization treatments with inhaled albuterol, ipratropium, corticosteroids and magnesium. Primary outcome was the proportion of hospitalized children given magnesium in the ED. Secondary outcome was the ED use of "intensive therapy" in hospitalized children, defined as three albuterol inhalations with ipratropium and corticosteroids within one hour of triage.

Results

19/154 hospitalized children received magnesium (12.3**%,** 95% CI 7.1; 17.5) versus 2/962 discharged patients. Children given magnesium were more likely to have been previously admitted to ICU (OR 11.2), hospitalized within the past year (OR3.8), received corticosteroids prior to arrival (OR4.0), presented with severe exacerbation (OR 6.1) and to have been treated at one particular centre (OR 14.9). 42/90 (53%) hospitalized children were not given "intensive therapy". Children receiving "intensive therapy" were more likely to present with severe disease to EDs using asthma guidelines (ORs 8.9, 3.0). Differences in the frequencies of all stabilization treatments were significant across centers.

Conclusions

Magnesium is used infrequently in Canadian pediatric EDs in acute asthma requiring hospitalization. Many of these children also do not receive frequent albuterol and ipratropium, or early corticosteroids. Significant variability in the use of these interventions was

detected.

The Choice of a Nebulizer for Delivering Magnesium Sulfate to Pediatric Asthmatic Patients in the Emergency Department

Allan L Coates1, MDCM; Kitty Leung1, BSc; Laurent Vecellio2,3, PhD; Suzanne Schuh4, FRCPC 1 Physiology and Experimental Medicine and 4 Population Health of the Hospital for Sick Children Research Institute, University of Toronto, Toronto Canada and 2 Aerodrug, Tours F-37000 France and 3 INSERM U-618 Université François Rableais, Tours F-37000 France

Coates et al, Respiratory Care Journal, 2011; 56(3):314-8.

Abstract

Background
As the use of intravenous magnesium sulfate (MgSO4) for the treatment of refractory asthma is becoming more common, the incidence of MgSO4 related systemic hypotension is also rising. One potential therapeutic option would be to deliver the MgSO4 by aerosol. One problem with MgSO4 is that, compared to most inhaled medication which is active in the microgram range, it has a dose requirement well into the milligram range. This, along with inefficient delivery systems, may be a reason for the lack of efficacy in some of the published studies using aerosol delivery.

Methods
Prior to a multicenter asthma study in children 2-17years of age evaluating inhaled MgSO4, an *in vitro* study was conducted to choose the best possible delivery system that would be effective over the entire age range. The potential devices considered included the Pari LC Star jet nebulizer, the Omron vibrating membrane device and the AeroNeb Go® vibrating membrane device with the Idehaler® acting as a holding chamber without valves that could connect with a face mask.

Results
The Pari LC Star® had an appropriate particle size distribution but a very slow rate of output. The Omron device had an even slower rate of output and a larger particle size distribution that would have been inappropriate for smaller children. The *in vitro* estimates for lung deposition for the AeroNeb Go® with the Idehaler® were 12.1±0.8 mg/min.

Conclusions
These data would suggest that a 16-minute nebulization session of 6 mL of a solution made up of 2 mL of 500 mg/mL of MgSO4, 1 ml of 5 mg/mL of albuterol and 7 mL of sterile water using the AeroNeb Go® vibrating membrane system attached to the Idehaler® holding chamber with a face mask would maximize delivery of magnesium to the airways in severe asthma while maintaining safety from both the question of bronchospasm due to hypersomolarity of the aerosol and hypotension from systemic absorption. Therefore, this device and regime is recommended for the multicenter trial of inhaled MgSO4 in children with severe asthma.

Pulmonary Deposition with a Novel Aerosol Delivery System

Allan L Coates[1,2] MDCM, Kitty Leung[1] BSc, Jeffrey Chan[3] BSc, Nancy Ribeiro[3] RTNM Martin Charron[3] MD and Suzanne Schuh[4] MD
From Physiology and Experimental Medicine, Research Institute[1], Division of Respiratory Medicine[1,2], Division of Nuclear Medicine[3] and the Department of Emergency Medicine[4] Hospital for Sick Children, University of Toronto. Toronto Canada

Abstract

Background

A problem with intravenous magnesium sulphate ($MgSO_4$) in children and adults with severe acute asthma refractory to optimized standard therapy is systemic hypotension which might be avoided with the aerosol route. However, compared to most inhaled medications which are active in the microgram range, $MgSO_4$ has a dose requirement in the milligram range. This, plus the use of inefficient delivery systems, may explain the lack of efficacy of inhaled $MgSO_4$ in some studies. Prior to a multicenter asthma study in children 2-17 years of age evaluating inhaled $MgSO_4$, an *in vitro* study suggested that the AeroNeb Go® with the Idehaler® using a face mask would have an acceptable
pulmonary delivery of approximately 12 mg/min but no *in vivo* data exist.

Methods

Since the physical characteristics of the sodium and magnesium water suspension are comparable, five adult males had the rate of deposition of normal saline measured using nuclear medicine techniques (to eliminate any Mg-associated risk). Regions of interest comprised of both lungs, the mediastinum with both the trachea and esophagus and the stomach. The measured deposition of the radiolabel was converted to the rate of drug deposition which was compared to the results from an *in vitro* model using adult respiratory patterns.

Results

The mean rate of pulmonary deposition was 10.8±1.9 mg/min (mean±SD) which correlated with height ($r=0.83$, $p<0.05$). The reasons for this slightly lower deposition compared to the *in vitro* estimate include the exclusion of tracheal deposition which would have been included *in vitro* and exhalation of anatomical dead space aerosol which would have been captured on the inspiratory filter *in vitro*. The aforementioned deposition represents 20% of the charge dose, compared to 4% deposition by conventional nebulizers.

Conclusion

The AeroNeb Go® coupled with the novel holding chamber, the Idehaler® did confirm the *in vitro* deposition data in healthy adult males, within expected limits. This device appears suitable for the clinical trial of inhaled MgSO4 over a wide range of ages in patients with refractory asthma. Respiratory Care, December 2013, epub ahead of print.

Development and Evaluation of PRAM[37,83]:

The vast majority of children with acute asthma are of pre-school age and lack coordination to perform pulmonary function tests reliably. Dr Ducharme and colleagues therefore developed and validated the Pediatric Respiratory Assessment Measure (PRAM) as a measure of severity of airway obstruction in acute asthma and its responsiveness to treatment and later evaluated its performance characteristics in children 2 years of age and older presenting with acute asthma in the Emergency Department setting. *This background work will provide us with the ability to use this excellent measurement tool in this trial – both as an entry-severity criterion and as a secondary outcome.*

EXPERIMENTAL DESIGN AND METHODOLOGY

Primary question:

In children 2-17 years of age with acute asthma who have persistent moderate to severe airway obstruction despite maximized initial bronchodilator and steroid therapy, is there a significant difference in the hospitalization rate in those who receive three nebulized Mg and salbutamol treatments compared to those receiving only nebulized salbutamol?

Secondary questions:

Between these treatment modalities:

a). Is there a difference in the changes in the validated Pediatric Respiratory Assessment Measure (PRAM), respiratory rate, oxygen saturation and blood pressure from randomization baseline to 240 minutes?

b). Is there a difference in the number of salbutamol treatments within 240 minutes?

c). Does the treatment effect with respect to primary outcome vary between subgroups defined by these variables: age, gender, pre-randomization PRAM score, personal history of atopy and "viral-induced wheeze" phenotype?

Hypothesis:

We hypothesize that the children with PRAM ≥ 5 points after optimized initial inhaled bronchodilator and oral steroid therapy who are given nebulized Mg in addition to nebulized salbutamol will have a significantly lower hospitalization rate at the index visit compared to those given salbutamol only.

Magnesium Nebulization Utilization in Management of Pediatric Asthma – "MagNUM PA"

Trial Design:

This is a seven-centre randomized double-blind placebo controlled trial. Two study groups will be compared: nebulized salbutamol with Mg sulfate and nebulized salbutamol with placebo.

Triage RN: Baseline PRAM score (**see Appendix A**) in all asthmatics
Moderate to severe disease (PRAM meets local criteria for ipratropium & corticosteroids):
Assessment by ED physician
Systemic corticosteroids
3 treatments with salbutamol & ipratropium over 1 hour
Study nurse notified

↓

Study Nurse repeats PRAM at approximately 1 hour, if ≥ 5: assess eligibility criteria.
(If PRAM is < 5, subject is ineligible for study)

↓

If eligible, obtain informed consent
Baseline measurements (BP, RR, HR, O2 Sat, Obtain treatment allocation assignment
Retrieve treatment package from ED/pharmacy

↓

Experimental Group Treatment
Mg (600 mg) and Salbutamol (5mg)
@ 0', 20', 40'

Control Group Treatment
Placebo and Salbutamol (5mg)
@ 0', 20', 40'

PRAM, RR, HR, O_2 sat @ 0' (pre experimental Rx), 60' (post experimental Rx), 120', 180', 240'
BP @ 0', 20', 40', 60', 120', 180', 240'
(These study procedures can be done approximately at each time point)

↓

Further bronchodilator therapy as clinically indicated

↓

Disposition decision by ED MD

↓

Electronic/paper health chart review and telephone follow-up at 72 hrs (+4 days)

Inclusion criteria:

(1) **2-17 years** of age

(2) **Diagnosis of asthma**, defined as this diagnosis made by a physician or at least one prior acute episode of wheezing with cough or dyspnea treated with inhaled $ß_2$ agonists or oral corticosteroids. *Our study population will exclude bronchiolitis and first-time wheeze (potential alternate diagnoses).*

(3) ***Persistent* moderate to severe airway obstruction** after 3 doses of salbutamol and ipratropium *(as per site specific standard of care guidelines),* defined as a PRAM 5 or higher. *A PRAM score of 5 or more following initial therapy indicates the child has at least moderate disease severity*[37] *and has a high likelihood of being hospitalized.*[37] *This group of children includes 84% of all pediatric asthma hospitalizations; therefore, finding an effective therapy for this population has great potential to significantly reduce hospitalizations. (Appendix B).*

Although the inclusion of children with PRAM scores of 4 or more would enable us to capture nearly all asthma admissions, their admission rate is substantially lower (20%) and thus the overall baseline likelihood of admission would be reduced (Appendix B). Although the admission rate for children with PRAM of 6 or more is high, randomizing only this population would miss 30% of asthma hospitalizations (Appendix B). For these reasons, we have chosen to randomize children with PRAM 5 or more after initial bronchodilator therapy.

Although the PRAM scores of most children will improve following the initial treatment, 35% of those with a presenting PRAM of 5 points do not change (Appendix B). Thus, to maximize capturing this high-risk population, we shall screen and perform post-bronchodilator therapy PRAM scores on all previously healthy children in the target age-range with a presenting PRAM of 5 points or more.

Exclusion Criteria:

(1) No previous history of wheezing or bronchodilator therapy. *Some children who present with wheezing for the first time will have other diagnoses which would not be expected to respond to Mg.*
(2) Patients who have already received IV Mg therapy during the index visit.
(3) Critically ill children requiring immediate intubation. *These children need immediate ICU management and hospitalization.*
(4) Children who in the opinion of the treating physician require a chest radiograph due to atypical clinical presentation and are diagnosed to have lobar consolidation with pneumonia, felt to be the primary cause of respiratory distress. *These rare patients may have to be hospitalized primarily for treatment of the infection and may not respond to magnesium.*
(5) Known co-existent renal, chronic pulmonary, neurologic, cardiac or systemic disease. *These conditions may influence the response to Mg and hospitalization*.
(6) Known hypersensitivity to Mg sulfate.
(7) Patients previously enrolled in the study.
(8) Insufficient command of the English and or French language.
(9) Lack of a home or cellular telephone.
(10) Known allergy/sensitivity to latex.

Sample Selection:

Children presenting to the collaborating EDs at The Hospital for Sick Children, Children's Hospital of Eastern Ontario, Ste Justine's Hospital, BC Children's Hospital, Alberta Children's Hospital, Stollery Hospital and Children's Hospital of Winnipeg who meet eligibility criteria will be approached for enrollment when the research nurses are on duty (days and evenings). The research nurses will keep a log of all children presenting to the ED with acute asthma during the study period whether randomized or not in order to assess the generalizability of the study. All aforementioned hospitals are tertiary care centers, which see the entire clinical and demographic spectrum of the asthma population. Our profile of children with acute asthma should therefore be comparable to that of other institutions and the generalizability of the study should not be affected and the referral bias should be minimal. A structured data collection form will be used to assess the baseline and demographic features that may affect outcome and potentially confound the comparisons. Since the patients will be screened consecutively and study coverage will occur during days, evenings and weekends, selection bias should not play a major role.

Randomization:

The Research Coordinating Pharmacist at SickKids will produce Master Randomization tables, stratified by site and age (≥ 6 years vs less), using a permuted block randomization of 6 and 8 in a 1:1 ratio of active Mg sulfate to placebo, using random number generating software. The Master Randomization tables will be held at the Research Pharmacy at SickKids, open 24 hours a day. Consecutively numbered kits will be prepared by each pharmacy according to the step-by-step procedure manual provided by Research Coordinating Pharmacist at SickKids. Upon receiving the informed consent, the study nurse will obtain the next appropriate numbered study kit from the locked research fridge in the ED (Mg has to be refrigerated) and enter the number in the confidential log book.

Blinding:

The patients, research nurses and ED physicians will be **blinded to the treatment assignment.** The SickKids Research Pharmacist will provide a manual with detailed instructions as to how each site pharmacy will prepare blinded numbered kits containing Mg SO4 or hypertonic 5.5% saline placebo (to match tonicity of Mg Sulfate). Sites will procure a study supply of open label salbutamol nebulizer solution and sterile water to be used as a top up diluent (sterile water chosen as the diluent since mixing normal saline with Mg sulfate is hyperosmolar). Each site will be given detailed requirements for drug accountability and handling to ensure compliance with Health Canada regulations. The active Mg and placebo hypertonic saline mixture with salbutamol and sterile water are very similar in volume, color, taste and smell when nebulized (tested in the research pharmacy at SickKids). The study nurse, physicians and patient will be unaware of the next group assignment. Only the pharmacy will be unblinded. We acknowledge the remote possibility of indirect unblinding because a decrease in blood pressure may occur during Mg therapy. However, major hypotension is unlikely and the likelihood of inadequate

blinding is thus very low. The current inhaled Mg study has no hypotension episodes. Study patients are usually re-assessed after conclusion of the experimental therapy unless they become unstable or a symptomatic drop in blood pressure occurs. Therefore, the ED physicians will be unaware of minor blood pressure fluctuations and the likelihood of unblinding will be minimized. To assess blinding, the research nurse and parents will be asked at the conclusion of experimental therapy which intervention they think the child had received. In case of increasing respiratory distress, IV Mg may be given after the experimental therapy, provided the patient is not hypotensive. In the unlikely event the patient develops hypotension requiring therapy or apnea and the ED physician feels that the experimental therapy cannot be safely continued, further doses of the experimental treatment will be stopped. If these Mg side effects are also accompanied by severe distress and additional IV Mg is warranted, the code may be broken for that patient. Unblinding will only occur if the clinical treatment of the patient will change as a result of knowing which arm of the study the patient was previously on. The study PI/local PI and the study nurses will remain blinded. No patients participating in our inhaled Mg study had experimental therapy unblinded. For emergency unblinding procedures, see **Appendix D**.

Pre-Study Screening and Baseline Evaluation:

All previously healthy children 2-17 years of age with acute asthma will have a PRAM score measured in triage. Those meeting local ED criteria for enhanced therapy (with ipratropium and systemic corticosteroids) will be assessed by the ED physician and receive either oral dexamethasone, oral prednisolone/prednisone or IV hydrocortisone [all considered equivalent for reducing hospitalizations] plus three salbutamol and ipratropium inhalations via Metered Dose Inhaler/Valved Holding Chamber (MDI/VHC)/nebulizer according to the local asthma pathway 20 minutes apart. Ipratropium bromide decreases hospitalizations in asthmatic children with evidence of major distress[90], such as marked neck retractions and extensive wheeze *Our baseline initial therapy is therefore optimized and insufficient improvement/persistent respiratory distress justifies further intervention in this population.*

Study Procedures:

At approximately 1 hour, i.e. at the conclusion of the baseline three inhalations, the research nurse will assess eligibility for the study and measure the pre-randomization PRAM score. Eligible children with PRAM[83] ≥5 points after three bronchodilator treatments [at least moderate to severe respiratory distress] will be approached and informed consent will be obtained. Subjects will be randomly allocated to receive three consecutive nebulizations of salbutamol with either diluted Mg sulfate or diluted hypertonic saline placebo 20 minutes apart (+/- 10 minutes), using the Aeroneb® Go Micropump Nebulizer along with the Idehaler®Pocket system. Since three nebulizations were used in the adult study that demonstrated the greatest benefit of Mg[63], likewise we will use the same number in this study. Specifically, each treatment will utilize 600 mg (1.2 mL) of Mg sulfate (hypertonic)or 1.2 mL hypertonic 5.5% saline (*to match osmolarity of Mg sulfate-see Appendix C for details)*, 5 mg (1 mL) of salbutamol and 3.8 mL of sterile water. *Our Mg dose approximates the upper end of the Mg dosing range used in previous studies, selected to maximize the therapeutic potential of inhaled Mg. Administration of multiple*

experimental inhalations will have the advantage of better drug distribution in the lungs after the first treatment when some bronchoconstriction will have been relieved.

In order to minimize the possibility of cough/bronchospasm which can on occasion be seen with inhaling solutions with osmolarities above 500 mOsm/L[81], we plan to employ a solution with an osmolarity well under 500mOsm/L. In order to ensure that any potential differences in side effects/treatment effect were not due to a difference in the osmolarity of the two solutions, we had to ensure that both the active and placebo arms solutions were of comparable and acceptable osmolarities. *Magnesium sulphate injection solution itself is hyper-osmolar. 5.5% saline has the same osmolarity as magnesium sulphate.*

The use of ***sterile water as the top up diluent in both the active Mg/salbutamol arm and the placebo 5.5% saline/salbutamol arm yields a highly acceptable final osmolality of 384 mOsm/L in both study arms*** (Appendix C). Using normal saline as the top up diluent in the active arm would result in a higher osmolality which would exceed the upper limit of acceptability of 500 mOsm/L. Therefore, normal saline cannot be used as the top up diluent.

The use of 5.5% saline as the placebo and of sterile water as the top up diluent in both arms creates comparable experimental conditions in both study arms (Appendix C). We have also pre-tested that the Idehaler® Pocket system ® nebulizer maintains isotonicity of both active and placebo solutions throughout nebulization, thereby minimizing the possibility of side- effects.

Pre-randomization, the study nurse will measure the subject's PRAM score, respiratory rate, heart rate, oxygen saturation and blood pressure. The study nurse will measure these parameters at 60 minutes and hourly thereafter up to 240 minutes and blood pressure will also be assessed after each experimental nebulization at 20, 40, 60 minutes. These study procedures can be done approximately at each time point. The study nurse will also record the details of all other pharmacotherapy given as well as disposition status during the index visit. The research nurses will ascertain subsequent return visits/hospitalizations-both from the telephone follow-ups as well as from a review of the patient health records including any records from their family doctor if necessary at 72 hours. At this time the parents will also be questioned about unscheduled medical visits related to asthma and further therapies instituted. If families cannot be reached during mutually agreed upon times at 72 hours, daily phone calls will be made until day 7. If hospitalized, patients will not be contacted by the research nurse for a telephone follow-up.

Following this experimental intervention, participating children will continue to receive further salbutamol treatments as frequently as clinically warranted as per the treating ED physician. Disposition will also be determined by the ED physician, independently of the knowledge of the study intervention. If the patient has improved and the ED physician feels that he/she can go home, the patient can be discharged prior to the 240-minute study assessment. Discharged

patients will receive a prescription for inhaled salbutamol via MDI up to every four hours as necessary for the next week in addition to either daily oral prednisolone/prednisone or oral dexamethasone as per local standard of care. All participating families will receive instructions to visit their primary care provider/ED if salbutamol has to be given more often than every 4 hours for increased work of breathing/severe cough and if the respiratory status interferes with usual play/normal speech or routine activity.

<u>The primary outcome measure</u> will be hospitalization defined as admission to an inpatient unit within 24 hours of the start of the experimental therapy due to continued/worsening respiratory distress. Those children in whom a decision to admit was made by the treating physician, but due to lack of bed availability were never transferred to the inpatient unit will be analyzed as admitted as will those returning to the ED within 24 hours of the start of the study who require hospitalization for asthma. It is extremely unlikely that admissions would occur primarily for reasons other than respiratory distress. The study nurse will ascertain that the hospitalizations are for respiratory distress versus other reasons. Should the latter scenario occur, these children will be identified and not counted as hospitalized. Extended ED stays without a decision to admit will not be counted as hospitalized. If the nurse leaves before disposition has been finalized he/she will review the ED electronic data records to identify the length of stay, final disposition, number of bronchodilator treatments by this time and other outcomes the next day. He/she will also communicate with the treating ED physician regarding the reason for hospitalization.

Hospitalization is a powerful marker of treatment failure, a decrease in which is likely to impact practice and influence decision makers since almost a half of pediatric asthma costs, relate to hospitalizations.[91] Hospital admission can also be a very stressful even for both the caregivers and patients. It impacts on the rest of the family since caregivers have to take time off work and arrange alternative sources of care for the other children.

<u>Secondary outcome measures</u>

The two groups will also be compared with respect to:

a. Changes in the PRAM, respiratory rate and oxygen saturation from the start of the first experimental nebulization to 60, 120, 180 and 240 minutes and the changes in the blood pressure from the first experimental nebulization to 20, 40, 60, 120, 180 and 240 minutes.
b. Number of salbutamol treatments within 240 minutes of starting experimental therapy.
c. An association between hospitalization and age, gender, pre-randomization PRAM score, personal history of atopy, and "acute viral induced wheeze" phenotype.[92] This phenotype will be defined by age less than 5 years, co-existent upper respiratory tract infection, no interval symptoms between exacerbations, no atopy. [92-98]
d. Asthma related hospitalization rate by 24 hours of starting Rx to examine Mg impact on side effects such as hypotension necessitating admission.

<u>Other outcomes</u>

Unscheduled visits for asthma to any medical facility within 72 hours of the start of the study. *Most return visits for acute asthma occur within this period.* However, this will be an uncommon event and a meaningful analysis may not be possible.

Major side-effects such as hypotension (systolic blood pressure below 5th percentile for age) or apnea will be tracked as will be admission to ICU for airway stabilization. These outcomes are extremely rare (unstable children will be excluded) and the study cannot therefore be powered for their meaningful statistical analysis. *However, these data are critical to estimate a safety profile of inhaled Mg in children.* We shall not measure the Forced Expiratory Volume in one second (FEV1) since most study patients will be pre-schoolers who cannot perform the necessary maneuvers reliably. Moreover, more than two thirds of the older children with severe asthma enrolled in our previous studies were unable to perform reliable lung function measurements.

PRAM is a validated 12 point clinical asthma severity score[83] exhibiting the most comprehensive measurement properties of all asthma scores[99] which has been successfully used as an outcome in major trials.[100] *It is the only score with demonstrated criterion validity, using respiratory resistance as the gold standard.*[101] This instrument has recently been validated in both preschool and school aged acute asthmatics in the ED *and has strong association with admission, thus supporting its ability to distinguish across severity levels.*[37] *The score has inter-rater reliability consistently above 70%* [37]*and is currently implemented in numerous pediatric EDs across Canada.* In contrast, the Pediatric Asthma Severity Score[102] has not been validated against a concurrent measure of lung function and may not be as responsive as the PRAM due to a smaller range. The vast majority of children treated for acute asthma are preschoolers[103] who lack sufficient coordination to perform pulmonary function tests reliably. All participating EDs now measure the PRAM score as part of routine clinical assessment in their EDs in children with acute asthma. Since Calgary is situated 1000 metres above sea level, oxygen saturations there can be expected to be approximately 2% lower than in Toronto (International Civil Aviation Organization, Manual of the ICAO Standard Atmosphere, Doc 7488-CD, Third Edition, 1993, ISBN 92-9194-004-6). Therefore, the oxygen saturation component of the PRAM will be adjusted in Calgary (this is already local practice) as outlined in Appendix A.

<u>Study Implementation:</u>

Prior to the study, the ED staff physicians and fellows and emergency nurses will be educated in all aspects of the study. Particular attention will be paid to the importance of communicating to the research nurse the reasons for hospitalization and the importance of protocolized stabilization therapy. The research nurses will be trained in all aspects of the study execution,

including obtaining informed consent, technical aspects of administering nebulized treatments and the PRAM measurement.

This study requires the following personnel:

1. Study manager at The Hospital for Sick Children who will communicate with the PI, the site PIs and all study nurses regarding starting the study at all sites, data transfer, study-related enrollment and logistic issues, facilitate the REB-related matters as well as oversee the budget and organize the study log in Toronto.
2. Research nurses or respiratory therapists at all participating sites will be responsible for screening, enrollment and study execution and electronic data entry as well as the data transfer.

Sample Size:

The sample size calculation is based on the assessment of the between-group difference in proportions of hospitalizations. The estimated hospitalization rate is based on our pilot data where the *overall* (*control plus intervention*) hospitalization rate is 40%. Since this ongoing pilot remains blinded, it is certainly feasible that the *control group hospitalization rate* may be as high as 50%. This admission rate is greater than that in a 2006 prospective audit of 1000 children presenting with acute asthma at Canadian EDs which showed that approximately 30% of patients with a PRAM score of ≥ 5 after bronchodilator therapy were hospitalized (Appendix B).While the admission rate in our current study is substantially higher than in the previous audit, the one study in adults that focused on non-respondents to optimized initial Rx had an even higher admission rate of 71%.[63] *In order to ensure adequate power, we have conservatively used the hospitalization rate from our pilot* as compared to lower estimates using historical data. This is a superiority study in which the adoption of the Mg therapy can only be recommended for future practice if the rate of the primary outcome in this group is significantly lower than in the controls. With 408 patients per arm (816 in total) a two-sided test with a type I error of 0.05 will have 80% power to achieve statistical significance if Mg therapy reduces the probability of hospitalization to 40% (i.e. absolute reduction of 10%) [104] . This estimate is based on clinically relevant differences agreed upon by all study authors and it also represents NNT of 10. In the Cochrane reviews of anticholinergics and early corticosteroids by Plotnick and Rowe, respective NNTs of 12 and 8 led to a change in national practice recommendations.[105,106] In our North America-wide survey the majority of respondents considered a 10% reduced risk as a minimally clinically important difference that would prompt adoption of Mg.[54] Since almost a half of pediatric asthma costs relates to hospitalizations, this target difference would also have significant economic impact. Since our pilot has already enrolled 124 patients, 692 additional subjects need to be recruited. Based on the current study, the anticipated refusal rate will be 24%. Although the study non-completion rate and loss to follow-up are both currently 0%, we assume that each may be as high as 5%. Therefore, to have complete data on 692 patients we plan to randomize 766 (*i.e.* 692/(1 - 0.05)*(1 – 0.05) and to approach 1008 (*i.e.* 766/(1 - 0.24).

.

Statistical Analysis:

The primary analysis:

A two-sided Fisher's exact test will be used to test the null hypothesis that the treatment arms are equal with respect to the probability of hospitalization. This analysis will be performed on all randomized patients, according to the intent-to-treat principle, using a two-sided test of hypothesis with a type I error of 0.05. A nominal level of 4% for the type I error rate will be used to account for the interim analysis.

The secondary analyses:

a) Repeated measures ANOVA to compare treatment arms with respect to the changes in the PRAM score, respiratory rate, heart rate, oxygen saturation, and blood pressure over time.
b) A Poisson model will be used to compare the number of salbutamol treatments used in the ED in the two study arms.
c) Logistic regression analysis, including interaction terms with treatment group, will be used to examine the subgroup effects with respect to the primary outcome. The following variables will be used to define subgroups: age, gender, pre-randomization PRAM score, personal history of atopy.
The statistical tests of hypotheses for the secondary outcomes a) through c will two-sided at the 0.017 level to account for the issue of multiple testing and to maintain an overall type 1 error rate of 0.05.

Interim Analysis:

To assure safety, there will be one planned interim analysis on the first 200 patients randomized (a quarter through the study) conducted by a statistician not involved in the trial and evaluated by the independent data safety monitoring board. The interim analysis will be a one-sided test of the null hypothesis of no difference versus the alternative hypothesis that the probability of hospitalization is higher on Mg therapy at the 0.01 level. That is, we are looking for evidence that Mg therapy is less effective, and the trial will be stopped at an interim analysis only if the null hypothesis is rejected in favor of the control arm. Therefore, the interim analysis is only for safety and not for efficacy and it will not increase the probability of erroneously rejecting the null hypothesis in favor of Mg therapy at the final analysis. The reason we are doing one-sided (for harm) interim analysis is because if there is early strong evidence that Mg increases the probability of hospital admission, we want to stop the trial. On the other hand, we do not want to stop the trial early for benefit because a smaller sample size will not be convincing.

Feasibility:

We plan to implement an enrollment schedule similar to the one used in the current study, for a total of 88 hours a week. Extensive weekly coverage is needed since the time of presentation of these children varies. These hours will be covered by a combination of clinical research nurse coordinators and several trained on call research nurses. Based on the current pilot study, approximately 1337 patients ≥2 years of age present to SickKids and Alberta Children's annually, 87 of which were enrolled and completed the study in **one asthma season** (**Appendix E**). Based on the current study logs, we anticipate a 7% miss rate, 17% eligibility, 24% refusal rate, 5% may not finish the full experimental Rx (0% to date) and 5% may be lost to follow up (0% to date). Based on these enrollment rates and annual asthma presentations to the participating EDs, 3994 children can be expected to present to the participating EDs, of which 2006 (50%) will be screened, 342 will be eligible and 260 are projected to be randomized. Although 100% patients enrolled to date have completed experimental Rx and both the 24 hour and 7 day follow up, up to 5% may not fulfill either requirement, leaving 234 patients with full data per asthma season (**Appendix E**). Therefore, 3.2 asthma seasons (31 study months) which will include 4 fall periods when asthma presentations are the most plentiful represent a reasonable timeline for obtaining the required sample size. Since *virtually all asthma cases occur between September and May, these totals represent one "asthma season".* To save money, enrollment will be limited to these periods. ***TIMELINE:*** **Oct'13-Apr'14:** regulatory documents, investigator meetings, REB, distribution of nebulizers, **Jan-Apr 14:** hiring of personnel, **May-Aug '14:** personnel training, **Sept '14-May '15:** 1st year recruitment, **Sept '15- May '16**: 2nd year recruitment, **Sept'16-May'17** 3rd year of recruitment**, June "17-Dec 2020**- last recruitment, 2021: Data management, analysis,: Abstract and manuscript preparation.

Compliance with the experimental therapy is expected to be excellent since the nurses will administer and supervise its delivery in all children and the entire intervention will take place in the ED. They will also ensure the nebulizer mask stays on the face throughout treatment. We have conducted numerous past studies with successful nebulized bronchodilator delivery with a mask-face seal facilitated by the research nurse.[108-119] The experimental period is very short which will also enhance compliance. In our extensive experience, virtually no patients fail to finish experimental therapy. We have adjusted the sample size by 5% to account for /loss to follow-up.

Adverse Events

Magnesium blocks the neuromuscular transmission and acts as a CNS depressant. Therefore, the theoretical adverse effects with IV Mg may include a transient drop in blood pressure, apnea and heart block.[61] None of the IV or inhaled Mg trials has reported any of these issues and none of these have occurred during the pilot phase of the study. One study detected burning at the IV site, flushing and fatigue.[120] In their systematic review, Rowe et al. reported a clinically non- significant decrease in blood pressure.[121] However, hypotension related to IV Mg does occur, as documented in our North-America-wide survey. None of the surveyed physicians have witnessed heart block related to IV Mg and ‹1% have witnessed apnea. The potential for

these problems after nebulized Mg is much lower than with IV Mg since this treatment route will result in a lower systemic delivery of Mg (1/4 of the IV dose) and a lower systemic effect. Of note, a recent Cochrane review of 896 patients given inhaled Mg confirmed the safety of this agent.[73] No child in the current inhaled Mg study had experienced hypotension or other side effects.

All unexpected adverse events will be reported to the Hospital for Sick Children Research Ethics Board according to the Hospital for Sick Children's adverse event reporting requirements. Unexpected adverse events will be classified as mild, moderate or severe. Expected adverse events will include cough, respiratory distress (disease-related), asthma-related hospitalization, IV insertion, sinus tachycardia, nausea and bitter/salty taste of the experimental solution. All serious, unexpected adverse drug reactions to the study medication will be reported to Health Canada within 15 calendar days or for death or life-threatening events, within 7 calendar days. In the latter case, a follow-up report must be filed within 8 calendar days. Adverse reactions will be managed according to the Hospital for Sick Children's standard clinical management practices. Furthermore, we plan to document episodes of severe cough necessitating interruption of the experimental therapy for more than approximately 3 minutes to examine the safety profile of magnesium.

The serious adverse events will consist of hypotension below the 5th percentile for age, apnea and admission to intensive care unit. These will be reported to the PI, SickKids REB, local REB and the DSMC.

Since hypotension is the only major side-effect of IV Mg occurring with appreciable frequency, all enrolled patients will be on precautionary frequent blood pressure monitoring as per the study protocol. If the systolic blood pressure drops below 5th percentile for age, the study will be stopped, treatment given as necessary and DSMC will be notified. This has not happened during the current pilot phase of the study.

Due to the osmolarity of the study solutions being well under 500 mOsm/L throughout nebulization and co-administration of salbutamol, we do not anticipate side effects to occur as a result of using the aforementioned composition of the study solutions. However, should the highly unlikely event of respiratory deterioration occur, the experimental therapy will be discontinued, appropriate additional treatment started and the event will be reported to the DSMC within 48 hours. Salbutamol may cause tachycardia and this was also the case in many children enrolled to date. However, this was uniformly well tolerated and no patient had to stop/interrupt experimental therapy due to this issue.

To ensure safety of the participating subjects, unstable children requiring immediate airway stabilization will be excluded. We are also planning an interim analysis to maximize safety.

Data Safety and Monitoring Committee (DSMC):

The Data Safety and Monitoring Committee (DSMC) will consist of a non-study biostatistician, an ED physician and researcher and an ED scientist. The members of this committee will not be collaborators of this trial. They will be notified of all serious adverse events (such as hypotension <5th percentile for age, apnea, heart block, severe increase in respiratory distress necessitating discontinuation of the study) and of an admission to the ICU within 48 hours. Should any of these adverse events occur, they will be immediately reported from both sites to the study coordinator at SickKids who will promptly notify the DSMC. The DSMC will meet once per asthma season or ad hoc if necessary.

Dissemination of Results and Future Directions: The results of this study will be submitted for presentation at either the annual meeting of the Pediatric Academic Societies, the Society for Academic Emergency Medicine or the American Academy of Pediatrics. We shall also submit the manuscript for publication in a peer-reviewed scientific journal.

Limitations: In this study, we anticipate a very low rate of magnesium-related side effects such as hypotension. The major reason for this is a limited systemic magnesium delivery, which will be much lower than with the IV therapy. However, the study sample size will not permit us to conduct a meaningful statistical analysis of magnesium-related adverse events since we anticipate an extremely small number of such events, if any.

Literature References

1. Mannino DM, Homa DM, Pertowski CA, et al. Surveillance for asthma--United States, 1960-1995. Morbidity & Mortality Weekly Report CDC Surveillance Summaries 1998;47:1-27.
2. Asthma prevalence, health care use and mortality: United States, 2003-2005. Centers for Disease Control and Prevention, November 2006. (Accessed October 1, 2009, at http://www.cdc.gov/nchs/data/hestat/ashtma03-05/asthma03-05.htm.)
3. Moorman JE, Rudd RA, Johnson CA, et al. National surveillance for asthma--United States, 1980-2004. Morbidity & Mortality Weekly Report Surveillance Summaries 2007;56:1-54.
4. Camargo CA, Investigators M. Acute asthma among children presenting to the emergency department: the Multicenter Asthma Research Collaboration. Acad Emerg Med 1998;5:380.
5. Scarfone RJ, Fuchs SM, Nager AL, Shane SA. Controlled trial of oral prednisone in the emergency department treatment of children with acute asthma.[see comment]. Pediatrics 1993;92:513-8.
6. Robertson CF, Smith F, Beck R, Levison H. Response to frequent low doses of nebulized salbutamol in acute asthma. J Pediatr 1985;106:672-4.
7. Schuh S, Parkin P, Rajan A, et al. High-versus low-dose, frequently administered, nebulized albuterol in children with severe, acute asthma. Pediatrics 1989;83:513-8.
8. Plotnick LH, Ducharme FM. Combined inhaled anticholinergic agents and beta-2-agonists for initial treatment of acute asthma in children.[update in Cochrane Database Syst Rev. 2000;(4):CD000060; PMID: 11034671]. Cochrane Database of Systematic Reviews 2000:CD000060.
9. Rowe BH, Edmonds ML, Spooner CH, Camargo CA. Evidence-based treatments for acute asthma. Respir Care 2001;46:1380-90.
10. National Heart, Lung and Blood Institute: Global Initiative for asthma. Global strategy for asthma management and prevention. NHLBI/WHO workshop report. Bethesda, Md: NIH; 2002. Report No.: 02-3659.
11. National Institutes of Health and National Heart, Lung, and Blood Institute. Guidelines for the Diagnosis and Management of Asthma - Update on Selected Topics 2002. Washington, DC: US Dept of Health and Human Services; 2002. Report No.: 97-4051.
12. National Asthma Council. Asthma Management Handbook 2002. Melbourne: National Asthma Council Australia, Ltd; 2002.
13. British Thoracic S, Scottish Intercollegiate Guidelines N. British guideline on the management of asthma. Thorax 2003;58 Suppl 1:i1-94.
14. Becker A, Lemiere C, Berube D, et al. Summary of recommendations from the Canadian Asthma Consensus guidelines, 2003. CMAJ Canadian Medical Association Journal 2005;173:S3-11.
15. National Asthma Education and Prevention Program. Expert Panel Report 3: guidelines for the diagnosis and management of asthma. Bethesda, MD: National Institutes of Health, National Heart, Lung, and Blood Institute; 2007.
16. Hall IP. Pharmacogenetics of asthma. Eur Respir J 2000;15:449-51.
17. Drazen JM, Silverman EK, Lee TH. Heterogeneity of therapeutic responses in asthma. Br Med Bull 2000;56:1054-70.
18. Palmer LJ, Silverman ES, Weiss ST, Drazen JM. Pharmacogenetics of asthma. Am J Respir Crit Care Med 2002;165:861-6.
19. Koga T, Kamimura T, Oshita Y, et al. Determinants of bronchodilator responsiveness in patients with controlled asthma. J Asthma 2006;43:71-4.

20. Tsai HJ, Shaikh N, Kho JY, et al. Beta 2-adrenergic receptor polymorphisms: pharmacogenetic response to bronchodilator among African American asthmatics. Hum Genet 2006;119:547-57.
21. Johnson M. The beta-adrenoceptor. Am J Respir Crit Care Med 1998;158:S146-53.
22. Taylor DR, Epton MJ, Kennedy MA, et al. Bronchodilator response in relation to beta2-adrenoceptor haplotype in patients with asthma. Am J Respir Crit Care Med 2005;172:700-3.
23. Lipworth BJ, Hall IP, Tan S, Aziz I, Coutie W. Effects of genetic polymorphism on ex vivo and in vivo function of beta2-adrenoceptors in asthmatic patients. Chest 1999;115:324-8.
24. Israel E, Drazen JM, Liggett SB, et al. The effect of polymorphisms of the beta(2)-adrenergic receptor on the response to regular use of albuterol in asthma. Am J Respir Crit Care Med 2000;162:75-80.
25. Taylor DR, Drazen JM, Herbison GP, Yandava CN, Hancox RJ, Town GI. Asthma exacerbations during long term beta agonist use: influence of beta(2) adrenoceptor polymorphism. Thorax 2000;55:762-7.
26. Israel E, Chinchilli VM, Ford JG, et al. Use of regularly scheduled albuterol treatment in asthma: genotype-stratified, randomised, placebo-controlled cross-over trial.[see comment]. Lancet 2004;364:1505-12.
27. Palmer CN, Lipworth BJ, Lee S, Ismail T, Macgregor DF, Mukhopadhyay S. Arginine-16 beta2 adrenoceptor genotype predisposes to exacerbations in young asthmatics taking regular salmeterol.[see comment]. Thorax 2006;61:940-4.
28. Contopoulos-Ioannidis DG, Manoli EN, Ioannidis JP. Meta-analysis of the association of beta2-adrenergic receptor polymorphisms with asthma phenotypes.[see comment]. J Allergy Clin Immunol 2005;115:963-72.
29. Martinez FD, Graves PE, Baldini M, Solomon S, Erickson R. Association between genetic polymorphisms of the beta2-adrenoceptor and response to albuterol in children with and without a history of wheezing. J Clin Invest 1997;100:3184-8.
30. Carroll CL, Schramm CM, Zucker AR. Slow-responders to IV beta2-adrenergic agonist therapy: defining a novel phenotype in pediatric asthma. Pediatr Pulmonol 2008;43:627-33.
31. Liggett SB. Polymorphisms of the beta2-adrenergic receptor and asthma. Am J Respir Crit Care Med 1997;156:S156-62.
32. Thakkinstian A, McEvoy M, Minelli C, et al. Systematic review and meta-analysis of the association between {beta}2-adrenoceptor polymorphisms and asthma: a HuGE review. Am J Epidemiol 2005;162:201-11.
33. Choudhry S, Ung N, Avila PC, et al. Pharmacogenetic differences in response to albuterol between Puerto Ricans and Mexicans with asthma.[see comment]. Am J Respir Crit Care Med 2005;171:563-70.
34. Martin AC, Zhang G, Rueter K, et al. Beta2-adrenoceptor polymorphisms predict response to beta2-agonists in children with acute asthma. J Asthma 2008;45:383-8.
35. Kercsmar CM, McDowell KM. Love it or lev it: levalbuterol for severe acute asthma--for now, leave it.[comment]. J Pediatr 2009;155:162-4.
36. Weiss KB, Sullivan SD, Lyttle CS. Trends in the cost of illness for asthma in the United States, 1985-1994. J Allergy Clin Immunol 2000;106:493-9.
37. Ducharme FM, Chalut D, Plotnick L, et al. The Pediatric Respiratory Assessment Measure: a valid clinical score for assessing acute asthma severity from toddlers to teenagers. J Pediatr 2008;152:476-80.
38. Ungar WJ, Coyte PC, Pharmacy Medication Monitoring Program Advisory B. Prospective study of the patient-level cost of asthma care in children. Pediatr Pulmonol 2001;32:101-8.
39. Parameswaran K, Belda J, Rowe BH. Addition of intravenous aminophylline to beta2-agonists in adults with acute asthma. Cochrane Database of Systematic Reviews 2000:CD002742.
40. Littenberg B. Aminophylline treatment in severe, acute asthma. A meta-analysis. JAMA 1988;259:1678-84.

41. Browne GJ, Lam LT. Single-dose intravenous salbutamol bolus for managing children with acute severe asthma in the emergency department:Reanalysis of data. Pediatr Crit Care Med 2002;3:117-23.
42. Browne GJ, Penna AS, Phung X, Soo M. Randomised trial of intravenous salbutamol in early management of acute severe asthma in children. Lancet 1997;349:301-5.
43. Browne GJ, Trieu L, Van Asperen P. Randomized, double-blind, placebo-controlled trial of intravenous salbutamol and nebulized ipratropium bromide in early management of severe acute asthma in children presenting to an emergency department. Crit Care Med 2002;30:448-53.
44. Spivey WH, Skobeloff EM, Levin RM. Effect of magnesium chloride on rabbit bronchial smooth muscle. Ann Emerg Med 1990;19:1107-12.
45. Iseri LT, French JH. Magnesium: nature's physiologic calcium blocker. Am Heart J 1984;108:188-93.
46. Hill J, Britton J. Dose-response relationship and time-course of the effect of inhaled magnesium sulphate on airflow in normal and asthmatic subjects. Br J Clin Pharmacol 1995;40:539-44.
47. Rolla G, Bucca C, Brussino L, Colagrande P. Effect of intravenous magnesium infusion on salbutamol-induced bronchodilatation in patients with asthma. Magnes Res 1994;7:129-33.
48. Cairns CB, Kraft M. Magnesium attenuates the neutrophil respiratory burst in adult asthmatic patients.[see comment]. Acad Emerg Med 1996;3:1093-7.
49. Mohammed S, Goodacre S. Intravenous and nebulised magnesium sulphate for acute asthma: systematic review and meta-analysis. Emerg Med J 2007;24:823-30.
50. Cheuk DK, Chau TC, Lee SL. A meta-analysis on intravenous magnesium sulphate for treating acute asthma. Arch Dis Child 2005;90:74-7.
51. Global Initiative for Asthma. (Accessed October 5, 2009, at www.ginasthma.com.)
52. Canadian Asthma Consensus Group. Management of patients with asthma in the emergency department and in hospital. CMAJ Canadian Medical Association Journal 1999;161:S53-9.
53. British guideline on the management of asthma. 2009. (Accessed October 23, 2009, at http://www.brit-thoracic.org.uk/Portals/0/Clinical%20Information/Asthma/Guidelines/sign101%20revised%20June%2009.pdf.)
54. Schuh S, Macias C, Freedman S, et al. North American Practice Patterns of IV Magnesium Therapy in Severe Acute Asthma in Children. Acad Emerg Med 2010;17:1189-96.
55. Taddio A, Soin HK, Schuh S, Koren G, Scolnik D. Liposomal lidocaine to improve procedural success rates and reduce procedural pain among children: a randomized controlled trial.[see comment]. CMAJ Canadian Medical Association Journal 2005;172:1691-5.
56. Black KJ, Pusic MV, Harmidy D, McGillivray D. Pediatric intravenous insertion in the emergency department: bevel up or bevel down? Pediatr Emerg Care 2005;21:707-11.
57. Arts SE, Abu-Saad HH, Champion GD, et al. Age-related response to lidocaine-prilocaine (EMLA) emulsion and effect of music distraction on the pain of intravenous cannulation. Pediatrics 1994;93:797-801.
58. Manner T, Kanto J, Iisalo E, Lindberg R, Viinamaki O, Scheinin M. Reduction of pain at venous cannulation in children with a eutectic mixture of lidocaine and prilocaine (EMLA cream): comparison with placebo cream and no local premedication. Acta Anaesthesiol Scand 1987;31:735-9.
59. Taddio A, Gurguis MG, Koren G. Lidocaine-prilocaine cream versus tetracaine gel for procedural pain in children. Ann Pharmacother 2002;36:687-92.
60. Walco GA, Burns JP, Cassidy RC. The ethics of pain control in infants and children. In: Schechter NL, Berde CB, Yaster M, eds. Pain in infants, children, and adolescents. 2nd ed. New York: Lippincott Williams & Wilkins; 2003:157-68.

61. Magnesium Sulphate: Adverse Effects. In: Reynolds JEF, Parfitt K, Parsons AV, Sweetman SC, Council of the Royal Pharmaceutical Society of Great Britain, Department of Pharmaceutical Sciences, eds. The extra pharmacopoeia. 29th ed. London: Pharmaceutical Press; 1989:1033.
62. Okayama H, Aikawa T, Okayama M, Sasaki H, Mue S, Takishima T. Bronchodilating effect of intravenous magnesium sulfate in bronchial asthma. JAMA 1987;257:1076-8.
63. Hughes R, Goldkorn A, Masoli M, Weatherall M, Burgess C, Beasley R. Use of isotonic nebulised magnesium sulphate as an adjuvant to salbutamol in treatment of severe asthma in adults: randomised placebo-controlled trial.[see comment]. Lancet 2003;361:2114-7.
64. Mahajan P, Haritos D, Rosenberg N, Thomas R. Comparison of nebulized magnesium sulfate plus albuterol to nebulized albuterol plus saline in children with acute exacerbations of mild to moderate asthma. J Emerg Med 2004;27:21-5.
65. Aggarwal P, Sharad S, Handa R, Dwiwedi SN, Irshad M. Comparison of nebulised magnesium sulphate and salbutamol combined with salbutamol alone in the treatment of acute bronchial asthma: a randomised study. Emergency Medicine Journal 2006;23:358-62.
66. Drobina BJ, Kostic MA, Roos JA. Nebulized magnesium has no benefit in the treatment of acute asthma in the emergency department. Acad Emerg Med 2006;13.
67. Kokturk N, Turktas H, Kara P, Mullaoglu S, Yilmaz F, Karamercan A. A randomized clinical trial of magnesium sulphate as a vehicle for nebulized salbutamol in the treatment of moderate to severe asthma attacks.[see comment]. Pulm Pharmacol Ther 2005;18:416-21.
68. Bessmertny O, DiGregorio RV, Cohen H, et al. A randomized clinical trial of nebulized magnesium sulfate in addition to albuterol in the treatment of acute mild-to-moderate asthma exacerbations in adults. Ann Emerg Med 2002;39:585-91.
69. Nannini LJ, Pendino JC, Corna RA, Mannarino S, Quispe R. Magnesium sulfate as a vehicle for nebulized salbutamol in acute asthma. The American journal of medicine 2000;108:193-7.
70. Powell C. A randomised, double blind, placebo controlled study of nebulised magnesium sulphate in asute asthma in children – The MAGNETIC study. Archives of Disease in Childhood 2012;97:A2-A3.
71. Blitz M, Blitz S, Hughes R, et al. Aerosolized magnesium sulfate for acute asthma: a systematic review.[see comment][erratum appears in Chest. 2005 Nov;128(5):3779]. Chest 2005;128:337-44.
72. Blitz M, Blitz S, Beasely R, et al. Inhaled magnesium sulfate in the treatment of acute asthma.[update in Cochrane Database Syst Rev. 2005;(4):CD003898; PMID: 16235345][update of Cochrane Database Syst Rev. 2005;(2):CD003898; PMID: 15846687]. Cochrane Database of Systematic Reviews 2005:CD003898.
73. Powell C, Dwan K, Milan SJ, et al. Inhaled magnesium sulfate in the treatment of acute asthma. The Cochrane database of systematic reviews 2012;12:CD003898.
74. Coates A, Leung K, Vecellio None L, Schuh S. The Choice of a Nebulizer for Delivering Magnesium Sulfate to Pediatric Asthmatic Patients in the Emergency Department. Respir Care In Press.
75. Coates AL, MacNeish CF, Allen PD, Ho SL, Lands LC. Do sinusoidal models of respiration accurately reflect the respiratory events of patients breathing on nebulizers? J Aerosol Med 1999;12:265-73.
76. Katz SL, Adatia I, Louca E, et al. Nebulized therapies for childhood pulmonary hypertension: an in vitro model. Pediatr Pulmonol 2006;41:666-73.
77. Coates AL, Dinh L, MacNeish CF, et al. Accounting for radioactivity before and after nebulization of tobramycin to insure accuracy of quantification of lung deposition. J Aerosol Med 2000;13:169-78.
78. Coates AL, Tipples G, Leung K, Gray M, Louca E, Vaccine WHOPDGfMA. How many infective viral particles are necessary for successful mass measles immunization by aerosol? Vaccine 2006;24:1578-85.

79. Mitchell JP, Nagel MW, Nichols S, Nerbrink O. Laser diffractometry as a technique for the rapid assessment of aerosol particle size from inhalers. J Aerosol Med 2006;19:409-33.
80. Coates AL, Macneish CF, Lands LC, et al. Factors influencing the rate of drug output during the course of wet nebulization. Journal of Aerosol Medicine-Deposition Clearance and Effects in the Lung 1998;11:101-11.
81. Dodd ME, Abbott J, Maddison J, Moorcroft AJ, Webb AK. Effect of tonicity of nebulised colistin on chest tightness and pulmonary function in adults with cystic fibrosis. Thorax 1997;52:656-8.
82. Ciarallo L, Brousseau D, Reinert S. Higher-dose intravenous magnesium therapy for children with moderate to severe acute asthma. Arch Pediatr Adolesc Med 2000;154:979-83.
83. Chalut DS, Ducharme FM, Davis GM. The Preschool Respiratory Assessment Measure (PRAM): a responsive index of acute asthma severity.[see comment]. J Pediatr 2000;137:762-8.
84. Chua HL, Collis GG, Newbury AM, et al. The influence of age on aerosol deposition in children with cystic fibrosis. Eur Respir J 1994;7:2185-91.
85. Newhouse MT, Dolovich MB. Control of asthma by aerosols.[see comment]. N Engl J Med 1986;315:870-4.
86. Tal A, Golan H, Grauer N, Aviram M, Albin D, Quastel MR. Deposition pattern of radiolabeled salbutamol inhaled from a metered-dose inhaler by means of a spacer with mask in young children with airway obstruction. J Pediatr 1996;128:479-84.
87. Wildhaber JH, Dore ND, Wilson JM, Devadason SG, LeSouef PN. Inhalation therapy in asthma: nebulizer or pressurized metered-dose inhaler with holding chamber? In vivo comparison of lung deposition in children.[see comment]. J Pediatr 1999;135:28-33.
88. Leung K, Louca E, Coates AL. Comparison of breath-enhanced to breath-actuated nebulizers for rate, consistency, and efficiency. Chest 2004;126:1619-27.
89. Wildhaber JH, Devadason SG, Hayden MJ, Eber E, Summers QA, LeSouef PN. Aerosol delivery to wheezy infants: a comparison between a nebulizer and two small volume spacers. Pediatr Pulmonol 1997;23:212-6.
90. Qureshi F, Pestian J, Davis P, Zaritsky A. Effect of nebulized ipratropium on the hospitalization rates of children with asthma. N Engl J Med 1998;339:1030-5.
91. Kopach R, Sadat S, Gallaway ID, Geiger G, Ungar WJ, Coyte PC. Cost-effectiveness analysis of medical documentation alternatives. Int J Technol Assess Health Care 2005;21:126-31.
92. Brand PL, Baraldi E, Bisgaard H, et al. Definition, assessment and treatment of wheezing disorders in preschool children: an evidence-based approach. Eur Respir J 2008;32:1096-110.
93. Ducharme FM, Lemire C, Noya FJ, et al. Preemptive use of high-dose fluticasone for virus-induced wheezing in young children.[see comment]. N Engl J Med 2009;360:339-53.
94. Oommen A, Lambert PC, Grigg J. Efficacy of a short course of parent-initiated oral prednisolone for viral wheeze in children aged 1-5 years: randomised controlled trial.[see comment]. Lancet 2003;362:1433-8.
95. Vuillermin PJ, Robertson CF, South M. Parent-initiated oral corticosteroid therapy for intermittent wheezing illnesses in children: systematic review.[see comment]. J Paediatr Child Health 2007;43:438-42.
96. Johnston SL, Pattemore PK, Sanderson G, et al. Community study of role of viral infections in exacerbations of asthma in 9-11 year old children.[see comment]. BMJ 1995;310:1225-9.
97. Martinez FD, Wright AL, Taussig LM, Holberg CJ, Halonen M, Morgan WJ. Asthma and wheezing in the first six years of life. The Group Health Medical Associates.[see comment]. N Engl J Med 1995;332:133-8.
98. Castro-Rodriguez JA, Holberg CJ, Wright AL, Martinez FD. A clinical index to define risk of asthma in young children with recurrent wheezing. Am J Respir Crit Care Med 2000;162:1403-6.

99. Birken CS, Parkin PC, Macarthur C. Asthma severity scores for preschoolers displayed weaknesses in reliability, validity, and responsiveness. J Clin Epidemiol 2004;57:1177-81.
100. Panickar J, Lakhanpaul M, Lambert PC, et al. Oral prednisolone for preschool children with acute virus-induced wheezing. N Engl J Med 2009;360:329-38.
101. Ducharme FM, Davis GM. Respiratory resistance in the emergency department: a reproducible and responsive measure of asthma severity.[see comment]. Chest 1998;113:1566-72.
102. Gorelick MH, Stevens MW, Schultz TR, Scribano PV. Performance of a novel clinical score, the Pediatric Asthma Severity Score (PASS), in the evaluation of acute asthma. Acad Emerg Med 2004;11:10-8.
103. Akinbami LJ, Schoendorf KC. Trends in childhood asthma: prevalence, health care utilization, and mortality. Pediatrics 2002;110:315-22.
104. Fleiss JL. Statistical Methods for Rates and Proportions. Second Edition: Wiley, John and Sons, Incorporated, New York, N.Y.; 1981.
105. Plotnick LH, Ducharme FM. Combined inhaled anticholinergics and beta2-agonists for initial treatment of acute asthma in children. The Cochrane database of systematic reviews 2000:CD000060.
106. Rowe BH, Spooner C, Ducharme FM, Bretzlaff JA, Bota GW. Early emergency department treatment of acute asthma with systemic corticosteroids. The Cochrane database of systematic reviews 2001:CD002178.
107. Fleiss JL. Statistical methods for rates and proportions. 2nd ed. New York, NY: Wiley; 1981.
108. Schuh S, Canny G, Reisman JJ, et al. Nebulized albuterol in acute bronchiolitis. J Pediatr 1990;117:633-7.
109. Schuh S, Reider MJ, Canny G, et al. Nebulized albuterol in acute childhood asthma: comparison of two doses. Pediatrics 1990;86:509-13.
110. Bentur L, Canny GJ, Shields MD, et al. Controlled trial of nebulized albuterol in children younger than 2 years of age with acute asthma. Pediatrics 1992;89:133-7.
111. Schuh S, Johnson D, Canny G, et al. Efficacy of adding nebulized ipratropium bromide to nebulized albuterol therapy in acute bronchiolitis. Pediatrics 1992;90:920-3.
112. Schuh S, Johnson DW, Callahan S, Canny G, Levison H. Efficacy of frequent nebulized ipratropium bromide added to frequent high-dose albuterol therapy in severe childhood asthma. J Pediatr 1995;126:639-45.
113. Johnson DW, Schuh S, Koren G, Jaffee DM. Outpatient treatment of croup with nebulized dexamethasone. Arch Pediatr Adolesc Med 1996;150:349-55.
114. Johnson DW, Jacobson S, Edney PC, Hadfield P, Mundy ME, Schuh S. A comparison of nebulized budesonide, intramuscular dexamethasone, and placebo for moderately severe croup. N Engl J Med 1998;339:498-503.
115. Schuh S, Johnson DW, Stephens D, Callahan S, Winders P, Canny GJ. Comparison of albuterol delivered by a metered dose inhaler with spacer versus a nebulizer in children with mild acute asthma. J Pediatr 1999;135:22-7.
116. Schuh S, Reisman J, Alshehri M, et al. A comparison of inhaled fluticasone and oral prednisone for children with severe acute asthma. N Engl J Med 2000;343:689-94.
117. Schuh S, Coates AL, Binnie R, et al. Efficacy of oral dexamethasone in outpatients with acute bronchiolitis. J Pediatr 2002;140:27-32.
118. Scolnik D, Coates AL, Stephens D, Da Silva Z, Lavine E, Schuh S. Controlled delivery of high vs low humidity vs mist therapy for croup in emergency departments: a randomized controlled trial. JAMA 2006;295:1274-80.

119. Schuh S, Dick PT, Stephens D, et al. High-dose inhaled fluticasone does not replace oral prednisolone in children with mild to moderate acute asthma. Pediatrics 2006;118:644-50.
120. Bloch H, Silverman R, Mancherje N, Grant S, Jagminas L, Scharf SM. Intravenous magnesium sulfate as an adjunct in the treatment of acute asthma. Chest 1995;107:1576-81.
121. Rowe BH, Bretzlaff JA, Bourdon C, Bota GW, Camargo CA, Jr. Intravenous magnesium sulfate treatment for acute asthma in the emergency department: a systematic review of the literature. Annals of emergency medicine 2000;36:181-90.

Appendix A: Pediatric Respiratory Assessment Measure (PRAM) Score

Signs	0	1	2	3
Suprasternal retractions	Absent		Present	
Scalene muscle contraction	Absent		Present	
Air entry*	Normal	Decreased at bases	Widespread decrease	Absent/minimal
Wheezing*	Absent	Expiratory only	Inspiratory and expiratory	Audible without stethoscope/ silent chest with minimal air entry
O2 saturation	≥95% - Toronto<br>≥93% - Calgary<br>>94% - Edmonton	92%-94% - Toronto<br>90%-92% - Calgary<br>90-93% - Edmonton	<92% - Toronto<br>< 90% - Calgary<br>≤89% - Edmonton	

*If asymmetric findings between the right and left lungs, the most severe side is rated. Reprinted from The Journal of Pediatrics, Vol 137, Issue 6, Chalut DS, Ducharme FM, Davis, GM. The Preschool Assessment Measure (PRAM): A responsive index of acute asthma severity. Pages 762-768, Copyright © 2000 with permission from Elsevier.

Appendix B: **Pediatric Respiratory Assessment Measure (PRAM) Scores in Triage and After Initial Bronchodilator Therapy***

**Triage PRAM**: (N)		**Post-Bronchodilator Therapy PRAM ≥ 5**
4:	74	16 (22%)
5:	69	24 (35%)
6:	88	45 (51%)
7:	50	34 (68%)
8:	32	25 (78%)
9:	18	15 (83%)
10:	10	8 (80%)
11:	11	11 (100%)

Of children with PRAM ≥5 in triage, 58% (162/278) have post-bronchodilator therapy PRAM of ≥ 5.

Probability of Hospitalization with different post-bronchodilator therapy PRAM scores*

PRAM ≥ 4:	61/290 = 21%
PRAM ≥ 5:	53/184 = 30%
PRAM ≥ 6:	45/113 = 40%

Post-Bronchodilator PRAM score as a Proportion of Asthma Hospitalizations*

PRAM ≥ 4:	97%
PRAM ≥ 5:	84%
PRAM ≥ 6:	71%
PRAM ≥ 7:	49%

*2006 Asthma Audit from a Canadian pediatric ED

Magnesium Nebulization Utilization in Management of Pediatric Asthma – "MagNUM PA"

Appendix C: LOGISTICS OF BLINDING AND KIT MAKING

	**Investigational Drug or Placebo (mg=mL) (provided in a blinded vial)**	**Salbutamol Nebulizer Solution 5mg/mL (mg=mL)**	**Diluent Volume to Top up to 6mL Final Volume (mL)**	Osmolarity (mOsm/L)
Active Arm	Magnesium Sulfate Injection 500mg/mL (600mg Mg Sulf = 1.2mL)	5mg = 1mL	Sterile Water for Injection (3.8mL)	**384**
Placebo Arm	Hypertonic Saline (5.5%) (0mg Mg Sulf = 1.2mL)	5mg = 1mL	Sterile Water for Injection (3.8mL)	**381**

Each site will prepare consecutively numbered randomization kits, numbered according to the site's Master Randomization table. Each kit will contain:
Magnesium Sulfate Injection 500mg/mL OR **Hypertonic Saline** (5.5%)

Active kits will contain Magnesium Sulfate injection
- Injection to be administered by nebulized inhalation
- Unblinded site pharmacy will repackage small batches of Canadian commercial Magnesium injection into empty sterile vials in a laminar air flow hood according to detailed worksheet procedures in the Pharmacy Manual of Operations.

Placebo Kits will contain Hypertopic Saline 5.5%
- Unblinded site pharmacy will compound small batches of Hypertonic Saline (5.5%) in a Laminar Air Flow hood using 14.6% concentrated Sodium Chloride and sterile water according to detailed worksheet procedures in the Pharmacy Manual of Operations.
- ***Hypertonic Saline (5.5%) was chosen as the Placebo since Magnesium Sulfate is hypertonic. 5.5% is the percentage that mimics the osmolality of the Active arm when sterile water is used as the top up diluent.***

The repackaged Magnesium Sulfate and compounded placebo vials will be given a 6-month expiry date.
During Kit assembly by the site pharmacy, identical labels will be placed on the blinded vials in order to ensure the integrity of the blind.

Blinded Numbered Randomization Kits will be assembled by the unblinded site pharmacy and made available to the Emergency Study RNs for use once a subject is eligible to be randomized.

Open Label supplies of the following will be available:
1. Salbutamol Nebulizer Solution 5mg/mL Canadian commercial supply. No blinding required. Drug accountability according to Health Canada Division 5 regulations will be maintained.

2. Sterile Water for Injection (SWI)
Used as the diluent to top up to final 6mL nebulizer volume
Canadian commercial supply. No blinding required.
Drug accountability according to Health Canada Division 5 regulations will be maintained
Sterile Water was chosen as the top up diluent to ensure that <u>the final osmolality of the nebulizer solutions was less than 500</u> (the osmolality at which bronchospasm has been reported). <u>The inhalation solutions in both study arms will be of comparable isotonicity.</u>

In this Investigator initiated study, the numbered kits will be assembled and labeled in the local Research Pharmacy according to detailed kit making Standard Operating Procedures provided by the Coordinating Pharmacy at SickKids. All kits/products will have appropriate Clinical Trial labeling according to Canadian regulations.

Appendix D: EMERGENCY UNBLINDING PROCEDURES

In the unlikely event the patient develops hypotension requiring therapy, apnea, heart block or another adverse event and the ED physician feels that the experimental therapy cannot be safely continued, further doses of the experimental treatment will be stopped.

If these adverse events are accompanied by severe distress and additional IV Mg is warranted, the study may be unblinded for that subject. If the subject was allocated to the Active Mg Sulfate arm, then additional IV Mg should not be given but alternative treatment provided instead. If the subject was allocated to the Placebo arm, then IV Mg may be given as part of treatment of the adverse event.

Emergency unblinding should only be requested when the clinical treatment of the patient will be different by knowing which arm of the study the patient was previously on. The study PI/local PI and the study nurses will remain blinded if possible.

The following Emergency Unblinding procedure will be followed:

1. Treating Physician or RN should contact the local PI of the study for consultation to unblind. In the event they cannot be reached immediately go to the next step.
2. Contact the SickKids hospital pharmacy by phone.
3. Provide the patient’s study randomization number, reason for unblinding, your site and your name to the SickKids pharmacist who will then provide the unblinded study arm.
4. Note that all patients whose therapy is unblinded must stop taking the experimental therapy The ED physician will prescribe additional treatment as clinically appropriate.
5. The requesting physician should initiate Email communication within 24 hours detailing the request for Emergency unblinding and why. The email must inform the local PI and SickKids Research Pharmacist and Study PI.
6. The local DSMC and REB will be advised of emergency unblinding within 48 hours.

Appendix E: ANNUAL ENROLLMENT PROJECTION

**Site**	**Annual Asthma Presentations ≥2 years of age**	**Projected Annual Screens+**	**Projected Randomizations***	**Projected Annual Study Completion based on progress to date and asthma presentations**
Hospital for Sick Children	682	340	44	40
Children Hospital of Eastern Ontario	672	336	43	39
Alberta Children's Hospital	660	330	42	38
Stollery Children's Hospital	320	160	20	18
Winnipeg Children's Hospital	500	250	32	29
CHU – Sainte-Justine	670	335	43	39
BC Children's Hospital	490	245	31	28
Total	**3994**	**1996**	**255**	**231**

+ screens represent approximately 50% of annual presentations as per current study
***** randomizations represent 13% of patients screened as per progress in current study

Magnesium Nebulization Utilization in Management of Pediatric Asthma – “MagNUM PA”

Current Seasonal Patient Accrual and Progress to date

Annual presentations at SickKids and ACH: 1337

↓

Available for screening: 718 (54%)

↓

Misses: 46 (6.5%)

↓

Screened: 672

↓

Exclusions: 558

↓

Eligible: 114 (17%)

↓

Refusals: 27 (24%)

↓

Randomized: 87

↓

Completed experimental Rx: 87 (100%)

↓

Follow up completed: 87 (100%)

Not screened (RNs off duty): 619

Exclusions:

PRAM <5 in triage/after Rx: 324

First wheeze: 68

Pneumonia 14

Co-morbidities:98

Transferred on IV Mg: 14

Allergy to Mg: 1

No English: 3

Previous enrollment: 6

Other reasons: 30

Magnesium Nebulization Utilization in Management of Pediatric Asthma – "MagNUM PA"

Expected annual patient accrual based on asthma presentations to participating EDs and study progress to date

Annual presentations: 3994 (September through May)

↓

Available for screening (54%): 2156

↓

Missed (7%): 150

↓

Screened: 2006

↓

Exclusions: 1664

↓

Eligible: 342 (17%)

↓

Refusals: 82 (24%)

↓

Randomized: 260

↓

Complete experimental Rx: 246 (95%)

↓

Complete follow up: 234 (95%)
