# Supplementary material for: Association Between Intravenous Magnesium Therapy in the Emergency Department and Subsequent Hospitalization Among Pediatric Patients With Refractory Acute Asthma: Secondary Analysis of a Randomized Clinical Trial
Source: JAMA Netw Open. 2021 Jul 19;4(7):e2117542. doi: 10.1001/jamanetworkopen.2021.17542 (PMC8290299; doi:10.1001/jamanetworkopen.2021.17542)
Supplement: Supplement 2. — Nonauthor Collaborators. Pediatric Emergency Research Canada [file jamanetwopen-e2117542-s002.pdf]

Supplemental Online Content: Nonauthor Collaborators

*Indicates required information. Only first name, last name, and suffix will appear in PubMed.

*Group Name(s): Pediatric Emergency Research Canada							
***First Name and Middle Initial(s)**	***Last Name**	***Suffix (eg, Jr, III)**	**Academic Degrees**	**Institution**	**Location (city, state/province, country)**	**Role or Contribution, eg, chair, principal investigator**	**Group (if more than 1 Group listed in the byline) and/or Subgroup (eg, Steering Committee)**
Stephen B.	Freedman		MDCM, MSc	Sections of Pediatric Emergency Medicine and Gastroenterology, Departments of Pediatrics and Emergency Medicine, Alberta Children's Hospital, Alberta Children's Hospital Research Institute, Cumming School of Medicine, University of Calgary,	Calgary, Alberta, Canada	Protocol review, PERC facilitation, guidance and support	Past Chair
Roger	Zemek		MD	Department of Pediatrics and Emergency Medicine, Children's Hospital of Eastern Ontario, University of Ottawa; CHEO Research Institute	Ottawa, Ontario, Canada	Protocol review, PERC facilitation, guidance and support	Chair
Samina	Ali		MDCM	Department of Pediatrics; Division of Pediatric Emergency Medicine, University of Alberta	Edmonton, Alberta, Canada	Protocol review, PERC facilitation, guidance and support	Vice Chair
Maala	Bhatt		MD, MSc	Emergency Medicine Physician and Associate Professor, Department of Pediatrics, University of Ottawa	Ottawa, Ontario, Canada	Guidance, study review, support	Executive Board Member
Amanda (Mandi)	Newton		PhD	Department of Pediatrics, Faculty of Medicine & Dentistry, University of Alberta	Edmonton, Alberta, Canada	Guidance, study review, support	Executive Board Member
Garth	Meckler		MD, MSHS	Division of Pediatric Emergency Medicine, BC Children's Hospital	Vancouver, British Columbia, Canada	Guidance, study review, support	Executive Board Member

Supplemental Online Content: Nonauthor Collaborators

*Indicates required information. Only first name, last name, and suffix will appear in PubMed.

*First Name and Middle Initial(s)	*Last Name	*Suffix (eg, Jr, III)	Academic Degrees	Institution	Location (city, state/province, country)	Role or Contribution, eg, chair, principal investigator	Group (if more than 1 Group listed in the byline) and/or Subgroup (eg, Steering Committee)
Naveen	Poonai		MD, MSc	Lawson Health Research Institute, London Health Sciences Centre	London, Ontario, Canada	Guidance, study review, support	Executive Board Member
Candice	McGahern		BA	Children's Hospital of Eastern Ontario	Ottawa, Ontario, Canada	Guidance, study review, support	Coordinator Representative
Kate	Maki		MD	BC Childrens Hospital	Vancouver, British Columbia, Canada	Guidance, study review, support	Fellows' Representative
Rebecca	Emerton		BA(Hons)	Alberta Children's Hospital	Calgary, Alberta, Canada	Guidance, PERC facilitation, study review, support	Network Coordinator
